# Supplementary material for: Effect of Dietary Supplements Which Upregulate Nitric Oxide on Walking and Quality of Life in Patients with Peripheral Artery Disease: A Meta-Analysis
Source: Biomedicines. 2023 Jun 29;11(7):1859. doi: 10.3390/biomedicines11071859 (PMC10376856; doi:10.3390/biomedicines11071859)

Section S1:
Supplementary Methods: PRISMA 2020 Checklist

Section and Topic	Item #	Checklist item	Location where item is reported
**TITLE**			
Title	1	Identify the report as a systematic review.	1
**ABSTRACT**			
Abstract	2	See the PRISMA 2020 for Abstracts checklist.	2
**INTRODUCTION**			
Rationale	3	Describe the rationale for the review in the context of existing knowledge.	5
Objectives	4	Provide an explicit statement of the objective(s) or question(s) the review addresses.	5
**METHODS**			
Eligibility criteria	5	Specify the inclusion and exclusion criteria for the review and how studies were grouped for the syntheses.	6
Information sources	6	Specify all databases, registers, websites, organisations, reference lists and other sources searched or consulted to identify studies. Specify the date when each source was last searched or consulted.	5
Search strategy	7	Present the full search strategies for all databases, registers and websites, including any filters and limits used.	Section S1
Selection process	8	Specify the methods used to decide whether a study met the inclusion criteria of the review, including how many reviewers screened each record and each report retrieved, whether they worked independently, and if applicable, details of automation tools used in the process.	6
Data collection process	9	Specify the methods used to collect data from reports, including how many reviewers collected data from each report, whether they worked independently, any processes for obtaining or confirming data from study investigators, and if applicable, details of automation tools used in the process.	7
Data items	10a	List and define all outcomes for which data were sought. Specify whether all results that were compatible with each outcome domain in each study were sought (e.g. for all measures, time points, analyses), and if not, the methods used to decide which results to collect.	6
	10b	List and define all other variables for which data were sought (e.g. participant and intervention characteristics, funding sources). Describe any assumptions made about any missing or unclear information.	7
Study risk of bias assessment	11	Specify the methods used to assess risk of bias in the included studies, including details of the tool(s) used, how many reviewers assessed each study and whether they worked independently, and if applicable, details of automation tools used in the process.	7
Effect measures	12	Specify for each outcome the effect measure(s) (e.g. risk ratio, mean difference) used in the synthesis or presentation of results.	7, 8
Synthesis methods	13a	Describe the processes used to decide which studies were eligible for each synthesis (e.g. tabulating the study intervention characteristics and comparing against the planned groups for each synthesis (item #5)).	7, 8
	13b	Describe any methods required to prepare the data for presentation or synthesis, such as handling of missing summary statistics, or data conversions.	7
	13c	Describe any methods used to tabulate or visually display results of individual studies and syntheses.	8
	13d	Describe any methods used to synthesize results and provide a rationale for the choice(s). If meta-analysis was performed, describe the model(s), method(s) to identify the presence and extent of statistical heterogeneity, and software package(s) used.	7, 8
	13e	Describe any methods used to explore possible causes of heterogeneity among study results (e.g. subgroup analysis, meta-regression).	8
	13f	Describe any sensitivity analyses conducted to assess robustness of the synthesized results.	8
Reporting bias assessment	14	Describe any methods used to assess risk of bias due to missing results in a synthesis (arising from reporting biases).	8
Certainty assessment	15	Describe any methods used to assess certainty (or confidence) in the body of evidence for an outcome.	7, 8
**RESULTS**			
Study selection	16a	Describe the results of the search and selection process, from the number of records identified in the search to the number of studies included in the review, ideally using a flow diagram.	8, Figure 2
	16b	Cite studies that might appear to meet the inclusion criteria, but which were excluded, and explain why they were excluded.	32
Study characteristics	17	Cite each included study and present its characteristics.	8, Table 1, Tables S1 and S2
Risk of bias in studies	18	Present assessments of risk of bias for each included study.	Figure 3, Table S3
Results of individual studies	19	For all outcomes, present, for each study: (a) summary statistics for each group (where appropriate) and (b) an effect estimate and its precision (e.g. confidence/credible interval), ideally using structured tables or plots.	Figures 4–6, Table S4, Figures S2–S32
	20a	For each synthesis, briefly summarise the characteristics and risk of bias among contributing studies.	Figure S1

Section and Topic	Item #	Checklist item	Location where item is reported
Results of syntheses	20b	Present results of all statistical syntheses conducted. If meta-analysis was done, present for each the summary estimate and its precision (e.g. confidence/credible interval) and measures of statistical heterogeneity. If comparing groups, describe the direction of the effect.	9-14
	20c	Present results of all investigations of possible causes of heterogeneity among study results.	9-14
	20d	Present results of all sensitivity analyses conducted to assess the robustness of the synthesized results.	9-14, Tables S5–S15
Reporting biases	21	Present assessments of risk of bias due to missing results (arising from reporting biases) for each synthesis assessed.	Table S4
Certainty of evidence	22	Present assessments of certainty (or confidence) in the body of evidence for each outcome assessed.	9-14
**DISCUSSION**			
Discussion	23a	Provide a general interpretation of the results in the context of other evidence.	14
	23b	Discuss any limitations of the evidence included in the review.	15-16
	23c	Discuss any limitations of the review processes used.	15-16
	23d	Discuss implications of the results for practice, policy, and future research.	16
**OTHER INFORMATION**			
Registration and protocol	24a	Provide registration information for the review, including register name and registration number, or state that the review was not registered.	5
	24b	Indicate where the review protocol can be accessed, or state that a protocol was not prepared.	5
	24c	Describe and explain any amendments to information provided at registration or in the protocol.	N/A
Support	25	Describe sources of financial or non-financial support for the review, and the role of the funders or sponsors in the review.	16
Competing interests	26	Declare any competing interests of review authors.	16
Availability of data, code and other materials	27	Report which of the following are publicly available and where they can be found: template data collection forms; data extracted from included studies; data used for all analyses; analytic code; any other materials used in the review.	16

Supplementary Methods: Search Strategy

OVID Database:
Ovid MEDLINE(R) and Epub Ahead of Print, In-Process, In-Data-Review & Other Non-Indexed Citations, Daily and Versions(R) <1946 to July 07, 2021>

#	**Query**
1	(Nitr*te adj3 (diet* or oral or inorganic or supplement* or exogenous)).mp.
2	(Nitric oxide adj3 (diet* or oral or inorganic or supplement* or exogenous)).mp.
3	(antioxidant or anti-oxidant or flavon* or flavan* or neutraceutical* or nutraceutical* or nutriceutical* or neutriceutical*).mp.
4	(NOX2 or NOX-2 or "NADPH oxidase" or "nitric oxide").mp.
5	((NOX2 or NOX-2) adj3 (regulation or downregulation or down-regulation)).mp.
6	"Fruit and vegetable juices"/
7	(Beet* or garlic or arginine or citrulline or carnitine or "coenzyme Q10" or "coq10" or "co-enzyme q10" or cacao or cocoa or catechin* or "vitamin C" or polyphenol* or epicatechin* or catechin*).mp.
8	1 or 2 or 3 or 4 or 5 or 6 or 7
9	((peripheral adj2 arter* adj2 disease) or (peripheral adj2 vascular adj2 disease)).mp.
10	(claudication adj3 (intermittent or leg or limb or peripheral)).mp.
11	((ischemia or ischaemia) adj3 (limb or leg or peripheral)).mp.
12	9 or 10 or 11
13	8 and 12
14	limit 13 to (adaptive clinical trial or clinical trial, all or clinical trial or comparative study or controlled clinical trial or meta analysis or multicenter study or observational study or pragmatic clinical trial or randomized controlled trial or "systematic review")

CINAHL Database:

#	**Query**
1	((MH “Nitrogen Oxides”) OR (MH “Nitric Oxide”) OR (MH “Nitric Oxide Synthases”) OR nitrate supplementation OR nitric oxide OR nitric oxide therapy OR nitrite OR antioxidant supplements OR antioxidants OR nutraceuticals OR (MH “Dietary Supplements”) OR (MH “Dietary Supplementation”) OR cacao OR cocoa OR flavanol OR flavonol OR beet OR beetroot OR vegetable OR garlic OR arginine OR citrulline OR carnitine OR polyphenol)
2	((MH “Peripheral Vascular Diseases”) OR (MH “Arterial Occlusive Diseases”))
3	1 AND 2
4	Limit to clinical trials and RCTs

SCOPUS Database:

#	**Query**
1	((TITLE-ABS-KEY ("peripheral arterial disease" OR "peripheral arterial diseases" OR "peripheral artery disease" OR "peripheral artery diseases" OR "arterial obstructive disease" OR "arterial obstructive diseases" OR "arterial occlusive disease" OR "arterial occlusive diseases" OR "limb ischemia" OR "limb ischemias" OR "limb ischaemia" OR "limb ischaemias" OR "intermittent claudication")) **AND**
2	TITLE-ABS-KEY (nitrate OR nitrite OR nitrates OR nitrites OR "nitric oxide" OR antioxidant OR anti-oxidant OR cacao OR cocoa OR flavanol OR flavonol OR beet OR beetroot OR vegetable OR garlic OR arginine OR citrulline OR carnitine OR polyphenol) **OR**
3	(TITLE-ABS-KEY (nitrate OR nitrite OR nitrates OR nitrites OR "nitric oxide" OR antioxidant OR anti-oxidant OR cacao OR cocoa OR flavanol OR flavonol OR beet OR beetroot OR vegetable OR garlic OR arginine OR citrulline OR carnitine OR polyphenol)))) **AND**
4	((INDEXTERMS ("clinical trials" OR "clinical trials as a topic" OR "randomized controlled trial" OR "Randomized Controlled Trials as Topic" OR "controlled clinical trial" OR "Controlled Clinical Trials" OR "random allocation" OR "Double-Blind Method" OR "Single-Blind Method" OR "Cross-Over Studies" OR "Placebos" OR "multicenter study" OR "double blind procedure" OR "single blind procedure" OR "crossover procedure" OR "clinical trial" OR "controlled study" OR "randomization" OR "placebo")) **OR**
5	(TITLE-ABS-KEY (("clinical trials" OR "clinical trials as a topic" OR "randomized controlled trial" OR "Randomized Controlled Trials as Topic" OR "controlled clinical trial" OR "Controlled Clinical Trials as Topic" OR "random allocation" OR "randomly allocated" OR "allocated randomly" OR "Double-Blind Method" OR "Single-Blind Method" OR "Cross-Over Studies" OR "Placebos" OR "cross-over trial" OR "single blind" OR "double blind" OR "factorial design" OR "factorial trial"))) **OR**
6	(TITLE-ABS (clinical AND trial* OR trial* OR rct* OR random* OR blind*)))

Supplementary Methods: Method for imputing missing data

Missing endpoint data was imputed using data at baseline and change from baseline reported in the studies.

The standard equation for estimating the standard deviation of a pre-post difference from the standard deviations of pre and post is:

$sd_{change}=\sqrt{[(sd^2_{Pre}+ sd^2_{Post})-(2\times Corr \times sd_{Pre}\times sd_{Post})]}$

Therefore, squaring both sides

$sd^2_{change} = (sd^2_{Pre}+ sd^2_{Post})-(2\times Corr \times sd_{Pre}\times sd_{Post})$

$= sd^2_{Pre}+ sd^2_{Post} - 2\times Corr \times sd_{Pre}\times sd_{Post}$[24]

Then rearranging the equation:

$sd^2_{Post} + sd^2_{Pre} - sd^2_{change} - 2\ Corr\ sd_{Pre}\ sd_{Post} = 0$

$sd^2_{Post} - 2\ Corr\ sd_{Pre}\ sd_{Post} + sd^2_{change} - sd^2_{Pre} = 0$

Let $sd_{Post} = x$

Then in the equation $ax^2 + bx + c = 0$

$a = 1$

$b = - 2\times Corr \times sd_{Pre}$

$c = sd^2_{change} - sd^2_{Pre}$

If Corr, sd_{Pre}, and sd_{change} are all known or can be estimated, the quadratic equation $ax^2 + bx + c = 0$ can (potentially) be solved for sd_{Post}, via

$sd_{Post} = (- b +/- sqrt(b^2 - 4ac))/2a$

(If the value of $b^2 - 4ac$ is negative, the estimate of sd_{Post} is not a real number, so the solution cannot be used.)

Using this approach needs the correlation coefficient (Corr) between individual Pre and Post data values to be estimated from the data in each of the four datasets.

Fortunately, each data set included some studies which provided both sd_{change} and sd_{Post}. For these studies, sd_{Post} was estimated repeatedly from sd_{Pre} and sd_{change} using a range of values of Corr starting at 0 and with increments of 0.1. The value of Corr which resulted in the smallest average error in estimates of sd_{Post} was then used to estimate sd_{Post} in studies where sd_{Post} had not been provided by the authors.
In cases where the resulting sd_{Post} could not be estimated because the value of $b^2 - 4ac$ proved to be negative, an alternative approach was used. In studies which reported both sd_{Pre} and sd_{Post}, the two values were moderately well correlated, though there was a tendency for standard deviations to be higher for sd_{Post}, and for the values to be more variable as the standard deviation became larger (for examples see the two figures below, where the slope of the line represents the average ratio of sd_{Post} to sd_{Pre}).

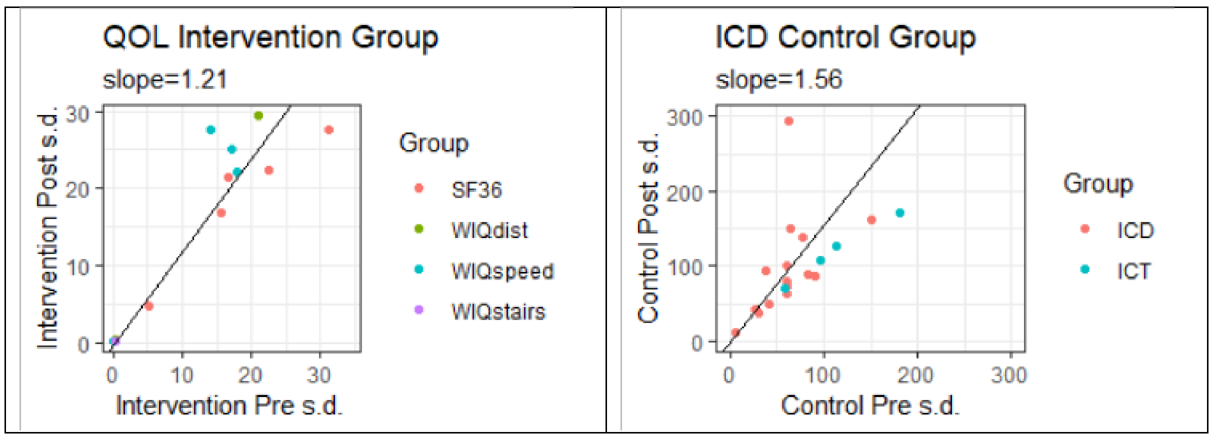

Estimated ratios for each group are then:

ICD Control	1.56
ICD Intervention	2.27
ABI Control	1.31
ABI Intervention	1.02
MWD Control	1.73
MWD Intervention	2.40
QOL Control	1.09
QOL Intervention	1.21

This gives an alternative approach to estimating values of sd_{Post} in studies where its value was not provided, as the value of sd_{Pre} multiplied by the relevant ratio.

Section S2

Table S1. Description of study interventions and outcomes.

Study	Description of intervention	Description of control	Primary outcome(s)	Secondary Outcome(s)	Assessments of adherence
**Nitric Oxide Donors**					
Bock (2018)	1g/day of $NaNO_3$ (sodium nitrate) for 8 weeks.	Matched placebo for 8 weeks (microcrystalline cellulose)	1. Peak calf blood flow (change in vasodilator capacity)<br>2. Peak calf vascular conductance (change in arterial stiffness)<br>3. Six-minute walk test distance	1. Plasma inflammatory and adhesion biomarkers<br>2. Plasma nitrate and nitrite concentrations	Pill counting and blood samples for plasma nitrate and nitrite.
Gresele (2012)	800mg twice daily of NCX 4016 for 6 months.	Matched placebo for 6 months	1. Maximum walking distance	1. Initial claudication distance<br>2. Quality of life<br>3. ABI	Pill counting.
Kenjale (2011)	Single 500mL bottle of NO_3^- rich beetroot juice.	Single 500mL bottle of orange juice.	1. Plasma NO_2^- concentration<br>2. Exercise tolerance	1. BP<br>2. Heart rate<br>3. VO2 responses	NA
Mohler (2014)	40mg twice daily or 80mg twice daily of sodium nitrite tablets for 10 weeks, followed by 80mg twice daily or 160mg twice daily for 1 week.	Matched placebo for 11 weeks.	1. Safety & tolerability of intervention	1. Endothelial function and markers of functional improvement<br>2. Six-minute walk test distance<br>3. Quality of life	Pill counting.
Pekas (2021)	Single dose of a beetroot supplement (0.11mmol NO_3^- per kg bodyweight). Washout between groups was 14 days.	Matched placebo: tapioca powder capsules.	NR	NR	NA
Van der Avoort (2021)	Intervention 1: nitrate rich vegetable meal containing 400mg dietary nitrate – green smoothie (200mL) containing mango, arugula, zucchini, orange juice, and rhubarb compote + 2 beetroot waffles (200g) containing egg, wholemeal flour, low-fat milk, and beetroots.<br>Intervention 2: 70mL concentrated red beetroot juice (Beet-it Sport ®).<br>Washout between interventions was 7-14 days.	Matched placebo: 70mL nitrate-depleted Beet-it Sport ®	1. Exercise tolerance (as measured by claudication onset time and maximal walking time)	1. Plasma nitrate and nitrite concentrations<br>2. Muscle oxygenation as assessed by near-infrared spectroscopy<br>3. Blood pressure<br>4. Arterial stiffness (pulse wave velocity)	NA
Woessner (2018)	70mL (4.2 mmol NO_3^-) beetroot juice 3 hours prior to each supervised exercise session (3 times per week for 12 weeks). Exercise sessions included at least 30 minutes of actual walking, with intensity tailored to each subjects' initial baseline maximal graded exercise test results.	Matched placebo: 70mL of nitrate-depleted placebo 3 hours prior to each supervised exercise session. Exercise sessions were identical to those in the intervention group.	1. Change in exercise capacity (pain-free walking time, peak walking time, VO2 peak)<br>2. Six-minute walk test distance	1. Gastrocnemius tissue oxygenation<br>2. Gastrocnemius muscle angiogenesis<br>3. Vascular function	Several scheduled and unannounced blood samples were taken throughout the study.
**Enhancers of NO Availability**					
Domingues (2021)	Creatinine monohydrate supplementation (20g/day [5g four times daily] for 1 week, then 5g once daily for 7 weeks.	Matched placebo for 8 weeks (dextrose).	1. Six-minute walk test distance	1. Upper-limb strength<br>2. Lower-limb strength<br>3. Calf-muscle StO2	Plasma creatinine levels in a subset of the participants.
Maxwell (2000)	One or two L-arginine enriched nutrient bars daily, with each 50g bar containing 3.3g L-arginine, in addition to antioxidant vitamins and minerals, folic acid, and B-complex vitamins for 2 weeks.	Matched placebo bars for 2 weeks (arginine-poor whey rather than soy based)	1. Initial claudication distance	1. Maximum walking distance<br>2. Quality of life	NR
Micker (2007)	4g three times daily of L-arginine tablets for 1 month. The first seven days were completed in hospital.	Matched placebo for 1 month	1. Initial claudication distance<br>2. Maximum walking distance	NR	NR

Oka (2005)	9g daily of L-arginine in three divided doses for 3 months.	Matched placebo for 3 months.	NR	NR	NR
Wilson (2007)	1g L-arginine three times daily for 6 months.	Matched placebo for 6 months.	1. Absolute claudication distance	1. Initial claudication distance<br>2. Functional status<br>3. ABI	Pill counting and blood samples for plasma arginine.
**Nitric Oxide Synthase Inducers**					
Loffredo (2014)	One dose of 40mg of dark chocolate (>85% cocoa). Washout between the intervention and control was 1 week.	One dose of milk chocolate (<35% cocoa).	1. Flow-mediated dilation<br>2. Maximum walking distance	1. Maximum walking time<br>2. ABI<br>3. Oxidative stress markers	NR
McDermott (2017)	Daily capsules of resveratrol, 125 mg; or resveratrol, 500 mg (both 98% pure trans-resveratrol; Reserveage Nutrition) for 6 months	Matched placebo capsules for 6 months	1. Change in six-minute walk test distance	1. Change in maximal walking time<br>2. Change in pain-free walking time<br>3. Brachial artery flow-mediated dilation<br>4. Calf muscle biopsy measures of peroxisome proliferator-activated receptor γ coactivation 1^{α}, COX activity and citrate synthase activity	Pill counting.
McDermott (2020)	Flavanol-rich cocoa powder packets, with one packed to be mixed with water or milk three times daily for 6 months.	Matched placebo packets for 6 months.	1. Six-minute walk test distance (at 6 months – 2.5 hours and 24 hours after a dose of intervention)	1. Brachial artery flow-mediated dilation<br>2. Maximum walking distance<br>3. Initial claudication distance	Monthly packet counting.
Tenore (2019)	2g Annurca apple polyphenolic extract daily (two 500mg capsules twice daily) for 6 months.	Matched placebo for 6 months (maltodextrin).	1. Walking autonomy in IC<br>2. Haemodynamic parameters (ABI and acceleration time)	1. Vascular abnormalities of the lower limbs	Pill log.
**Antioxidants**					
Brevetti (1988)	2g L-carnitine twice daily for 3 weeks. Washout between the intervention and control was 1 week.	Matched placebo tablets for 3 weeks.	1. Initial claudication distance	1. Subjective symptoms<br>2. Metabolic assessment	NR
Brevetti (1995)	Increasing dose of propionyl-L-carnitine from 500mg twice daily to 2g daily then 3g daily at 2-month intervals in patients showing improvement in treadmill performance <30% over baseline (total of 24 weeks). Patients showing improvement >30% over baseline continued with the same dose as in the previous 2 months.	Placebo for 24 weeks	1. Initial claudication distance<br>2. Maximum walking distance	1. Quality of life (reported in Brevetti 1997)	Pill counting (blinded to participants). Patients taking < 70% of their prescribed dose were considered noncompliant and excluded from efficacy analysis.
Brevetti (1999)	1g twice daily of propionyl-L-carnitine.	Placebo	1. Maximum walking distance	1. Initial claudication distance<br>2. Quality of life	Pill counting (blinded to participants). Patients taking <75% of the prescribed dose were non-compliant and considered dropouts.
Collins (2003)	400IU vitamin E daily (single capsule) for 6 months	Matched placebo capsule for 6 months (oil).	1. Walking ability<br>2. Quality of life	NR	Patient self-reporting and measured vitamin E levels.
Coto (1992)	Propionyl L-carnitine (2g administered per os daily in two divided doses).	Placebo (2g administered per os daily in two divided doses).	1. Initial claudication distance<br>2. Maximum walking distance	NR	Patient self-reporting and pill counting.
Da Silva (2015)	1.8g/day N-acetylcysteine for 4 days. Washout between the intervention and control was at least 10 days.	Placebo for 4 days.	1. Initial claudication distance<br>2. Maximum walking distance	NR	NR
Dal Lago (1999)	1g three times daily of propionyl-L-carnitine	Placebo	1. Initial claudication distance<br>2. Maximum walking distance	NR	Pill counting.
Deckert (1997)	1g daily of propionyl-L-carnitine, increasing up to 3g daily if improvements in treadmill performance occurred.	Placebo	1. Quality of life	NR	Pill counting.

Gardner (2008)	180mg Gingko Biloba (EGb 761) in the morning and 120mg at night (300mg daily).	Matched placebo for 4 months (dextrose).	1. Maximum walking time<br>2. Initial claudication time	1. Flow-mediated vasodilation<br>2. Antioxidant status<br>3. Walking impairment<br>4. Quality of life	Pill counting.
Goldenberg (2012)	100mg twice daily of cilostazol plus 1g twice daily L-carnitine for 6 months.	100mg twice daily of cilostazol plus matched placebo twice daily	1. Maximum walking time	1. Initial claudication time<br>2. Quality of life	Pill counting.
Grenon (2015)	2.2g n-3 polyunsaturated fatty acid (fish oil) twice daily for one month; four capsules twice daily, total of 2.6g EPA and 1.8g of DHA daily	Matched placebo for one month.	1. Change in endothelial function	1. Inflammatory markers<br>2. Lipid profile<br>3. Blood pressure<br>4. Quality of life	NR
Hiatt (2011)	2g daily of propionyl-L-carnitine for 6 months in addition to a monitored exercise training regimen including 50 minutes of treadmill walking monthly, and instructions on similar exercises at home three times per week.	Matched placebo and monitored exercise training for 6 months	1. Maximum walking time	1. Initial claudication time<br>2. Quality of life	Accelerometer and diary to monitor home exercises, and pill counting for adherence to propionyl-L-carnitine.
Kiesewetter (1993)	Two coated tablets containing 200mg standardized garlic powder twice daily for 3 months.	Matched placebo for 3 months.	1. Initial claudication distance	1. Blood pressure<br>2. Heart rate<br>3. ABI<br>4. Lipid levels<br>5. Plasma viscosity and thrombocyte aggregation<br>6. Adverse events	NR
Leng (1997)	One antioxidant capsule daily for 24 months containing 3mg beta-carotene, 100mg ascorbic acid, 25mg pyridoxine hydrochloride, 100mg zinc sulphate, 10mg nicotinamide, and 1mg sodium selenite.	Matched placebo for 24 months (255mg coconut oil).	1. Plasma cholesterol, lipoprotein<br>2. Haemostatic and rheological factors	1. ABI<br>2. Walking distance	NR
Luo (2013)	Two tablets twice daily of propionyl-L-carnitine (total of 2g daily) for 4 months.	Placebo for 4 months.	1. Maximum walking time	1. Initial claudication time<br>2. ABI	NR
Park (2020)	A single dose of 80mg MitoQ (a mitochondrial-targeted antioxidant). Washout between groups was 14 days.	Matched placebo.	1. Flow-mediated dilation	NR	NA
Ramirez (2019)	4.4g daily of n-3 polyunsaturated fatty acid (fish oil; four capsules twice daily) for 3 months. Each capsule contains 325mg EPA and 225mg DHA.	Matched placebo for 3 months (soybean).	1. Change in plasma high-sensitivity C-reactive protein	1. Biomarkers of inflammation<br>2. SPM profile<br>3. Omega-3 index<br>4. Brachial artery flow-mediated vasodilation<br>5. Measures of walking ability	Pill counting.
Santo (2006)	2g propionyl L-carnitine daily for 12 months	Matched placebo for 12 months	1. ABI<br>2. Initial claudication distance	1. Oxidative profile<br>2. Nitrates/nitrites in plasma	NR
Vincent (2007)	600mg alpha-lipoic acid daily (300mg twice daily on an empty stomach) for 3 months.	Matched placebo for 3 months (microcrystalline cellulose).	1. Six-minute walk test distance<br>2. 4-meter walk time<br>3. Initial claudication time<br>4. Initial claudication distance	NR	Pill log and pill counts.

IC = intermittent claudication; NR = not reported.

Table S2. Descriptions of the inclusion and exclusion criteria for the included studies.

Study	Inclusion criteria	Exclusion criteria	Number screened and excluded
**Nitric Oxide Donors**			
Bock (2018)	Patients aged 50-85 years with documented PAD (Fontaine Stage 1 to 2a, Rutherford 0 to 1) recruited from a single vascular clinic.	Non-atherosclerotic vascular disease, critical limb ischemia, active foot ulcers, recent revascularization (within one year), symptomatic coronary artery disease, heart failure, renal failure, resting SBP > 180 or DBP > 100, hypotension, active or recent smoker, or use of phosphodiesterase-5 inhibitors, and women with history of HRT use within the last 6 months.	NR
Gresele (2012)	Patients aged 40-80 years with documented PAD (Fontaine stage 2, ABI <0.9, and initial claudication distance >50m and absolute claudication distance <500m) and IC for at least 6 months	Unstable PAD, significant renal or hepatic failure, type 1 diabetes, uncontrolled type 2 diabetes, arterial hypertension, allergy, dyslipidaemia, any condition that would limit exercise capacity (e.g. heart failure or angina), active or recent peptic ulcer disease, any haemorrhagic condition, recent coronary or cerebrovascular episodes, recent revascularization, pregnancy or lactation.	Of 485 screened, 43 did not meet the inclusion/exclusion criteria.
Kenjale (2011)	Patients with documented PAD (ABI <0.9) and stable IC for at least 3 years from vascular clinics associated with a single university.	Previous gangrene, expected loss of limb, osteomyelitis, recent vascular surgery, angioplasty or sympathectomy, severe peripheral neuropathy, any non-PAD condition limiting walking ability, unstable angina, coronary artery disease, recent myocardial infarction, any history of hepatic or renal insufficiency, chest pain during treadmill exercise, or currently taking proton pump inhibitors,	NR
Mohler (2014)	Patients aged 35-85 years with documented PAD (ABI <0.9) with stable symptoms for at least 1 month, and unable to fall pregnant.	Non-atherosclerotic PAD, recent lower extremity surgery or revascularization, recent myocardial infarction, unstable angina, cerebrovascular accident, or transient ischaemic attack, poorly controlled diabetes, uncontrolled hypertension or hypotension, renal insufficiency, hypersensitivity to sodium nitrite, pregnant or breastfeeding, active malignancy or infection, heart failure, critical limb ischaemia, lower-limb amputation, anaemia, chronic hemolytic condition, or use of allopurinol, tricyclic antidepressants, antihistamines, CNS depressants, meperidine, and nitrates.	NR
Pekas (2021)	Patients with documented PAD (Fontaine Stage 2a and 2b; ABI <0.9) and stable blood pressure, lipid, and diabetes regimen; all females were postmenopausal.	Rest pain or tissue loss due to PAD, non-PAD walking limitation, kidney disease, already included a form of nitrate intake in their regimen, or allergy to beetroot juice.	Of 14 assessed, 3 were excluded (2 due to kidney disease).
Van der Avoort (2021)	Patients with documented PAD (ABI < 0.9) and a history of stable intermittent claudication for > 3 months (classified as Fontaine Stage IIA-III, Rutherford 1-4) recruited from vascular clinics by physician referral.	Past medical history of endovascular or surgical intervention for claudication within the last 12 months, CKD, insulin-dependent diabetes, severe peripheral neuropathy, any other condition other than PAD that limits walking, use of isosorbide dinitrite/mononitrate, sildenafil, tadalafil, or vardenafil.	Of 26 assessed, 8 were excluded (3 did not meet the inclusion criteria and 5 declined to participate).
Woessner (2018)	Patients aged 40-80 years with documented PAD (ABI <0.9) with stable IC pain as the limiting factor in their ability to exercise, no major changes in medications in the preceding three months, and recruited from a single medical center.	Any condition that could limit exercise performance (including foot ulcers, advanced neuropathy, gangrene), or impacted on safety of exercising (including recent myocardial infarction, chest pain during exercise test), type 1 diabetes or uncontrolled diabetes (HbA1c >8.5%), a major cardiovascular event in the preceding 6 weeks or a planned hospitalization within 2 months, allergy to beets or proton pump inhibitors.	NR
**Enhancers of NO Availability**			
Domingues (2021)	Patients with documented PAD (ABI <0.9) and IC experienced during the six-minute walk test, from a single tertiary vascular center.	Chronic renal insufficiency (Cr clearance <30mL/min), adverse events from creatinine supplementation, or non-compliance with study procedures.	Of 160 screened, 118 did not meet eligibility criteria, five refused participation, and five were not included for other reasons.
Maxwell (2000)	Patients with documented PAD (ABI <0.9, dropping by at least 25% during exercise) and intermittent claudication for at least 6 months.	Any non-PAD walking impairment, non-atherosclerotic artery disease, recent major surgery, lower-limb amputation above the ankle, recent myocardial infarction, type 1 diabetes, uncontrolled hypertension, active malignancy, significantly impaired renal or hepatic function, or currently enrolled in another clinical trial.	Of 156 screened, 41 met the inclusion/exclusion criteria.
Micker (2007)	Patients with documented PAD (Fontaine stage 2) from a single vascular clinic.	Serious renal or hepatic failure, diabetes, thyroid disease, electrolyte imbalance, neoplasm, systemic disease, malabsorption, psychiatric disorder, alcohol abuse, or any other condition that would risk patient safety.	NR
Oka (2005)	Patients aged at least 40 years with documented PAD (Fontaine Class 2 or 3 and ABI <0.9 with at least 25% decrease after exercise) and IC, able to walk for 2-12 minutes on a treadmill.	Non-PAD walking limitation, non-atherosclerotic PAD, Fontaine class 4, recent major cardiovascular surgery, leg amputation above the ankle, recent myocardial infarction, enrollment in another trial or recent ingestion of another investigational product, proliferative retinopathy, disease or surgery affecting gastrointestinal absorption, hepatic	Of 610 screened, 264 were initially ineligible, a further 39 did not attend the initial clinic visit, 217 were excluded during

		disease, uncontrolled hypertension, type 1 diabetes, active malignancy or tumor, serious infection or hypotension associated with sepsis, or autoimmune disease.	an initial clinic visit, and a further 10 excluded after the clinic visit.
Wilson (2007)	Patients aged at least 45 years with documented PAD (resting ABI <0.9), stable IC pain for the previous 3 months, and able to walk on a treadmill for between 1 and 12 minutes with variability of MWD between 2 consecutive screening tests < 25%.	Ischaemic pain at rest, ulceration or gangrene, recent acute coronary syndrome or revascularization, major amputation, malignancy within the past 5 years, proliferative retinopathy, uncontrolled hypertension, or active inflammation, infectious or autoimmune disease. 1 month washout was required for patients taking pentoxifylline, cilostazol, prostanoids, L-carnitine or L-arginine.	Of 2365 presenting for the study, 687 provided consent and were screened, most were excluded due to normal ABI.
**Nitric Oxide Synthase Inducers**			
Loffredo (2014)	Patients with documented stable PAD (Fontaine Stage 2).	Kidney or liver insufficiency, acute cerebrovascular disease, acute myocardial infarction, current smokers, or taking antioxidants.	Of 32 assessed, 5 were smokers, 3 refused participation and 4 had serum creatinine outside the range.
McDermott (2017)	Patients aged at least 65 years with documented PAD (ABI <0.9, medical record-documented lower extremity revascularization, or noninvasive vascular laboratory test results consistent with PAD).	Below-knee or above- knee amputation, confined to a wheelchair, used a walking aid, had a walking impairment for a reason other than PAD, considerable visual or hearing impairment, required dialysis, lung disease requiring oxygen, substantial liver disease, had a major cardiovascular event/major surgery/endovascular revascularization in last 3 months, Mini-Mental State Examination score < 23, planned revascularization or major surgery in the next 6 months, already participating in another clinical trial, treated for cancer in the past 2 years (unless prognosis was excellent), currently taking or allergic to resveratrol, baseline 6MWT of < 152.4m or > 487.7m, or did not take at least 80% of daily placebo pills during a 2 week study run-in.	Of 125 assessed, 59 were excluded (28 for not meeting inclusion criteria, 11 for not attending the baseline or losing interest, 8 for having and extreme baseline 6MWT, 7 for having an eGFR < 30, 2 for having an MMSE score < 23 or disabling psychiatric disease, 1 for having below- or above-knee amputation, 1 for failing to complete run-in, and 1 for using a walker).
McDermott (2020)	Patients aged at least 60 years with documented PAD (ABI <0.9 or angiographic evidence).	Major leg amputation, critical limb ischaemia, confined to wheelchair, use of a walking aid, non-vascular walking impairment, significant visual or hearing impairment, dialysis, requiring oxygen, recent revascularization, major surgery or major cardiovascular event, six-minute walk test of less than 152m or more than 488m, mini-mental status examination score less than 23, or unwilling to give up major dietary sources of chocolate.	Of 118 screened, 74 were excluded (58 for not meeting eligibility criteria, 10 refusing participation, and 6 for other reasons).
Tenore (2019)	Patients aged 35-75 years with documented PAD (Fontaine Stage 2) with IC pain for at least 3 years, from a single medical center.	Current smoker, obese (BMI >30kg/m^2), severe kidney or liver disease, taking medications or supplements containing apple polyphenols, very physically active (>10 hours per week), actual or intended pregnancy, breastfeeding, allergy to birch pollen, recent use of vitamin/minerals or antioxidant supplements, or recent blood donation.	Of 253 screened, 73 did not meet inclusion/exclusion criteria.
**Antioxidsants**			
Brevetti (1988)	Patients with documented PAD (Fontaine Stage 2) diagnosed at least 1 year prior.	Heart failure, coronary artery disease, and severe hypertension.	Of 56 screened, 36 were excluded.
Brevetti (1995)	Patients aged at least 40 years with documented symptomatic PAD (ABI <0.8) diagnosed at least 1 year prior, with maximum walking capacity between 30 and 400m.	Any condition that limited exercise capacity, any medication apart from oral anti-diabetic drugs and diuretics, severe venous insufficiency, sympathectomy or angioplasty in the last 6 months, and peripheral neuropathy.	NR
Brevetti (1999)	Patients with documented symptomatic PAD (ABI <0.8) diagnosed at least 1 year prior that decreased with exercise by at least 20%, with maximum walking capacity between 50 and 400m.	Reconstructive vascular surgery, recent angioplasty, peripheral neuropathy, or any other condition that limited exercise capacity. Patients in whom the highest value of MWD during the three treadmill tests in the run-in period exceeded the lowest one by more than 50%.	Of 1773 screened, 1272 were excluded.

Collins (2003)	Patients with documented PAD (ABI <0.95 at least and/or <0.85 after exercise) and a history of intermittent claudication limiting walking.	Comorbid medical conditions, already taking vitamin E, warfarin or pentoxifylline, or walking limited by a non-PAD cause.	Of 1065 screened, 73 passed inclusion criteria, with 22 of these excluded after screening.
Coto (1992)	Patients with documented PAD (Fontaine Stage 2, ABI <0.8) with exercise-induced IC for at least 1 year, and maximum walking distance between 30 and 400m.	Resting pain or trophic lesions, any medication apart from oral hypoglycemics or diuretics, recent myocardial infarction (within 4 months) or vascular surgery (within 6 months), angina, cardiac insufficiency, uncontrolled hypertension (>165/95mmHg), ulcerative lesions, or any disease having a rapid evolution.	NR
Da Silva (2015)	Male patients with documented PAD (ABI <0.9) and stable IC.	Critical limb ischemia, unable to walk on a treadmill at 2mph, exercise limited by non-PAD diseases or conditions, or use of medications to treat claudication (pentoxifylline or cilostazol) or antioxidants.	Of 13 enrolled, two withdrew before attending the first visit.
Dal Lago (1999)	Patients with documented PAD (Fontaine Stage 2, ABI <0.8) with IC for at least 1 year, and maximum walking distance of 150 to 400m.	Rapidly worsening lower-limb arteriopathy, recent myocardial infarction (in 6 months prior to recruitment), previous stroke, diabetes, hepatic or renal impairment, active peptic ulcer, thyrotoxicosis, peripheral neuropathy, debilitating chronic illness, arteritis, venous insufficiency of lower limbs, recent peripheral revascularization in the last 6 months, recent use of propionyl-L-carnitine in the last 30 days, or use of vasodilators (except for ACE-inhibitors and diuretics).	NR
Deckert (1997)	Patients aged at least 40 years with documented PAD (ABI <0.8) and maximum walking distance of 30-400m.	Severe venous insufficiency, peripheral neuropathy, poor exercise tolerance, or recent vascular surgery, sympathectomy, or angioplasty.	NR
Gardner (2008)	Patients aged at least 18 years with documented PAD (ABI <0.9) and able to walk on a treadmill at 2mph and 10% grade for 1-10 minutes, with an ABI drop of at least 25% within 1 minute of treadmill walk.	Pregnancy, recent major surgery or cardiovascular complication (e.g. aortic or lower-limb arterial surgery, myocardial infarction, uncontrolled hypertension), active cancer, use of pentoxifylline, carnitine, arginine, prostacyclins, dietary antioxidant supplements or other products containing gingko biloba within the last month prior to screening and during the study.	Of 655 screened, 593 were ineligible or not interested, leaving 62 to be randomized.
Goldenberg (2012)	Patients aged at least 40 years with documented PAD (ABI <0.9 or 0.9-1.0 and >20% reduction in ABI after treadmill testing or toe pressure index <0.7), exercise-induced IC pain for at least 3 months, peak walking time of 1-12 minutes, and adherence >70% with cilostazol during the run-in phase.	Critical limb ischaemia, leg amputation, history of congestive heart failure, active malignancy, anticipated survival of less than 2 years, recent transient ischemic attack or deep vein thrombosis, recent stroke, coronary or peripheral revascularization, resting blood pressure >180/100mmHg, currently taking or unwilling to washout from L-carnitine, cilostazol, or pentoxifylline, currently taking and unable to discontinue ketoconazole, itraconazole or erythromycin, anticipated changes in pregnancy or smoking statuses, current pregnancy or breastfeeding, and any blood abnormalities.	Of 398 screened, 234 failed screening and one voluntary withdrew.
Grenon (2015)	Patients aged at least 50 years with documented PAD (IC and ABI <0.9; Rutherford grade 1-3) from a single vascular clinic.	Critical limb ischaemia, allergy to seafood, renal or hepatic impairment, inflammatory disorder, concurrent severe infection, recent acute illness or major surgery, and use of immunosuppressant medications.	Of 136 screened, 56 excluded for not meeting criteria or declining entry.
Hiatt (2011)	Patients aged 40-80 years with documented PAD (ABI <0.9) and IC for at least 1 year, and peak walking time of 90 to 360 seconds.	Critical limb ischaemia, walking limited by non-PAD cause, current participation in an exercise program, recent aortic or lower extremity revascularization, recent major surgery or myocardial infarction, uncontrolled hypertension, renal or hepatic insufficiency, or recent participation in a claudication or propionyl-L-carnitine trial.	Of 128 screened, 51 failed screening and 8 failed treadmill criteria.
Kiesewetter (1993)	Patients aged 40-75 years with documented PAD (femoral or tibial with angiographically localized stenosis over 60% or occlusion of the superficial femoral artery free vascular system), pain-free walking distance of 80-300m, Doppler pressure values over peripheral arteries > 50mmHg at rest, and hematocrit values up to 47%.	Pelvic arterial occlusion, stenosis >60%, Buerger disease, non-vascular walking impairment, recent surgery, severe cerebral insufficiency, polyneuropathy, recent myocardial infarction or unstable angina, heart failure, heart defect, chronic venous insufficiency, use of anticoagulants or vasoactive drugs.	NR
Leng (1997)	Patients of any age with documented PAD (ABI <0.9) and stable intermittent claudication (shown on the Edinburgh Claudication Questionnaire) from a single vascular clinic.	Critical limb ischaemia, previous or intended artery surgery or angioplasty, unstable angina or recent myocardial infarction, severe concurrent illness including liver disorders, malignancy or epilepsy, concurrent treatment with anticoagulants, lithium, or phenothaizines, or actual or intended pregnancy.	NR
Luo (2013)	Patients aged 40-75 years with documented PAD (ABI <0.9) and stable IC for at least 3 months and max walking distance of 50-250m.	Critical limb ischaemia, non-PAD walking impairment, current participation in an exercise program, recent aortic or lower extremity revascularization, recent major surgery or myocardial infarction, uncontrolled hypertension, renal or hepatic insufficiency, recent participation in a claudication or propionyl-L-carnitine trial.	NR
Park (2020)	Patients with documented PAD (Fontaine Stage 2 or 3; ABI <0.9), IC, and stable management of their blood pressure and lipids.	Rest pain or tissue loss due to PAD, non-PAD walking limitation, or kidney disease.	Of 16 assessed, 5 were excluded.

Ramirez (2019)	Patients aged at least 50 years with documented PAD (<0.9 or toe pressures <70mmHg or at least 50% stenosis of relevant arteries) and intermittent claudication (Rutherford 1 to 3) at a single medical center.	Severe acute illness within the last 30 days (infection, surgery, critical limb ischemia), taking immunosuppressants or steroids, severe renal or hepatic disease, or nonvascular inflammatory disease.	NR
Santo (2006)	Patients with documented PAD (Fontaine Stage 2 and ABI <0.9) and type 2 diabetes, and lack of a signal in at least one of three leg arteries.	Symptomatic coronary artery disease, chronic renal insufficiency, smoking within the last 12 months, active liver infection, and active infective disease	NR
Vincent (2007)	Patients aged at least 50 years with documented PAD (ABI between 0.3 and 0.9) and claudication pain when walking.	Current smoker, liver or kidney disease, or ambulatory barriers.	Of 60 screened, 28 were excluded due to ABI outside the range.

ABI = ankle-brachial pressure index; NR = not reported.

Table S3i. Risk of Bias-2 (ROB-2) quality assessment outcomes.

**Risk of bias assessment area or question**	**Bock (2018)**	**Brevetti (1988)**	**Brevetti (1995)**	**Brevetti (1999)**	**Collins (2003)**	**Coto (1992)**	**Da Silva (2015)**	**Dal Lago (1999)**	**Deckert (1997)**	**Domingues (2021)**	**Gardner (2008)**	**Goldenberg (2012)**	**Grenon (2015)**	**Gresele (2012)**	**Hiatt (2011)**	**Kenjale (2011)**	**Kiesewetter (1993)**
1.1 Was allocation sequence random?	Y	NI	NI	NI	Y	NI	NI	N	NI	Y	Y	Y	Y	Y	NI	NI	NI
1.2 Was allocation sequence concealed until participants were assigned to intervention/ control?	Y	NI	NI	NI	Y	NI	NI	NI	NI	Y	Y	Y	Y	Y	NI	NI	NI
1.3 Did baseline differences between groups suggest a problem with randomisation?	N	NI	N	N	N	N	NI	NI	N	N	Y	N	N	N	Y	NI	NI
**1. Risk of bias judgement**	**Low**	**Some**	**Some**	**Some**	**Low**	**Some**	**Some**	**Some**	**Some**	**Low**	**Some**	**Low**	**Low**	**Low**	**High**	**Some**	**Some**
2.1 Were participants aware of their assigned intervention during the trial?	N	PN	N	N	N	N	N	N	PN	N	N	N	N	N	N	Y	N
2.2 Were carers and people delivering the interventions aware of participants' assigned intervention during the trial?	N	PN	N	N	N	N	N	N	PN	N	N	N	N	N	N	Y	N
2.3. If Y/PY/NI to 2.1 or 2.2: Were there deviations from the intended intervention that arose because of the trial context?	-	-	-	-	-	-	-	-	-	-	-	-	-	-	-	N	-
2.4 If Y/PY/NI to 2.3: Were these deviations likely to have affected the outcome?	-	-	-	-	-	-	-	-	-	-	-	-	-	-	-	-	-
2.5. If Y/PY to 2.4: Were these deviations from intended intervention balanced between groups?	-	-	-	-	-	-	-	-	-	-	-	-	-	-	-	-	-
2.6 Was an appropriate analysis used to estimate the effect of assignment to intervention?	Y	NI	N	N	N	N	N	N	N	N	Y	Y	Y	Y	Y	Y	N
2.7 If N/PN/NI to 2.6: Was there potential for a substantial impact (on the result) of the failure to analyse participants in the group to which they were randomized?	-	PN	NI	NI	PN	PN	PN	PN	NI	PN / N	-	-	-	-	-	-	PN
**2. Risk of bias judgement**	**Low**	**Some**	**High**	**High**	**Some**	**Some**	**Some**	**Some**	**High**	**Some**	**Low**	**Low**	**Low**	**Low**	**Low**	**Low**	**Some**
3.1 Were data for this outcome available for all, or nearly all, participants randomized?	Y	PY	N	N	Y	N	Y	Y	N	Y	Y	N	Y	N	Y	Y	N
3.2 If N/PN/NI to 3.1: Is there evidence that the result was not biased by missing outcome data?	-	-	N	N	-	N	-	-	NI	-	-	N	-	Y	-	-	N
3.3 If N/PN to 3.2: Could missingness in the outcome depend on its true value?	-	-	NI	NI	-	PY	-	-	PY / NI	-	-	PY / NI	-	-	-	-	PN

3.4 If Y/PY/NI to 3.3: Is it likely that missingness in the outcome depended on its true value?	-	-	PN	PN	-	PN	-	-	NI	-	-	PN	-	-	-	-	-
**3. Risk of bias judgement**	**Low**	**Low**	**Some**	**Some**	**Low**	**Some**	**Low**	**Low**	**High**	**Low**	**Low**	**Some**	**Low**	**Low**	**Low**	**Low**	**Low**
4.1 Was the method of measuring the outcome inappropriate?	N	N	N	N	N	N	N	N	PN	N	N	N	N	N	N	N	N
4.2 Could measurement or ascertainment of the outcome have differed between intervention groups?	PN	PN / N	PN	PN	PN	PN	N	PN	NI	N	N	PN	N	N	N	PN	PN
4.3 If N/PN/NI to 4.1 and 4.2: Were outcome assessors aware of the intervention received by study participants?	N	PN	PN	PN	N / PN	PN	PN	PN	PN	N	N	N	PN	PN	NI	Y	NI
4.4 If Y/PY/NI to 4.3: Could assessment of the outcome have been influenced by knowledge of intervention received?	-	-	-	-	-	-	-	-	PY	-	-	-	-	-	PN	PN	PN
4.5 If Y/PY/NI to 4.4: Is it likely that assessment of the outcome was influenced by knowledge of intervention received?	-	-	-	-	-	-	-	-	PN	-	-	-	-	-	-	-	-
**4. Risk of bias judgement**	**Low**	**Low**	**Low**	**Low**	**Low**	**Low**	**Low**	**Low**	**Some**	**Low**	**Low**	**Low**	**Low**	**Low**	**Low**	**Low**	**Low**
5.1 Were the data that produced this result analysed in accordance with a pre-specified analysis plan that was finalised before unblended outcome data were available for analysis?	Y	NI	NI	NI	NI	NI	NI	NI	NI	Y	NI	Y	Y	Y	NI	NI	NI
5.2 Is the numerical result being assessed likely to have been selected, on the basis of the results from multiple eligible outcome measurements (e.g. scales, definitions, time points) within the outcome domain?	N	N	N	N	N	N	N	N	N	N	N	N	N	N	N	N	N
5.3 Is the numerical result being assessed likely to have been selected, on the basis of the results from multiple eligible analyses of the data?	N	N	N	N	N	N	N	N	N	N	N	N	N	N	N	N	N
**5. Risk of bias judgement**	**Low**	**Some**	**Some**	**Some**	**Some**	**Some**	**Some**	**Some**	**Some**	**Low**	**Some**	**Low**	**Low**	**Low**	**Some**	**Some**	**Some**
**Overall risk of bias judgement**	**Low**	**Some**	**High**	**High**	**Some**	**Some**	**Some**	**Some**	**High**	**Some**	**Some**	**Some**	**Low**	**Low**	**High**	**Some**	**Some**

Table S3ii. Risk of Bias-2 (ROB-2) quality assessment outcomes.

**Risk of bias assessment area or question**	**Leng (1997)**	**Loffredo (2014)**	**Luo (2013)**	**Maxwell (2000)**	**McDermott (2017)**	**McDermott (2020)**	**Micker (2007)**	**Mohler (2014)**	**Oka (2005)**	**Park (2020)**	**Pekas (2021)**	**Ramirez (2019)**	**Santo (2006)**	**Tenore (2019)**	**Van der Avoort (2021)**	**Vincent (2007)**	**Wilson (2007)**	**Woessner (2018)**
1.1 Was allocation sequence random?	Y	Y	NI	NI	Y	Y	NI	NI	NI	NI	NI	Y	NI	Y	Y	Y	NI	NI
1.2 Was allocation sequence concealed until participants were assigned to intervention/ control?	Y	Y	NI	NI	NI	Y / PY	NI	NI	NI	NI	NI	Y	NI	Y	Y	PY	PY	Y
1.3 Did baseline differences between groups suggest a problem with randomisation?	N	NI	NI	Y	N	N	PN / N	N	N	NI	NI	N	N	N	N	N	N	Y
**1. Risk of bias judgement**	**Low**	**Some**	**Some**	**High**	**Low**	**Low**	**Some**	**Some**	**Some**	**Some**	**Some**	**Low**	**Some**	**Low**	**Low**	**Low**	**Low**	**Some**
2.1 Were participants aware of their assigned intervention during the trial?	N	Y	N	PN	N	N	PN	N	PN	N	N	N	N	N	PN	N	N	N

2.2 Were carers and people delivering the interventions aware of participants' assigned intervention during the trial?	N	N	N	N	N	N	PN	N	PN	N	N	N	N	N	N	N	N	N
2.3. If Y/PY/NI to 2.1 or 2.2: Were there deviations from the intended intervention that arose because of the trial context?	-	N	-	-	-	-	-	-	-	-	-	-	-	-	-	-	-	-
2.4 If Y/PY/NI to 2.3: Were these deviations likely to have affected the outcome?	-	-	-	-	-	-	-	-	-	-	-	-	-	-	-	-	-	-
2.5. If Y/PY to 2.4: Were these deviations from intended intervention balanced between groups?	-	-	-	-	-	-	-	-	-	-	-	-	-	-	-	-	-	-
2.6 Was an appropriate analysis used to estimate the effect of assignment to intervention?	Y	Y	Y / PY	N	Y	Y	Y	Y / PY	N	Y	Y	Y	Y	Y	Y	N	Y	N
2.7 If N/PN/NI to 2.6: Was there potential for a substantial impact (on the result) of the failure to analyse participants in the group to which they were randomized?	-	-	-	N	-	-	-	-	PN	-	-	-	-	-	-	PN	-	N
**2. Risk of bias judgement**	**Low**	**Low**	**Low**	**Some**	**Low**	**Low**	**Low**	**Low**	**Some**	**Low**	**Low**	**Low**	**Low**	**Low**	**Low**	**Some**	**Low**	**Some**
3.1 Were data for this outcome available for all, or nearly all, participants randomized?	N	Y	Y	Y	Y	Y	Y	N	Y	Y	Y	Y	Y	Y	Y	Y	Y	N
3.2 If N/PN/NI to 3.1: Is there evidence that the result was not biased by missing outcome data?	Y	-	-	-	-	-	-	Y	-	-	-	-	-	-	-	-	-	N
3.3 If N/PN to 3.2: Could missingness in the outcome depend on its true value?	-	-	-	-	-	-	-	-	-	-	-	-	-	-	-	-	-	N
3.4 If Y/PY/NI to 3.3: Is it likely that missingness in the outcome depended on its true value?	-	-	-	-	-	-	-	-	-	-	-	-	-	-	-	-	-	-
**3. Risk of bias judgement**	**Low**	**Low**	**Low**	**Low**	**Low**	**Low**	**Low**	**Low**	**Low**	**Low**	**Low**	**Low**	**Low**	**Low**	**Low**	**Low**	**Low**	**Low**
4.1 Was the method of measuring the outcome inappropriate?	N	N	N	N	N	N	N / PN	N	N	N	N	N / PN	PN	N	N	N	N	N
4.2 Could measurement or ascertainment of the outcome have differed between intervention groups?	N	N	N	N	N	N	N / PN	N	PN	N	N	N / PN	PN	N	N	N	PN	N
4.3 If N/PN/NI to 4.1 and 4.2: Were outcome assessors aware of the intervention received by study participants?	N	N	PN	PN	N	N	PN	PN	PN	N	N	N	N	N	PN	N	PN	N
4.4 If Y/PY/NI to 4.3: Could assessment of the outcome have been	-	-	-	-	-	-	-	-	-	-	-	-	-	-	-	-	-	-

influenced by knowledge of intervention received?																		
4.5 If Y/PY/NI to 4.4: Is it likely that assessment of the outcome was influenced by knowledge of intervention received?	-	-	-	-	-	-	-	-	-	-	-	-	-	-	-	-	-	-
**4. Risk of bias judgement**	**Low**	**Low**	**Low**	**Low**	**Low**	**Low**	**Low**	**Low**	**Low**	**Low**	**Low**	**Low**	**Low**	**Low**	**Low**	**Low**	**Low**	**Low**
5.1 Were the data that produced this result analysed in accordance with a pre-specified analysis plan that was finalised before unblended outcome data were available for analysis?	PY	Y	NI	NI	PY	Y	NI	NI	NI	Y	Y	Y	NI	Y	PY	NI	Y	Y
5.2 Is the numerical result being assessed likely to have been selected, on the basis of the results from multiple eligible outcome measurements (e.g. scales, definitions, time points) within the outcome domain?	N	N	N	N	N	N	N	N	N	N	N	N	N	N	N	N	N	N
5.3 Is the numerical result being assessed likely to have been selected, on the basis of the results from multiple eligible analyses of the data?	N	N	N	N	N	N	N	N	N	N	N	N	N	N	N	N	N	N
**5. Risk of bias judgement**	**Low**	**Low**	**Some**	**Some**	**Low**	**Low**	**Some**	**Some**	**Some**	**Low**	**Low**	**Low**	**Some**	**Low**	**Low**	**Some**	**Low**	**Low**
**Overall risk of bias judgement**	**Low**	**Some**	**Some**	**Some**	**Low**	**Low**	**Some**	**Some**	**Some**	**Some**	**Some**	**Low**	**Some**	**Low**	**Low**	**Some**	**Low**	**Some**

Table S4. Comparison of primary and secondary outcome data between the intervention and control groups for each study.

**Outcomes**	**Study**	**Participants in intervention group**	**Participants in control group**	**Intervention group at baseline**	**Control group at baseline**	**Intervention group at end of study**	**Control group at end of study**	**Change from baseline (interventional group)**	**Change from baseline (control group)**
Initial claudication distance (m)	Brevetti (1988)	20		158.2 (59.8)		306.5 (121.8)	174.7 (63.1)	NR	NR
	Brevetti (1995)	99	115	125.6 (59.7)	125.1 (64.3)	222.6 (179.1)	191.6 (150.1)	91.1 (16%)	58.5 (8%)
	Brevetti (1999)	86<br>76	87<br>79	104 (37.1)<br>202 (69.7)	105 (37.3)<br>196 (62.2)	193 (157.7)<br>367 (270.3)	156 (93.3)<br>381 (293.3)	NR	NR
	Coto (1992)	142	140	104.5 (38)	99.4 (41)	153.3 (55.6)	117.3 (48.8)	NR	NR
	Da Silva (2015)	10		NR	NR	NR	NR	NR	NR
	Dal Lago (1999)	9	10	109.6 (33.9)	113.2 (30.8)	163.3 (53.3)	112.2 (38.8)	NR	NR
	Domingues (2021)	14	15	143 (65)	143 (84)	193 (110)	145 (90)	NR	NR
	Gresele (2012)	221	221	131.5 (77.3)	131.5 (78.0)	203.0 (162.6)	193.2 (138.8)	84.3 (100.2)	84.5 (88.5)
	Kiesewetter (1993)	32	32	161.0 (65.1)	172.0 (60.0)	207.1 (85.0)	203.1 (72.8)	46	31
	Leng (1997)	26	34	45.4 (42.9)	48.5 (43.8)	NR	NR	NR	NR
	Maxwell (2000)	15<br>12	13	206 (269.4)<br>159 (117.8)	116 (61.3)	221 (258.2)<br>260 (232.1)	135 (79.3)	14 (67.3)<br>100 (155.9)	19 (39.7)
	Micker (2007)	24	24	80.4 (44.2)	57.1 (27.3)	438.8 (532.6)	74.2 (42.3)	NR	NR

	Oka (2005)	18<br>17<br>19	18	150.0 (96.9)<br>113.2 (73.7)<br>123.4 (61.2)	121.3 (61.5)	191.6 (114.4)<br>179.2 (115.6)<br>198.0 (181.6)	152.6 (101.8)	NR	NR
	Ramirez (2019)	11	13	119 (74)	219 (125)	225 (168)*	213 (195)*	106 (92)	-6 (99)
	Santo (2006)	37	37	366.4 (8)	337.3 (90.9)	519.8 (9)	331.8 (86.4)	NR	NR
	Vincent (2007)	16	12	169.6 (145.9)	150.2 (150.5)	233.4 (159.1)	201.9 (162.1)	22.1	27.0
	Wilson (2007)	58	61	91 (7.1)	105 (7.0)	110 (11)	146 (12)	19.0 (9.2)	41 (8.9)
Maximum walking distance (m)	Brevetti (1995)	99	115	214.6 (109.4)	207.8 (107.2)	354.1 (218.9)	298.1 (193.0)	+72.7 (+/- 9%)	+45.6 (+/-6%)
	Brevetti (1999)	86<br>76	87<br>79	169 (46.4)<br>332 (43.6)	174 (46.6)<br>323 (35.6)	342 (278.2)<br>574 (322.6)	269 (177.2)<br>612 (328.9)	NR	NR
	Coto (1992)	142	140	177.5 (51.6)	173.7 (57)	270.6 (78.5)	208.8 (68.4)	93	35
	Da Silva (2015)	10		NR	NR	NR	NR	NR	NR
	Dal Lago (1999)	9	10	217.5 (60.4)	219.6 (80.7)	360.8 (228.9)	237.3 (108.1)	NR	NR
	Gresele (2012)	221	221	210 (107)	217 (114)	327 (244)	344 (254)	117 (137)	126 (140)
	Loffredo (2014)	20		110.7 (64.5)	115.8 (71.9)	122.2 (61.5)	109.1 (65.1)	NR	NR
	Maxwell (2000)	15<br>12	13	226 (280.6)<br>197 (155.9)	267 (432.7)	339 (310.6)<br>327 (239.0)	236 (147.8)	32 (86.1)<br>77 (107.4)	8 (43.3)
	Micker (2007)	24	24	159.0 (87.3)	109.0 (50.9)	687.1 (769.7)	137.7 (86.5)	NR	NR
	Oka (2005)	18<br>17<br>19	18	297.1 (139.8)<br>306.3 (151.9)<br>299.7 (131.3)	299.0 (115.6)	398.5 (208.8)<br>371.4 (188.9)<br>372.1 (222.1)	352.9 (152.0)	NR	NR
	Park (2020)	11		438.5 (218.2)	458.7 (209.3)	487.8 (218.4)	435.4 (218.1)	49.2 (87.3)	-23.4 (40.7)
	Pekas (2021)	11		367.7 (244.5)	365.9 (245.7)	460.5 (279.3)	361.6 (263.1)	92.7 (110.5)	-4.3 (50.8)
	Wilson (2007)	58	61	280 (16)	310 (19)	314 (25)	392 (28)	36 (17)	78 (16)
6-minute walking distance (m)	Bock (2018)	13	8	387 (90)	423 (56)	425 (82)	427 (66)	NR	NR
	Domingues (2021)	14	15	344 (82)	371 (81)	369 (115)	382 (99)	NR	NR
	McDermott (2017)	20<br>23	21	369.7 (64.9)<br>357.4 (57.7)	353.8 (70.4)	374.4 (63.9)<br>344.6 (55.3)	341.5 (85.2)	4.6 (36.8)<br>-12.8 (36.8)	-12.3 (36.9)
	McDermott (2020)	22	21	338.9 (74.7)	330.7 (86.4)	346.6 (74.2)	336.4 (88.3)	17.2 (40.1)	-4.3 (38.3)
	Mohler (2014)	19<br>18	18	NR<br>NR	NR	NR<br>NR	NR	NR	NR
	Vincent (2007)	16	12	365.1 (124.0)	375.9 (91.2)	380.7 (126.6)	395.8 (83.8)	18.9	21.3
	Woessner (2018)	11	13	318.5 (63.1)	354.2 (118.0)	371.8 (67.5)	378.8 (106.7)	53.3 (19.6)	24.6 (12.1)
Initial claudication time (s)	Gardner (2008)	31	31	67.0 (29.7)	66.7 (42.1)	NR	NR	21 (43)	15 (31)
	Goldenberg (2012)	74	71	NR	NR	NR	NR	1.07 (0.67)	0.90 (0.74)
	Hiatt (2011)	32	30	69 (35)	60 (24)	NR	NR	174 (183)	100 (100)
	Kenjale (2011)	8		NR	NR	215 (99)	183 (84)	32 (18%)	NR
	Luo (2013)	120	119	122 (67)	127 (65)	166 (106)	149 (80)	44 (77)	22 (54)
	McDermott (2017)	16<br>18	19	264 (138)<br>318 (264)	258 (168)	300 (132)<br>312 (174)	276 (180)	36 (146.9)<br>-6 (149.4)	18 (153.5)
	McDermott (2020)	18	19	365 (257)	314 (203)	373 (229)	326 (192)	-4 (138.5)	23 (137.9)
	Park (2020)	11		202.3 (154.5)	205.4 (126.1)	246.5 (152.6)	169.4 (142.5)	44.2 (51.3)	-36.0 (34.0)
	Pekas (2021)	11		220.6 (121.3)	213.2 (106.7)	241.1 (102.7)	180.6 (121.7)	20.6 (157.3)	-32.7 (34.3)
	Ramirez (2019)	11	13	113 (86)	165 (99)	NR	NR	94 (88)	7 (89)
	Van der Avoort (2021)	18		NR	NR	413 (187)<br>392 (154)	403 (176)	NR	NR

	Vincent (2007)	16	12	123.2 (106.1)	129.8 (113.1)	211.5 (132.2)	157.6 (145.1)	34.4	19.4
	Woessner (2018)	11	13	252.6 (131.3)	302.8 (285.9)	432.2 (218.9)	362.0 (235.2)	180.3 (46.6)	59.2 (57.3)
Maximum walking time (s)	Collins (2003)	13	10	705 (951)	612 (378)	664 (548)	623 (531)	NR	NR
	Gardner (2008)	31	31	226.6 (126.8)	205.6 (126.0)	NR	NR	91 (242)	20 (80)
	Goldenberg (2012)	74	71	328.2 (102)	315.6 (100.2)	417.6 (110.4)	359.4 (115.8)	76.2 (87.6)	68.6 (83.5)
	Hiatt (2011)	32	30	354 (143)	339 (150)	NR	NR	266 (243)	218 (367)
	Kenjale (2011)	8		NR	NR	533 (233)	467 (223)	65 (17%)	NR
	Loffredo (2014)	20		124.8 (60.8)	124.5 (60.1)	142.2 (62.0)	125.4 (64.1)	NR	NR
	Luo (2013)	120	119	192 (98)	213 (149)	286 (177)	226 (157)	94 (113)	14 (80)
	McDermott (2017)	20<br>22	20	570 (282)<br>606 (312)	522 (270)	534 (234)<br>570 (282)	546 (288)	30 (130.1)<br>36 (136.4)	24 (136.9)
	McDermott (2020)	18	20	532 (292)	586 (444)	548 (266)	586 (514)	-2 (147.2)	17 (144.9)
	Park (2020)	11		442.7 (166.2)	457.2 (112.8)	516.5 (149.0)	435.7 (124.5)	73.8 (134.0)	-21.5 (30.4)
	Pekas (2021)	11		399.9 (151.6)	418.9 (146.9)	456.2 (144.9)	401.7 (146.9)	56.3 (76.1)	29.4 (126.2)
	Van der Avoort (2021)	18		NR	NR	745 (220)<br>746 (176)	696 (222)	NR	NR
	Woessner (2018)	11	13	537.5 (188.0)	657.5 (418.5)	807.4 (182.8)	896.2 (378.1)	269.9 (195.3)	238.7 (207.0)
Quality of life (SF-36): reported as physical component / mental component	Collins (2003)	13	10	43.1 (15.6) / 76.9 (16.1)	46.7 (21.5) / 79.3 (18.1)	45.8 (16.8) / 80.0 (14.2)	50.0 (19.7) / 74.4 (15.8)	NR	NR
	Gardner (2008)	30	27	38.0 (5.1) / 47.0 (13.1)	39.2 (5.7) / 49.7 (12.1)	38.3 (4.7) / 50.3 (10.6)	38.6 (5.3) / 49.8 (12.5)	NR	NR
	Goldenberg (2012)[#]	69	68	49.1 (16.6)	48.8 (19.8)	56.2 (21.5)	52.4 (18.7)	6.7 (16.4)	3.7 (17.6)
	Gresele (2012)	221	221	52.3 (17.8) / 66.1 (18.0)	58.3 (15.1) / 70.4 (15.1)	55.4 (17.8) / 65.9 (18.4)	60 (15) 70.2 (17.3)	3.1 (14.0) / -0.22 (24.4)	1.7 (14.7) / -0.27 (22.4)
	Hiatt (2011)	32	30	35.8 (8.6) / 49.5 (16.7)	32.6 (7.4) / 41.2 (15.7)	40 (10.4)	43.3 (8.1)	4.2 (7.2) / 12.7 (11.5)	3.3 (7.6) / 8.8 (23.2)
	Maxwell (2000)	14<br>12	13	62 (22.4) / 73 (7.5)<br>59 (31.2) / 60 (17.3)	58 (25.2) / 71 (10.8)	65 (22.4) / 75 (3.7)<br>62 (27.7) / 66 (17.3)	66 (28.8) / 68 (10.8)	NR	NR
	Mohler (2014)	19<br>18	18	NR<br>NR	NR	NR<br>NR	NR	NR	NR
	Wilson (2007)	66	67	NR	NR	NR	NR	NR	NR
Quality of life (WIQ)	Collins (2003): walking distance / speed	13	10	0.30 (0.21) / 0.28 (0.20)	0.38 (0.30) / 0.28 (0.20)	0.26 (0.28) / 0.27 (0.17)	0.36 (0.34) / 0.33 (0.32)	NR	NR
	Gardner (2008): walking distance / speed / stairs	30	26	0.45 (0.25) / 0.44 (0.21) / 0.51 (0.25)	0.53 (0.32) / 0.42 (0.22) / 0.51 (0.27)	0.52 (0.32) / 0.50 (0.23) / 0.52 (0.29)	0.54 (0.33) / 0.50 (0.26) / 0.49 (0.31)	NR	NR
	Goldenberg (2012): walking distance	68	68	21.5 (20.9)	26.9 (24.2)	35.4 (29.4)	33.8 (27.1)	13.2 (22.0)	6.6 (24.3)
	Grenon (2015): walking distance / speed / stairs	40	40	25 (30) / 22 (23) / 32 (31)	32 (27) / 30 (27) / 34 (26)	27 (36.3) / 26 (27.8) / 31 (37.5)*	29 (29.4) / 29 (29.4) / 54 (28.3)*	2 (21) / 4 (17) / -1 (20)	-3 (10) / -1 (18) / 0.09 (18)
	Hiatt (2011): walking distance / speed	32	30	21.6 (20.0) / 30.6 (22.2)	17.7 (18.6) / 22.8 (15.8)	49.5 (24.2) / 42.8 (26.9)*	37.9 (20.3) / 29.4 (17.2)*	27.9 (20.9) / 12.2 (19.6)	20.9 (28.2) / 6.6 (17.8)
	Mohler (2014)	19<br>18	18	NR<br>NR	NR	NR<br>NR	NR	NR	NR
	Oka (2005): walking speed only	18<br>17<br>19	18	24.7 (14.2)<br>24.2 (17.8)<br>29.2 (17.1)	33.6 (27.5)	29.6 (27.6)<br>32.1 (22.2)<br>39.3 (25.0)	42.1 (27.6)	NR	NR

	Ramirez (2019): walking distance / speed / stairs	11	13	42 (32) / 38 (18) / 40 (34)	42 (35) / 41 (30) / 49 (30)	53 (38.7) / 38.3 (21.8) / 44.2 (41.1)*	43 (38.2) / 42.4 (32.7) / 52.1 (32.7)*	11 (14) / 0.3 (23.5) / 4.2 (17)	1 (26) / 1.2 (31.6) / 3.1 (18)
Quality of life (other assessment)	Brevetti (1995)	85	102	0.59 (0.12)	0.63 (0.12)	0.64 (0.12)	0.64 (0.13)	NR	NR
	Brevetti (1999)	53	61	NR	NR	NR	NR	-2.3 (5.1)	-0.4 (3.9)
ABI	Bock (2018)	13	8	0.76 (0.21)	0.81 (0.14)	0.86 (0.21)	0.85 (0.15)	NR	NR
	Brevetti (1988)	20		0.65 (0.15)		0.64 (0.14)	0.61 (0.11)	NR	NR
	Collins (2003)	13	10	0.63 (0.15)	0.77 (0.10)	0.63 (0.12)	0.82 (0.17)	NR	NR
	Dal Lago (1999)	9	10	0.71 (0.08)	0.69 (0.07)	0.66 (0.09)	0.72 (0.15)	NR	NR
	Grenon (2015)	40	40	0.73 (0.12)	0.71 (0.14)	0.69 (0.12)*	0.68 (0.18)*	-0.02 (0.1)	-0.03 (0.1)
	Gresele (2012)	221	221	0.66 (0.15)	0.64 (0.15)	0.67 (0.16)	0.66 (0.14)	0.00 (0.08)	0.02 (0.10)
	Leng (1997)	33	42	0.67 (0.22)	0.68 (0.16)	NR	NR	NR	NR
	Luo (2013)	120	119	0.66 (0.21)	0.68 (0.17)	0.73 (0.22)	0.71 (0.18)	0.05 (0.12)	0.02 (0.12)
	Ramirez (2019)	11	13	0.60 (0.09)	0.66 (0.09)	0.66 (0.09)*	0.71 (0.12)*	0.06 (0.18)	0.05 (0.11)
	Santo (2006)	37	37	0.78 (0.04)	0.73 (0.06)	0.88 (0.03)	0.72 (0.06)	NR	NR
	Tenore (2019)	90	90	NR	NR	NR	NR	NR	NR
	Wilson (2007)	52	56	0.60 (0.02)	0.56 (0.02)	0.71 (0.03)	0.67 (0.03)	0.11 (0.02)	0.12 (0.02)
	Woessner (2018)	11	13	0.61 (0.16)	0.70 (0.19)	0.77 (0.20)	0.76 (0.22)	0.16 (0.11)	0.06 (0.11)
Adverse events	Bock (2018)	13	8	NA	NA	0	0	NA	NA
	Brevetti (1995)	99	115	NA	NA	11	3	NA	NA
	Brevetti (1999)	239	246	NA	NA	38	98	NA	NA
	Coto (1992)	142	140	NA	NA	3	5	NA	NA
	Gardner (2008)	31	31	NA	NA	2	2	NA	NA
	Goldenberg (2012)	80	83	NA	NA	35	39	NA	NA
	Gresele (2012)	221	221	NA	NA	90 (40.7%)	69 (31.2%)	NA	NA
	Hiatt (2011)	34	35	NA	NA	24 (70.6%)	24 (68.6%)	NA	NA
	Luo (2013)	120	119	NA	NA	36 (30.0%)	30 (25.2%)	NA	NA
	Maxwell (2000)	15<br>12	13	NA	NA	0<br>2 (16.7%)	1 (7.7%)	NA	NA
	Mohler (2014)	19<br>18	18	NA	NA	12 (63.2%)<br>14 (77.8%)	9 (50.0%)	NA	NA
	Van der Avoort (2021)	18		NA	NA	0<br>0	0	NA	NA
	Vincent (2007)	16	12	NA	NA	7 (43.8%)	0	NA	NA
	Wilson (2007)	66	67	NA	NA	55 (83.3%)	44 (65.7%)	NA	NA
	Woessner (2018)	11	13	NA	NA	1	0	NA	NA
Serious adverse events	Bock (2018)	13	8	NA	NA	0	0	NA	NA
	Collins (2003)	13	12	NA	NA	1	2	NA	NA
	Coto (1992)	142	140	NA	NA	0	1	NA	NA
	Brevetti (1999)	239	246	NA	NA	27 (11.3%)	30 (12.2%)	NA	NA
	Gresele (2012)	221	221	NA	NA	6 (2.7%)	4 (1.8%)	NA	NA
	Goldenberg (2012)	80	83	NA	NA	12	19	NA	NA
	Hiatt (2011)	34	35	NA	NA	4 (11.8%)	6 (17.1%)	NA	NA
	Kiesewetter (1993)	32	32	NA	NA	0	0	NA	NA
	Leng (1997)	55	65	NA	NA	12 (21.8%)	26 (40.0%)	NA	NA
	Luo (2013)	120	119	NA	NA	2 (1.7%)	4 (3.4%)	NA	NA

	Maxwell (2000)	15<br>12	13	NA	NA	0<br>0	0	NA	NA
	McDermott (2020)	23	21	NA	NA	5 (21.7%)	2 (9.5%)	NA	NA
	Mohler (2014)	19<br>18	18	NA	NA	0<br>0	2	NA	NA
	Van der Avoort (2021)	18		NA	NA	0<br>0	0	NA	NA
	Wilson (2007)	66	67	NA	NA	9 (13.7%)	9 (13.4%)	NA	NA
	Woessner (2018)	12	14	NA	NA	0	0	NA	NA
Mortality	Bock (2018)	13	8	NA	NA	0	0	NA	NA
	Brevetti (1999)	239	246	NA	NA	5 (2.1%)	5 (2.0%)	NA	NA
	Gresele (2012)	221	221	NA	NA	0	1 (0.5%)	NA	NA
	Goldenberg (2012)	80	83	NA	NA	1 (1.25%)	0	NA	NA
	Leng (1997)	55	65	NA	NA	3 (5.5%)	3 (4.6%)	NA	NA
	McDermott (2020)	23	21	NA	NA	1	0	NA	NA
	Ramirez (2019)	11	13	NA	NA	0	0	NA	NA
	Santo (2006)	37	37	NA	NA	0	0	NA	NA
	Tenore (2019)	90	90	NA	NA	0	0	NA	NA
	Van der Avoort (2021)	18		NA	NA	0<br>0	0	NA	NA
	Vincent (2007)	16	12	NA	NA	0	0	NA	NA
	Wilson (2007)	66	67	NA	NA	1 (1.5%)	1 (1.5%)	NA	NA
	Woessner (2018)	11	13	NA	NA	0	0	NA	NA
Requirement of lower extremity revascularization or amputation	Brevetti (1999)	239	246	NA	NA	2	0	NA	NA
	Leng (1997)	55	65	NA	NA	2 (3.6%)	3 (4.6%)	NA	NA
	McDermott (2020)	23	21	NA	NA	2 (8.7%)	0	NA	NA
	Park (2020)	11		NA	NA	0	0	NA	NA
	Pekas (2021)	11		NA	NA	0	0	NA	NA
	Van der Avoort (2021)	18		NA	NA	0<br>0	0	NA	NA
Initial claudication distance (m) – converted from initial claudication time (s)	Gardner (2008) – treadmill 2 mph at a 10% grade	31	31	59.9 (26.6)	59.6 (37.6)	78.7 (60.3)*	73 (58.7)*	18.8 (38.4)	13.4 (27.7)
	Goldenberg (2012) - treadmill 2mph, 2% grade increase q2min	74	71	NR	NR	NR	NR	156.4 (104.8)	131.9 (112.4)
	Hiatt (2011) – treadmill 2mph, 12% grade	32	30	61.7 (31.3)	53.6 (21.5)	217.3 (71)*	143 (33.5)*	155.6 (163.6)	89.4 (89.4)
	Kenjale (2011) – treadmill 2mph, 2% grade increase q2min	8		NR	NR	192.2 (88.5)	163.6 (75.1)	28.6 (18%)	NR

	Luo (2013) – treadmill 2mph, 12% slope	120	119	109.1 (59.9)	113.5 (58.1)	148.4 (94.8)	133.2 (71.5)	39.3 (68.8)	19.7 (48.3)
	McDermott (2020) - treadmill 2mph, 2% grade increase q2min	18	19	326.3 (229.8)	280.7 (181.5)	333.5 (204.7)	291.5 (171.7)	-3.6 (123.8)	20.6 (123.3)
	Park (2020) – 2mph, 2% grade increase q2min	11		180.9 (138.1)	183.6 (112.7)	220.4 (136.4)	151.5 (127.4)	39.5 (45.9)	-32.2 (30.4)
	Pekas (2021) – 2mph, 2% grade increase q2min	11		197.2 (108.5)	190.6 (95.4)	215.6 (91.8)	161.5 (108.9)	18.4 (140.6)	-29.2 (30.7)
	Van der Avoort (2021) – 3.2kph, 2% grade increase q2m	18		NR	NR	367.1 (166.2)<br>348.4 (136.9)	358.2 (156.4)	NR	NR
Maximum walking distance (m) – converted from maximum walking time (s)	Collins (2003) – 2.9kph at 12% grade	13	10	567.9 (766.1)	493 (304.5)	534.9 (441.4)	501.9 (427.8)	NR	NR
	Gardner (2008) – treadmill 2 mph at a 10% grade	31	31	202.6 (113.4)	183.8 (112.7)	NR	NR	81.4 (216.4)	17.9 (71.5)
	Goldenberg (2012) – 2mph, 2% grade increase q2min	74	71	293.4 (91.2)	282.2 (89.6)	373.4 (98.7)	321.3 (103.5)	68.1 (78.3)	61.3 (74.7)
	Hiatt (2011) – treadmill 2mph, 12% grade	32	30	316.5 (127.9)	303.1 (134.1)	NR	NR	237.8 (217.3)	194.9 (328.1)
	Kenjale (2011) - treadmill 2mph, 2% grade increase q2min	8		NR	NR	476.5 (208.3)	417.5 (199.4)	65 (17%)	NR
	Luo (2013) - treadmill 2mph, 12% slope	120	119	171.7 (87.6)	190.4 (133.2)	255.7 (158.3)	202.1 (140.4)	84 (101)	12.5 (71.5)
	McDermott (2020) - treadmill 2mph, 2% grade increase q2min	18	20	475.7 (261.1)	523.9 (397)	490 (237.8)	523.9 (459.6)	-1.8 (131.6)	15.2 (129.6)
	Van der Avoort (2021) – 3.2kph, 2% grade increase q2m	18		NR	NR	662.2 (195.6)<br>663.1 (156.4)	618.7 (197.3)	NR	NR

Only physical functioning score reported. *Estimated from baseline and change data using formulas detailed in Supplementary Text 2.

Table S5. Impact of dietary sources of NO upregulation on maximum walking distance - leave-one-out (LOO) sensitivity analyses.

Study excluded	Effect size estimate SMD [95% CI]	Heterogeneity	Test for overall effect (Z)
None	0.13 [-0.17, 0.43]	Heterogeneity: Tau² = 0.39; Chi² = 190.04, df = 22 (P < 0.00001); I² = 88%	Test for overall effect: Z = 0.86 (P = 0.39)

Brevetti 1995	0.12 [-0.20, 0.45]	Heterogeneity: Tau² = 0.45; Chi² = 189.58, df = 21 (P < 0.00001); I² = 89%	Test for overall effect: Z = 0.75 (P = 0.45)
Brevetti 1999	0.14 [-0.21, 0.48]	Heterogeneity: Tau² = 0.48; Chi² = 185.76, df = 20 (P < 0.00001); I² = 89%	Test for overall effect: Z = 0.78 (P = 0.43)
Collins 2003	0.13 [-0.18, 0.44]	Heterogeneity: Tau² = 0.40; Chi² = 189.97, df = 21 (P < 0.00001); I² = 89%	Test for overall effect: Z = 0.85 (P = 0.39)
Coto 1992	0.09 [-0.22, 0.39]	Heterogeneity: Tau² = 0.38; Chi² = 158.21, df = 21 (P < 0.00001); I² = 87%	Test for overall effect: Z = 0.56 (P = 0.57)
Dal Lago 1999	0.11 [-0.20, 0.42]	Heterogeneity: Tau² = 0.40; Chi² = 188.97, df = 21 (P < 0.00001); I² = 89%	Test for overall effect: Z = 0.70 (P = 0.48)
Goldenberg 2012	0.11 [-0.21, 0.43]	Heterogeneity: Tau² = 0.42; Chi² = 185.96, df = 21 (P < 0.00001); I² = 89%	Test for overall effect: Z = 0.68 (P = 0.50)
Gresele 2012	0.15 [-0.19, 0.48]	Heterogeneity: Tau² = 0.48; Chi² = 180.94, df = 21 (P < 0.00001); I² = 88%	Test for overall effect: Z = 0.85 (P = 0.39)
Kenjale 2011	0.13 [-0.18, 0.44]	Heterogeneity: Tau² = 0.40; Chi² = 190.03, df = 21 (P < 0.00001); I² = 89%	Test for overall effect: Z = 0.82 (P = 0.41)
Loffredo 2014	0.13 [-0.18, 0.44]	Heterogeneity: Tau² = 0.40; Chi² = 190.04, df = 21 (P < 0.00001); I² = 89%	Test for overall effect: Z = 0.82 (P = 0.41)
Luo 2013	0.12 [-0.21, 0.45]	Heterogeneity: Tau² = 0.45; Chi² = 188.03, df = 21 (P < 0.00001); I² = 89%	Test for overall effect: Z = 0.72 (P = 0.47)
Maxwell 2000	0.11 [-0.21, 0.43]	Heterogeneity: Tau² = 0.40; Chi² = 189.68, df = 20 (P < 0.00001); I² = 89%	Test for overall effect: Z = 0.69 (P = 0.49)
McDermott 2020	0.14 [-0.17, 0.46]	Heterogeneity: Tau² = 0.40; Chi² = 189.32, df = 21 (P < 0.00001); I² = 89%	Test for overall effect: Z = 0.90 (P = 0.37)
Micker 2007	0.09 [-0.22, 0.40]	Heterogeneity: Tau² = 0.39; Chi² = 183.06, df = 21 (P < 0.00001); I² = 89%	Test for overall effect: Z = 0.56 (P = 0.57)
Oka 2005	0.13 [-0.20, 0.46]	Heterogeneity: Tau² = 0.41; Chi² = 189.95, df = 19 (P < 0.00001); I² = 90%	Test for overall effect: Z = 0.79 (P = 0.43)
Park 2020	0.13 [-0.18, 0.44]	Heterogeneity: Tau² = 0.40; Chi² = 190.03, df = 21 (P < 0.00001); I² = 89%	Test for overall effect: Z = 0.82 (P = 0.41)
Pekas 2021	0.13 [-0.18, 0.43]	Heterogeneity: Tau² = 0.40; Chi² = 189.98, df = 21 (P < 0.00001); I² = 89%	Test for overall effect: Z = 0.80 (P = 0.42)
Van der Avoort 2021	0.13 [-0.18, 0.44]	Heterogeneity: Tau² = 0.40; Chi² = 190.03, df = 20 (P < 0.00001); I² = 89%	Test for overall effect: Z = 0.80 (P = 0.42)
Wilson 2007	0.30 [0.13, 0.47]	Heterogeneity: Tau² = 0.07; Chi² = 49.81, df = 21 (P = 0.0004); I² = 58%	Test for overall effect: Z = 3.42 (P = 0.0006)

Table S6. Impact of dietary sources of NO upregulation on initial claudication distance - leave-one-out (LOO) sensitivity analyses.

Study excluded	Effect size estimate SMD [95% CI]	Heterogeneity	Test for overall effect (Z)
None	0.34 [0.04, 0.64]	Heterogeneity: Tau² = 0.51; Chi² = 263.14, df = 27 (P < 0.00001); I² = 90%	Test for overall effect: Z = 2.23 (P = 0.03)
Brevetti 1988	0.31 [0.01, 0.62]	Heterogeneity: Tau² = 0.51; Chi² = 258.64, df = 26 (P < 0.00001); I² = 90%	Test for overall effect: Z = 2.00 (P = 0.05)
Brevetti 1995	0.35 [0.03, 0.68]	Heterogeneity: Tau² = 0.58; Chi² = 262.98, df = 26 (P < 0.00001); I² = 90%	Test for overall effect: Z = 2.13 (P = 0.03)
Brevetti 1999	0.37 [0.03, 0.71]	Heterogeneity: Tau² = 0.62; Chi² = 259.61, df = 25 (P < 0.00001); I² = 90%	Test for overall effect: Z = 2.11 (P = 0.03)

Coto 1992	0.34 [0.04, 0.64]	Heterogeneity: Tau² = 0.51; Chi² = 263.14, df = 27 (P < 0.00001); I² = 90%	Test for overall effect: Z = 2.23 (P = 0.03)
Dal Lago 1999	0.32 [0.01, 0.63]	Heterogeneity: Tau² = 0.51; Chi² = 260.43, df = 26 (P < 0.00001); I² = 90%	Test for overall effect: Z = 2.05 (P = 0.04)
Domingues 2021	0.34 [0.03, 0.65]	Heterogeneity: Tau² = 0.52; Chi² = 262.77, df = 26 (P < 0.00001); I² = 90%	Test for overall effect: Z = 2.15 (P = 0.03)
Gardner 2008	0.35 [0.04, 0.67]	Heterogeneity: Tau² = 0.53; Chi² = 262.80, df = 26 (P < 0.00001); I² = 90%	Test for overall effect: Z = 2.22 (P = 0.03)
Gresele 2012	0.36 [0.02, 0.70]	Heterogeneity: Tau² = 0.63; Chi² = 258.82, df = 26 (P < 0.00001); I² = 90%	Test for overall effect: Z = 2.09 (P = 0.04)
Hiatt 2011	0.30 [-0.00, 0.61]	Heterogeneity: Tau² = 0.49; Chi² = 248.40, df = 26 (P < 0.00001); I² = 90%	Test for overall effect: Z = 1.95 (P = 0.05)
Kenjale 2011	0.34 [0.04, 0.65]	Heterogeneity: Tau² = 0.51; Chi² = 263.13, df = 26 (P < 0.00001); I² = 90%	Test for overall effect: Z = 2.20 (P = 0.03)
Kiesewetter 1993	0.36 [0.04, 0.67]	Heterogeneity: Tau² = 0.53; Chi² = 262.54, df = 26 (P < 0.00001); I² = 90%	Test for overall effect: Z = 2.23 (P = 0.03)
Luo 2013	0.35 [0.03, 0.68]	Heterogeneity: Tau² = 0.58; Chi² = 262.90, df = 26 (P < 0.00001); I² = 90%	Test for overall effect: Z = 2.12 (P = 0.03)
Maxwell 2000	0.33 [0.02, 0.65]	Heterogeneity: Tau² = 0.52; Chi² = 262.45, df = 25 (P < 0.00001); I² = 90%	Test for overall effect: Z = 2.08 (P = 0.04)
McDermott 2020	0.35 [0.04, 0.66]	Heterogeneity: Tau² = 0.52; Chi² = 263.13, df = 26 (P < 0.00001); I² = 90%	Test for overall effect: Z = 2.20 (P = 0.03)
Micker 2007	0.32 [0.01, 0.63]	Heterogeneity: Tau² = 0.51; Chi² = 257.65, df = 26 (P < 0.00001); I² = 90%	Test for overall effect: Z = 2.02 (P = 0.04)
Oka 2005	0.35 [0.03, 0.67]	Heterogeneity: Tau² = 0.53; Chi² = 263.09, df = 24 (P < 0.00001); I² = 91%	Test for overall effect: Z = 2.13 (P = 0.03)
Park 2020	0.34 [0.03, 0.65]	Heterogeneity: Tau² = 0.51; Chi² = 262.99, df = 26 (P < 0.00001); I² = 90%	Test for overall effect: Z = 2.17 (P = 0.03)
Pekas 2021	0.34 [0.03, 0.65]	Heterogeneity: Tau² = 0.51; Chi² = 262.98, df = 26 (P < 0.00001); I² = 90%	Test for overall effect: Z = 2.17 (P = 0.03)
Ramirez 2019	0.35 [0.04, 0.66]	Heterogeneity: Tau² = 0.52; Chi² = 262.95, df = 26 (P < 0.00001); I² = 90%	Test for overall effect: Z = 2.24 (P = 0.02)
Santo 2006	0.23 [-0.04, 0.51]	Heterogeneity: Tau² = 0.38; Chi² = 197.00, df = 26 (P < 0.00001); I² = 87%	Test for overall effect: Z = 1.67 (P = 0.10)
van der Avoort 2021	0.36 [0.05, 0.67]	Heterogeneity: Tau² = 0.52; Chi² = 262.88, df = 25 (P < 0.00001); I² = 90%	Test for overall effect: Z = 2.27 (P = 0.02)
Vincent 2007	0.35 [0.04, 0.66]	Heterogeneity: Tau² = 0.52; Chi² = 263.12, df = 26 (P < 0.00001); I² = 90%	Test for overall effect: Z = 2.21 (P = 0.03)
Wilson 2007	0.48 [0.26, 0.69]	Heterogeneity: Tau² = 0.19; Chi² = 111.14, df = 26 (P < 0.00001); I² = 77%	Test for overall effect: Z = 4.35 (P < 0.0001)

Table S7. Impact of dietary sources of NO upregulation on 6-minute walking distance - leave-one-out (LOO) sensitivity analyses.

Study excluded	Effect size estimate SMD [95% CI]	Heterogeneity	Test for overall effect (Z)
None	0.02 [-0.26, 0.30]	Heterogeneity: Tau² = 0.00; Chi² = 0.78, df = 6 (P = 0.99); I² = 0%	Test for overall effect: Z = 0.13 (P = 0.89)
Bock 2018	0.02 [-0.27, 0.32]	Heterogeneity: Tau² = 0.00; Chi² = 0.77, df = 5 (P = 0.98); I² = 0%	Test for overall effect: Z = 0.16 (P = 0.87)
Domingues 2021	0.04 [-0.26, 0.34]	Heterogeneity: Tau² = 0.00; Chi² = 0.62, df = 5 (P = 0.99); I² = 0%	Test for overall effect: Z = 0.28 (P = 0.78)
McDermott 2017	-0.03 [-0.36, 0.30]	Heterogeneity: Tau² = 0.00; Chi² = 0.39, df = 4 (P = 0.98); I² = 0%	Test for overall effect: Z = 0.17 (P = 0.87)

McDermott 2020	-0.01 [-0.32, 0.30]	Heterogeneity: Tau² = 0.00; Chi² = 0.63, df = 5 (P = 0.99); I² = 0%	Test for overall effect: Z = 0.06 (P = 0.95)
Vincent 2007	0.04 [-0.26, 0.34]	Heterogeneity: Tau² = 0.00; Chi² = 0.60, df = 5 (P = 0.99); I² = 0%	Test for overall effect: Z = 0.28 (P = 0.78)
Woessner 2018	0.03 [-0.26, 0.33]	Heterogeneity: Tau² = 0.00; Chi² = 0.72, df = 5 (P = 0.98); I² = 0%	Test for overall effect: Z = 0.21 (P = 0.83)

Table S8. Impact of dietary sources of NO upregulation on quality of life (SF-36 Physical Function) - leave-one-out (LOO) sensitivity analyses.

Study excluded	Effect size estimate SMD [95% CI]	Heterogeneity	Test for overall effect (Z)
None	-0.16 [-0.32, -0.00]	Heterogeneity: Tau² = 0.00; Chi² = 6.41, df = 6 (P = 0.38); I² = 6%	Test for overall effect: Z = 1.96 (P = 0.05)
Collins 2003	-0.14 [-0.33, 0.06]	Heterogeneity: Tau² = 0.01; Chi² = 6.39, df = 5 (P = 0.27); I² = 22%	Test for overall effect: Z = 1.39 (P = 0.16)
Gardner 2008	-0.15 [-0.35, 0.05]	Heterogeneity: Tau² = 0.01; Chi² = 6.21, df = 5 (P = 0.29); I² = 19%	Test for overall effect: Z = 1.50 (P = 0.13)
Goldenberg 2012	-0.25 [-0.41, -0.09]	Heterogeneity: Tau² = 0.00; Chi² = 1.00, df = 5 (P = 0.96); I² = 0%	Test for overall effect: Z = 3.13 (P = 0.002)
Gresele 2012	-0.02 [-0.25, 0.20]	Heterogeneity: Tau² = 0.00; Chi² = 3.42, df = 5 (P = 0.64); I² = 0%	Test for overall effect: Z = 0.20 (P = 0.84)
Hiatt 2011	-0.12 [-0.31, 0.07]	Heterogeneity: Tau² = 0.01; Chi² = 5.89, df = 5 (P = 0.32); I² = 15%	Test for overall effect: Z = 1.25 (P = 0.21)
Maxwell 2000	-0.14 [-0.36, 0.08]	Heterogeneity: Tau² = 0.02; Chi² = 6.32, df = 4 (P = 0.18); I² = 37%	Test for overall effect: Z = 1.23 (P = 0.22)

Table S9. Impact of dietary sources of NO upregulation on quality of life (WIQ Walking Distance) - leave-one-out (LOO) sensitivity analyses.

Study excluded	Effect size estimate SMD [95% CI]	Heterogeneity	Test for overall effect (Z)
None	0.04 [-0.16, 0.24]	Heterogeneity: Tau² = 0.00; Chi² = 2.05, df = 5 (P = 0.84); I² = 0%	Test for overall effect: Z = 0.37 (P = 0.71)
Collins 2003	0.06 [-0.15, 0.27]	Heterogeneity: Tau² = 0.00; Chi² = 1.31, df = 4 (P = 0.86); I² = 0%	Test for overall effect: Z = 0.56 (P = 0.57)
Gardner 2008	0.05 [-0.16, 0.27]	Heterogeneity: Tau² = 0.00; Chi² = 1.89, df = 4 (P = 0.76); I² = 0%	Test for overall effect: Z = 0.49 (P = 0.62)
Goldenberg 2012	0.03 [-0.22, 0.28]	Heterogeneity: Tau² = 0.00; Chi² = 2.03, df = 4 (P = 0.73); I² = 0%	Test for overall effect: Z = 0.21 (P = 0.83)
Grenon 2015	0.06 [-0.16, 0.29]	Heterogeneity: Tau² = 0.00; Chi² = 1.80, df = 4 (P = 0.77); I² = 0%	Test for overall effect: Z = 0.55 (P = 0.58)
Hiatt 2011	-0.01 [-0.22, 0.21]	Heterogeneity: Tau² = 0.00; Chi² = 1.15, df = 4 (P = 0.89); I² = 0%	Test for overall effect: Z = 0.04 (P = 0.96)
Ramirez 2019	0.02 [-0.18, 0.23]	Heterogeneity: Tau² = 0.00; Chi² = 1.76, df = 4 (P = 0.78); I² = 0%	Test for overall effect: Z = 0.22 (P = 0.82)

Table S10. Impact of dietary sources of NO upregulation on quality of life (WIQ Walking Speed) - leave-one-out (LOO) sensitivity analyses.

Study excluded	Effect size estimate SMD [95% CI]	Heterogeneity	Test for overall effect (Z)
None	-0.00 [-0.24, 0.23]	Heterogeneity: Tau² = 0.00; Chi² = 7.28, df = 7 (P = 0.40); I² = 4%	Test for overall effect: Z = 0.00 (P = 1.00)

Collins 2003	-0.02 [-0.31, 0.27]	Heterogeneity: Tau² = 0.03; Chi² = 7.28, df = 6 (P = 0.30); I² = 18%	Test for overall effect: Z = 0.12 (P = 0.91)
Gardner 2008	-0.02 [-0.31, 0.27]	Heterogeneity: Tau² = 0.03; Chi² = 7.28, df = 6 (P = 0.30); I² = 18%	Test for overall effect: Z = 0.12 (P = 0.91)
Grenon 2015	0.02 [-0.28, 0.31]	Heterogeneity: Tau² = 0.02; Chi² = 6.97, df = 6 (P = 0.32); I² = 14%	Test for overall effect: Z = 0.12 (P = 0.91)
Hiatt 2011	-0.14 [-0.40, 0.11]	Heterogeneity: Tau² = 0.00; Chi² = 1.06, df = 6 (P = 0.98); I² = 0%	Test for overall effect: Z = 1.10 (P = 0.27)
Oka 2005	0.07 [-0.23, 0.37]	Heterogeneity: Tau² = 0.03; Chi² = 5.34, df = 4 (P = 0.25); I² = 25%	Test for overall effect: Z = 0.44 (P = 0.66)
Ramirez 2019	0.00 [-0.27, 0.27]	Heterogeneity: Tau² = 0.02; Chi² = 7.15, df = 6 (P = 0.31); I² = 16%	Test for overall effect: Z = 0.01 (P = 0.99)

Table S11. Impact of dietary sources of NO upregulation on ABI - leave-one-out (LOO) sensitivity analyses.

Study excluded	Effect size estimate SMD [95% CI]	Heterogeneity	Test for overall effect (Z)
None	0.31 [-0.19, 0.81]	Heterogeneity: Tau² = 0.59; Chi² = 118.63, df = 10 (P < 0.00001); I² = 92%	Test for overall effect: Z = 1.21 (P = 0.23)
Bock 2018	0.33 [-0.20, 0.86]	Heterogeneity: Tau² = 0.62; Chi² = 118.45, df = 9 (P < 0.00001); I² = 92%	Test for overall effect: Z = 1.22 (P = 0.22)
Brevetti 1988	0.31 [-0.22, 0.84]	Heterogeneity: Tau² = 0.62; Chi² = 118.63, df = 9 (P < 0.00001); I² = 92%	Test for overall effect: Z = 1.16 (P = 0.25)
Collins 2003	0.45 [-0.06, 0.95]	Heterogeneity: Tau² = 0.56; Chi² = 107.99, df = 9 (P < 0.00001); I² = 92%	Test for overall effect: Z = 1.72 (P = 0.09)
Dal Lago 1999	0.37 [-0.15, 0.90]	Heterogeneity: Tau² = 0.61; Chi² = 116.37, df = 9 (P < 0.00001); I² = 92%	Test for overall effect: Z = 1.39 (P = 0.16)
Grenon 2015	0.33 [-0.23, 0.89]	Heterogeneity: Tau² = 0.69; Chi² = 117.97, df = 9 (P < 0.00001); I² = 92%	Test for overall effect: Z = 1.16 (P = 0.25)
Gresele 2012	0.32 [-0.33, 0.98]	Heterogeneity: Tau² = 0.97; Chi² = 112.76, df = 9 (P < 0.00001); I² = 92%	Test for overall effect: Z = 0.97 (P = 0.33)
Luo 2013	0.32 [-0.31, 0.95]	Heterogeneity: Tau² = 0.89; Chi² = 117.10, df = 9 (P < 0.00001); I² = 92%	Test for overall effect: Z = 1.00 (P = 0.32)
Ramirez 2019	0.38 [-0.15, 0.90]	Heterogeneity: Tau² = 0.61; Chi² = 115.82, df = 9 (P < 0.00001); I² = 92%	Test for overall effect: Z = 1.40 (P = 0.16)
Santo 2006	0.06 [-0.29, 0.40]	Heterogeneity: Tau² = 0.20; Chi² = 44.51, df = 9 (P < 0.00001); I² = 80%	Test for overall effect: Z = 0.32 (P = 0.75)
Wilson 2007	0.19 [-0.30, 0.69]	Heterogeneity: Tau² = 0.52; Chi² = 90.27, df = 9 (P < 0.00001); I² = 90%	Test for overall effect: Z = 0.76 (P = 0.45)
Woessner 2018	0.33 [-0.20, 0.86]	Heterogeneity: Tau² = 0.62; Chi² = 118.40, df = 9 (P < 0.00001); I² = 92%	Test for overall effect: Z = 1.21 (P = 0.22)

Table S12. Impact of dietary sources of NO upregulation on adverse events - leave-one-out (LOO) sensitivity analyses.

Study excluded	Effect size estimate SMD [95% CI]	Heterogeneity	Test for overall effect (Z)
None	1.32 [0.76, 2.28]	Heterogeneity: Tau² = 0.62; Chi² = 55.46, df = 12 (P < 0.00001); I² = 78%	Test for overall effect: Z = 0.98 (P = 0.33)
Bock 2018	1.32 [0.76, 2.28]	Heterogeneity: Tau² = 0.62; Chi² = 55.46, df = 12 (P < 0.00001); I² = 78%	Test for overall effect: Z = 0.98 (P = 0.33)
Brevetti 1995	1.18 [0.68, 2.06]	Heterogeneity: Tau² = 0.57; Chi² = 49.85, df = 11 (P < 0.00001); I² = 78%	Test for overall effect: Z = 0.59 (P = 0.55)

Brevetti 1999	1.48 [1.09, 2.02]	Heterogeneity: Tau² = 0.06; Chi² = 13.89, df = 11 (P = 0.24); I² = 21%	Test for overall effect: Z = 2.48 (P = 0.01)
Coto 1992	1.40 [0.79, 2.49]	Heterogeneity: Tau² = 0.64; Chi² = 54.95, df = 11 (P < 0.00001); I² = 80%	Test for overall effect: Z = 1.14 (P = 0.25)
Gardner 2008	1.34 [0.76, 2.37]	Heterogeneity: Tau² = 0.64; Chi² = 55.46, df = 11 (P < 0.00001); I² = 80%	Test for overall effect: Z = 1.00 (P = 0.32)
Goldenberg 2012	1.40 [0.75, 2.61]	Heterogeneity: Tau² = 0.74; Chi² = 55.31, df = 11 (P < 0.00001); I² = 80%	Test for overall effect: Z = 1.07 (P = 0.28)
Gresele 2012	1.32 [0.70, 2.48]	Heterogeneity: Tau² = 0.77; Chi² = 48.89, df = 11 (P < 0.00001); I² = 77%	Test for overall effect: Z = 0.86 (P = 0.39)
Hiatt 2011	1.35 [0.75, 2.44]	Heterogeneity: Tau² = 0.67; Chi² = 55.41, df = 11 (P < 0.00001); I² = 80%	Test for overall effect: Z = 0.99 (P = 0.32)
Luo 2013	1.34 [0.72, 2.51]	Heterogeneity: Tau² = 0.75; Chi² = 54.55, df = 11 (P < 0.00001); I² = 80%	Test for overall effect: Z = 0.93 (P = 0.35)
Maxwell 2000	1.33 [0.76, 2.35]	Heterogeneity: Tau² = 0.64; Chi² = 55.46, df = 11 (P < 0.00001); I² = 80%	Test for overall effect: Z = 1.00 (P = 0.32)
Mohler 2014	1.25 [0.70, 2.23]	Heterogeneity: Tau² = 0.63; Chi² = 53.19, df = 11 (P < 0.00001); I² = 79%	Test for overall effect: Z = 0.76 (P = 0.45)
Van der Avoort 2021	1.32 [0.76, 2.28]	Heterogeneity: Tau² = 0.62; Chi² = 55.46, df = 12 (P < 0.00001); I² = 78%	Test for overall effect: Z = 0.98 (P = 0.33)
Vincent 2007	1.22 [0.71, 2.10]	Heterogeneity: Tau² = 0.58; Chi² = 51.57, df = 11 (P < 0.00001); I² = 79%	Test for overall effect: Z = 0.71 (P = 0.48)
Wilson 2007	1.22 [0.69, 2.16]	Heterogeneity: Tau² = 0.60; Chi² = 49.61, df = 11 (P < 0.00001); I² = 78%	Test for overall effect: Z = 0.67 (P = 0.50)
Woessner 2018	1.28 [0.73, 2.24]	Heterogeneity: Tau² = 0.62; Chi² = 54.80, df = 11 (P < 0.00001); I² = 80%	Test for overall effect: Z = 0.88 (P = 0.38)

Table S13. Impact of dietary sources of NO upregulation on serious adverse events - leave-one-out (LOO) sensitivity analyses.

Study excluded	Effect size estimate SMD [95% CI]	Heterogeneity	Test for overall effect (Z)
None	0.83 [0.60, 1.16]	Heterogeneity: Tau² = 0.00; Chi² = 6.60, df = 10 (P = 0.76); I² = 0%	Test for overall effect: Z = 1.09 (P = 0.27)
Bock 2018	0.83 [0.60, 1.16]	Heterogeneity: Tau² = 0.00; Chi² = 6.60, df = 10 (P = 0.76); I² = 0%	Test for overall effect: Z = 1.09 (P = 0.27)
Brevetti 1999	0.79 [0.53, 1.19]	Heterogeneity: Tau² = 0.00; Chi² = 6.43, df = 9 (P = 0.70); I² = 0%	Test for overall effect: Z = 1.13 (P = 0.26)
Collins 2003	0.84 [0.61, 1.17]	Heterogeneity: Tau² = 0.00; Chi² = 6.31, df = 9 (P = 0.71); I² = 0%	Test for overall effect: Z = 1.01 (P = 0.31)
Coto 1992	0.84 [0.61, 1.17]	Heterogeneity: Tau² = 0.00; Chi² = 6.27, df = 9 (P = 0.71); I² = 0%	Test for overall effect: Z = 1.03 (P = 0.30)
Goldenberg 2012	0.89 [0.62, 1.28]	Heterogeneity: Tau² = 0.00; Chi² = 5.78, df = 9 (P = 0.76); I² = 0%	Test for overall effect: Z = 0.63 (P = 0.53)
Gresele 2012	0.80 [0.57, 1.12]	Heterogeneity: Tau² = 0.00; Chi² = 5.71, df = 9 (P = 0.77); I² = 0%	Test for overall effect: Z = 1.30 (P = 0.19)
Hiatt 2011	0.85 [0.60, 1.19]	Heterogeneity: Tau² = 0.00; Chi² = 6.46, df = 9 (P = 0.69); I² = 0%	Test for overall effect: Z = 0.97 (P = 0.33)
Kiesewetter 1993	0.83 [0.60, 1.16]	Heterogeneity: Tau² = 0.00; Chi² = 6.60, df = 10 (P = 0.76); I² = 0%	Test for overall effect: Z = 1.09 (P = 0.27)
Leng 1997	0.83 [0.58, 1.18]	Heterogeneity: Tau² = 0.00; Chi² = 6.60, df = 9 (P = 0.68); I² = 0%	Test for overall effect: Z = 1.03 (P = 0.30)

Luo 2013	0.85 [0.61, 1.19]	Heterogeneity: Tau² = 0.00; Chi² = 6.21, df = 9 (P = 0.72); I² = 0%	Test for overall effect: Z = 0.95 (P = 0.34)
Maxwell 2000	0.83 [0.60, 1.16]	Heterogeneity: Tau² = 0.00; Chi² = 6.60, df = 10 (P = 0.76); I² = 0%	Test for overall effect: Z = 1.09 (P = 0.27)
McDermott 2020	0.80 [0.57, 1.12]	Heterogeneity: Tau² = 0.00; Chi² = 4.90, df = 9 (P = 0.84); I² = 0%	Test for overall effect: Z = 1.32 (P = 0.19)
Mohler 2014	0.86 [0.62, 1.19]	Heterogeneity: Tau² = 0.00; Chi² = 4.54, df = 9 (P = 0.87); I² = 0%	Test for overall effect: Z = 0.94 (P = 0.35)
Van der Avoort 2021	0.83 [0.60, 1.16]	Heterogeneity: Tau² = 0.00; Chi² = 6.60, df = 10 (P = 0.76); I² = 0%	Test for overall effect: Z = 1.09 (P = 0.27)
Wilson 2007	0.81 [0.58, 1.15]	Heterogeneity: Tau² = 0.00; Chi² = 6.43, df = 9 (P = 0.70); I² = 0%	Test for overall effect: Z = 1.17 (P = 0.24)
Woessner 2018	0.83 [0.60, 1.16]	Heterogeneity: Tau² = 0.00; Chi² = 6.60, df = 10 (P = 0.76); I² = 0%	Test for overall effect: Z = 1.09 (P = 0.27)

Table S14. Impact of dietary sources of NO upregulation on mortality - leave-one-out (LOO) sensitivity analyses.

Study excluded	Effect size estimate SMD [95% CI]	Heterogeneity	Test for overall effect (Z)
None	1.14 [0.49, 2.64]	Heterogeneity: Tau² = 0.00; Chi² = 1.30, df = 5 (P = 0.94); I² = 0%	Test for overall effect: Z = 0.31 (P = 0.76)
Bock 2018	1.14 [0.49, 2.64]	Heterogeneity: Tau² = 0.00; Chi² = 1.30, df = 5 (P = 0.94); I² = 0%	Test for overall effect: Z = 0.31 (P = 0.76)
Brevetti 1999	1.24 [0.40, 3.83]	Heterogeneity: Tau² = 0.00; Chi² = 1.25, df = 4 (P = 0.87); I² = 0%	Test for overall effect: Z = 0.38 (P = 0.71)
Goldenberg 2012	1.06 [0.45, 2.53]	Heterogeneity: Tau² = 0.00; Chi² = 0.89, df = 4 (P = 0.93); I² = 0%	Test for overall effect: Z = 0.13 (P = 0.89)
Gresele 2012	1.25 [0.52, 2.98]	Heterogeneity: Tau² = 0.00; Chi² = 0.69, df = 4 (P = 0.95); I² = 0%	Test for overall effect: Z = 0.50 (P = 0.61)
Leng 1997	1.12 [0.42, 2.98]	Heterogeneity: Tau² = 0.00; Chi² = 1.29, df = 4 (P = 0.86); I² = 0%	Test for overall effect: Z = 0.24 (P = 0.81)
McDermott 2020	1.07 [0.45, 2.55]	Heterogeneity: Tau² = 0.00; Chi² = 0.97, df = 4 (P = 0.91); I² = 0%	Test for overall effect: Z = 0.15 (P = 0.88)
Ramirez 2019	1.14 [0.49, 2.64]	Heterogeneity: Tau² = 0.00; Chi² = 1.30, df = 5 (P = 0.94); I² = 0%	Test for overall effect: Z = 0.31 (P = 0.76)
Santo 2006	1.14 [0.49, 2.64]	Heterogeneity: Tau² = 0.00; Chi² = 1.30, df = 5 (P = 0.94); I² = 0%	Test for overall effect: Z = 0.31 (P = 0.76)
Tenore 2019	1.14 [0.49, 2.64]	Heterogeneity: Tau² = 0.00; Chi² = 1.30, df = 5 (P = 0.94); I² = 0%	Test for overall effect: Z = 0.31 (P = 0.76)
Van der Avoort 2021	1.14 [0.49, 2.64]	Heterogeneity: Tau² = 0.00; Chi² = 1.30, df = 5 (P = 0.94); I² = 0%	Test for overall effect: Z = 0.31 (P = 0.76)
Vincent 2007	1.14 [0.49, 2.64]	Heterogeneity: Tau² = 0.00; Chi² = 1.30, df = 5 (P = 0.94); I² = 0%	Test for overall effect: Z = 0.31 (P = 0.76)
Wilson 2007	1.16 [0.48, 2.78]	Heterogeneity: Tau² = 0.00; Chi² = 1.29, df = 4 (P = 0.86); I² = 0%	Test for overall effect: Z = 0.32 (P = 0.75)
Woessner 2018	1.14 [0.49, 2.64]	Heterogeneity: Tau² = 0.00; Chi² = 1.30, df = 5 (P = 0.94); I² = 0%	Test for overall effect: Z = 0.31 (P = 0.76)

Table S15. Impact of dietary sources of NO upregulation on requirement of lower extremity revascularisation or amputation - leave-one-out (LOO) sensitivity analyses.

Study excluded	Effect size estimate SMD	Heterogeneity	Test for overall effect (Z)

	[95% CI]		
None	1.70 [0.42, 6.87]	Heterogeneity: $Tau^2 = 0.00$; $Chi^2 = 1.71$, df = 2 (P = 0.42); $I^2 = 0\%$	Test for overall effect: Z = 0.74 (P = 0.46)
Brevetti 1999	1.29 [0.25, 6.57]	Heterogeneity: $Tau^2 = 0.08$; $Chi^2 = 1.05$, df = 1 (P = 0.31); $I^2 = 5\%$	Test for overall effect: Z = 0.30 (P = 0.76)
Leng 1997	5.10 [0.58, 44.59]	Heterogeneity: $Tau^2 = 0.00$; $Chi^2 = 0.00$, df = 1 (P = 0.99); $I^2 = 0\%$	Test for overall effect: Z = 1.47 (P = 0.14)
McDermott 2020	1.35 [0.25, 7.45]	Heterogeneity: $Tau^2 = 0.20$; $Chi^2 = 1.12$, df = 1 (P = 0.29); $I^2 = 11\%$	Test for overall effect: Z = 0.35 (P = 0.73)
Park 2020	1.70 [0.42, 6.87]	Heterogeneity: $Tau^2 = 0.00$; $Chi^2 = 1.71$, df = 2 (P = 0.42); $I^2 = 0\%$	Test for overall effect: Z = 0.74 (P = 0.46)
Pekas 2021	1.70 [0.42, 6.87]	Heterogeneity: $Tau^2 = 0.00$; $Chi^2 = 1.71$, df = 2 (P = 0.42); $I^2 = 0\%$	Test for overall effect: Z = 0.74 (P = 0.46)
Van der Avoort 2021	1.70 [0.42, 6.87]	Heterogeneity: $Tau^2 = 0.00$; $Chi^2 = 1.71$, df = 2 (P = 0.42); $I^2 = 0\%$	Test for overall effect: Z = 0.74 (P = 0.46)

Section S3:

Figure S1. Risk of bias summary plot indicating the number of studies with low, some, and high risk of bias across the five domains.

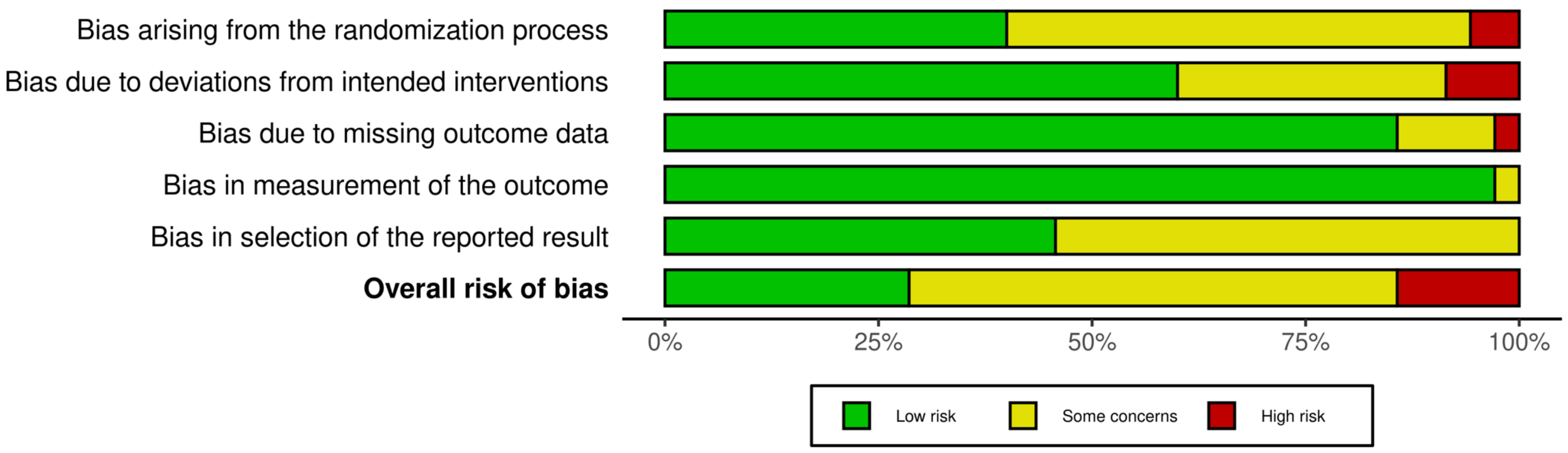

Figure S2. Forest plot showing effect of dietary supplements upregulating the NO pathway versus control on maximum walking distance; meta-analysis using fixed effect model.

	Experimental			Control				Std. Mean Difference
**Study or Subgroup**	**Mean**	**SD**	**Total**	**Mean**	**SD**	**Total**	**Weight**	**IV, Fixed, 95% CI**
Brevetti 1995	354.1	218.9	99	298.1	193	115	10.9%	0.27 [0.00, 0.54]
Brevetti 1999	342	278.2	86	269	177.2	87	8.8%	0.31 [0.01, 0.61]
Brevetti 1999	574	322.6	76	612	328.9	79	8.0%	−0.12 [−0.43, 0.20]
Collins 2003	534.9	441.4	13	501.9	427.8	10	1.2%	0.07 [−0.75, 0.90]
Coto 1992	270.6	78.5	142	208.8	68.4	140	13.3%	0.84 [0.59, 1.08]
Dal Lago 1999	360.8	228.9	9	237.3	108.1	10	0.9%	0.67 [−0.26, 1.60]
Goldenberg 2012	373.4	98.7	74	321.3	103.5	71	7.2%	0.51 [0.18, 0.84]
Gresele 2012	327	244	221	344	254	221	22.7%	−0.07 [−0.25, 0.12]
Kenjale 2011	476.5	208.3	4	417.5	199.4	4	0.4%	0.25 [−1.15, 1.65]
Loffredo 2014	122.2	61.5	10	109.1	65.1	10	1.0%	0.20 [−0.68, 1.08]
Luo 2013	255.7	158.3	120	202.1	140.4	119	12.1%	0.36 [0.10, 0.61]
Maxwell 2000	339	310.6	15	236	147.8	6	0.9%	0.36 [−0.60, 1.31]
Maxwell 2000	327	239	12	236	147.8	7	0.9%	0.41 [−0.53, 1.35]
McDermott 2020	490	237.8	18	523.9	459.6	20	1.9%	−0.09 [−0.73, 0.55]
Micker 2007	687.1	769.7	24	137.8	86.5	24	2.2%	0.99 [0.38, 1.59]
Oka 2005	398.5	208.8	18	352.9	152	6	0.9%	0.22 [−0.70, 1.15]
Oka 2005	371.4	188.9	17	352.9	152	6	0.9%	0.10 [−0.83, 1.03]
Oka 2005	372.1	222.1	19	352.9	152	6	0.9%	0.09 [−0.83, 1.01]
Park 2020	487.8	218.4	5	435.4	218.1	6	0.6%	0.22 [−0.97, 1.41]
Pekas 2021	460.5	279.3	5	361.6	263.1	6	0.5%	0.33 [−0.87, 1.53]
van der Avoort 2021	662.2	195.6	6	618.7	197.3	3	0.4%	0.20 [−1.19, 1.59]
van der Avoort 2021	663.1	156.4	6	618.7	197.3	3	0.4%	0.23 [−1.16, 1.63]
Wilson 2007	314	25	58	392	28	61	2.9%	−2.92 [−3.44, −2.39]
**Total (95% CI)**			**1057**			**1020**	**100.0%**	**0.18 [0.10, 0.27]**

Heterogeneity: $Chi^2 = 190.04$, df = 22 ($P < 0.00001$); $I^2 = 88\%$
Test for overall effect: Z = 4.06 ($P < 0.0001$)

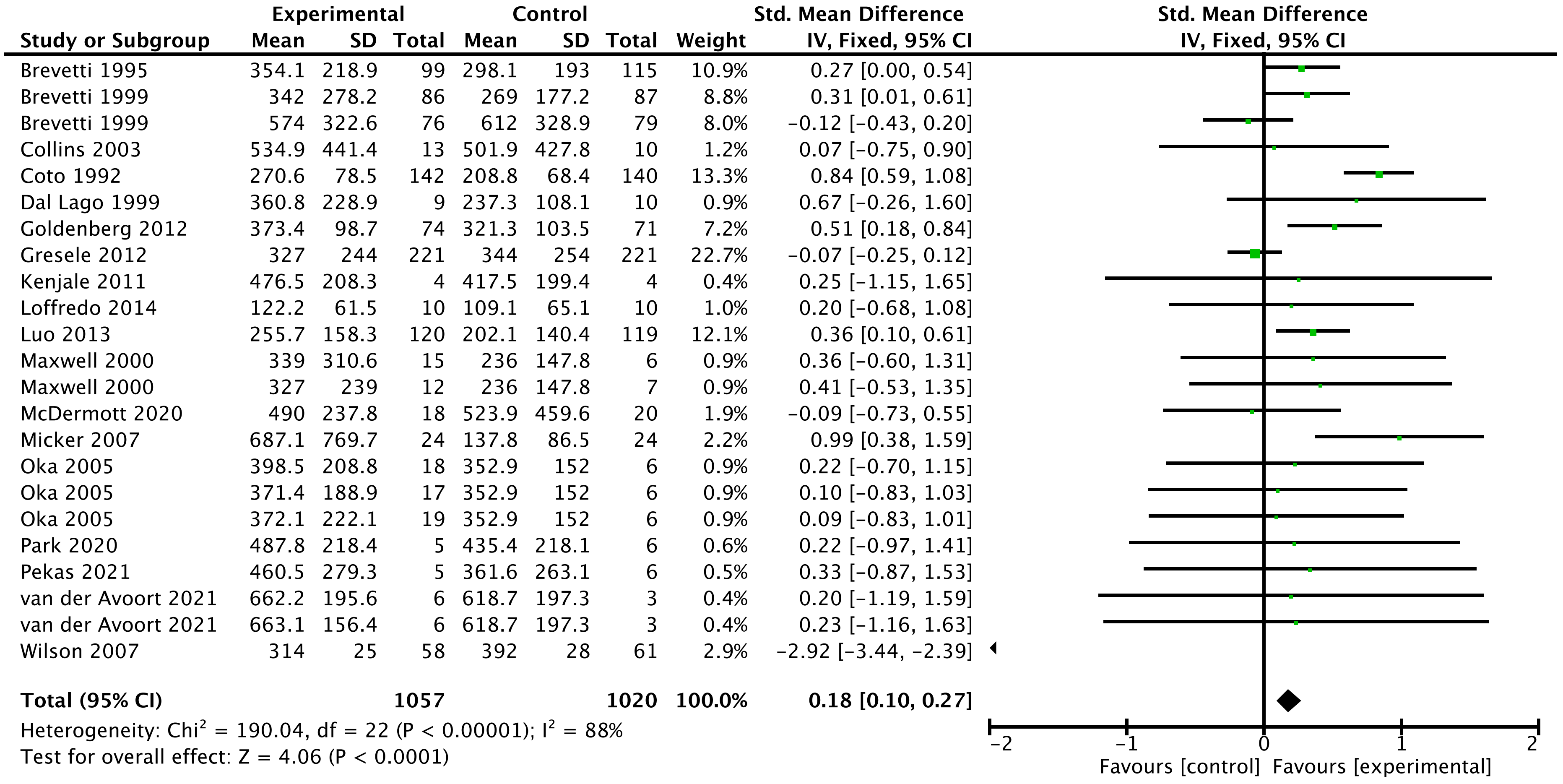

Figure S3. Funnel plot showing the impact of dietary supplements upregulating the NO pathway on maximum walking distance.

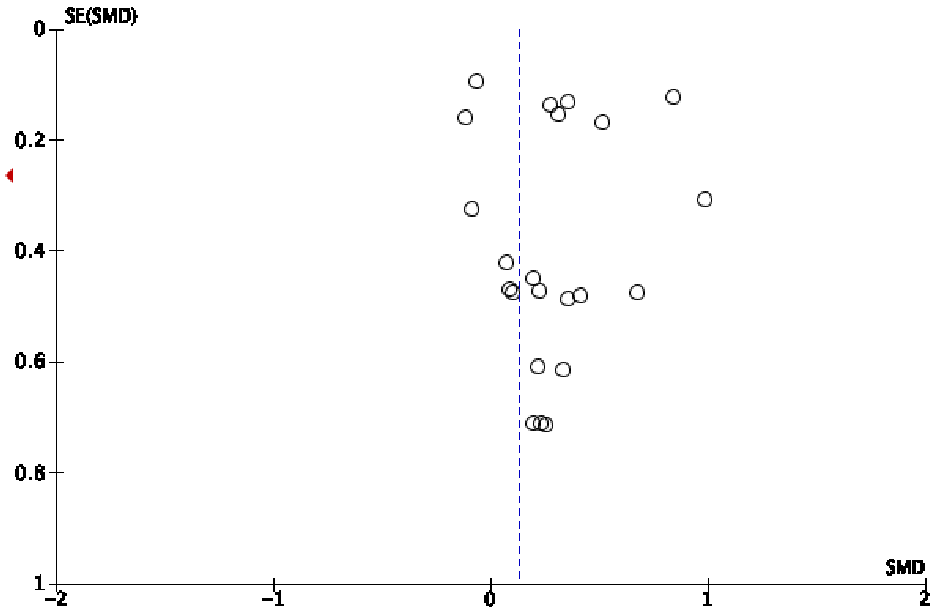

Figure S4. Forest plot showing effect of dietary supplements upregulating the NO pathway versus control on maximum walking distance; studies stratified by intervention type.

	Experimental			Control				Std. Mean Difference
**Study or Subgroup**	**Mean**	**SD**	**Total**	**Mean**	**SD**	**Total**	**Weight**	**IV, Random, 95% CI**
**1.6.1 Nitric oxide donors**								
Gresele 2012	327	244	221	344	254	221	5.9%	−0.07 [−0.25, 0.12]
Kenjale 2011	476.5	208.3	4	417.5	199.4	4	2.6%	0.25 [−1.15, 1.65]
Pekas 2021	460.5	279.3	5	361.6	263.1	6	3.1%	0.33 [−0.87, 1.53]
van der Avoort 2021	662.2	195.6	6	618.7	197.3	3	2.6%	0.20 [−1.19, 1.59]
van der Avoort 2021	663.1	156.4	6	618.7	197.3	3	2.6%	0.23 [−1.16, 1.63]
**Subtotal (95% CI)**			**242**			**237**	**16.9%**	**−0.04 [−0.22, 0.14]**
Heterogeneity: $Tau^2 = 0.00$; $Chi^2 = 0.89$, df = 4 (P = 0.93); $I^2 = 0\%$								
Test for overall effect: Z = 0.48 (P = 0.63)								
**1.6.2 Antioxidants**								
Brevetti 1995	354.1	218.9	99	298.1	193	115	5.7%	0.27 [0.00, 0.54]
Brevetti 1999	342	278.2	86	269	177.2	87	5.7%	0.31 [0.01, 0.61]
Brevetti 1999	574	322.6	76	612	328.9	79	5.7%	−0.12 [−0.43, 0.20]
Collins 2003	534.9	441.4	13	501.9	427.8	10	4.2%	0.07 [−0.75, 0.90]
Coto 1992	270.6	78.5	142	208.8	68.4	140	5.8%	0.84 [0.59, 1.08]
Dal Lago 1999	360.8	228.9	9	237.3	108.1	10	3.8%	0.67 [−0.26, 1.60]
Goldenberg 2012	373.4	98.7	74	321.3	103.5	71	5.6%	0.51 [0.18, 0.84]
Luo 2013	255.7	158.3	120	202.1	140.4	119	5.8%	0.36 [0.10, 0.61]
Park 2020	487.8	218.4	5	435.4	218.1	6	3.1%	0.22 [−0.97, 1.41]
**Subtotal (95% CI)**			**624**			**637**	**45.4%**	**0.36 [0.14, 0.59]**
Heterogeneity: $Tau^2 = 0.07$; $Chi^2 = 25.41$, df = 8 (P = 0.001); $I^2 = 69\%$								
Test for overall effect: Z = 3.18 (P = 0.001)								
**1.6.3 Enhancers of NO availability**								
Maxwell 2000	339	310.6	15	236	147.8	6	3.8%	0.36 [−0.60, 1.31]
Maxwell 2000	327	239	12	236	147.8	7	3.8%	0.41 [−0.53, 1.35]
Micker 2007	687.1	769.7	24	137.8	86.5	24	4.9%	0.99 [0.38, 1.59]
Oka 2005	371.4	188.9	17	352.9	152	6	3.8%	0.10 [−0.83, 1.03]
Oka 2005	372.1	222.1	19	352.9	152	6	3.9%	0.09 [−0.83, 1.01]
Oka 2005	398.5	208.8	18	352.9	152	6	3.8%	0.22 [−0.70, 1.15]
Wilson 2007	314	25	58	392	28	61	5.1%	−2.92 [−3.44, −2.39]
**Subtotal (95% CI)**			**163**			**116**	**29.0%**	**−0.12 [−1.43, 1.19]**
Heterogeneity: $Tau^2 = 2.95$; $Chi^2 = 118.16$, df = 6 (P < 0.00001); $I^2 = 95\%$								
Test for overall effect: Z = 0.18 (P = 0.86)								
**1.6.4 Nitric oxide synthase inducers**								
Loffredo 2014	122.2	61.5	10	109.1	65.1	10	4.0%	0.20 [−0.68, 1.08]
McDermott 2020	490	237.8	18	523.9	459.6	20	4.7%	−0.09 [−0.73, 0.55]
**Subtotal (95% CI)**			**28**			**30**	**8.7%**	**0.01 [−0.51, 0.53]**
Heterogeneity: $Tau^2 = 0.00$; $Chi^2 = 0.27$, df = 1 (P = 0.60); $I^2 = 0\%$								
Test for overall effect: Z = 0.04 (P = 0.97)								
**Total (95% CI)**			**1057**			**1020**	**100.0%**	**0.13 [−0.17, 0.43]**
Heterogeneity: $Tau^2 = 0.39$; $Chi^2 = 190.04$, df = 22 (P < 0.00001); $I^2 = 88\%$								
Test for overall effect: Z = 0.86 (P = 0.39)								
Test for subgroup differences: $Chi^2 = 8.02$, df = 3 (P = 0.05), $I^2 = 62.6\%$								

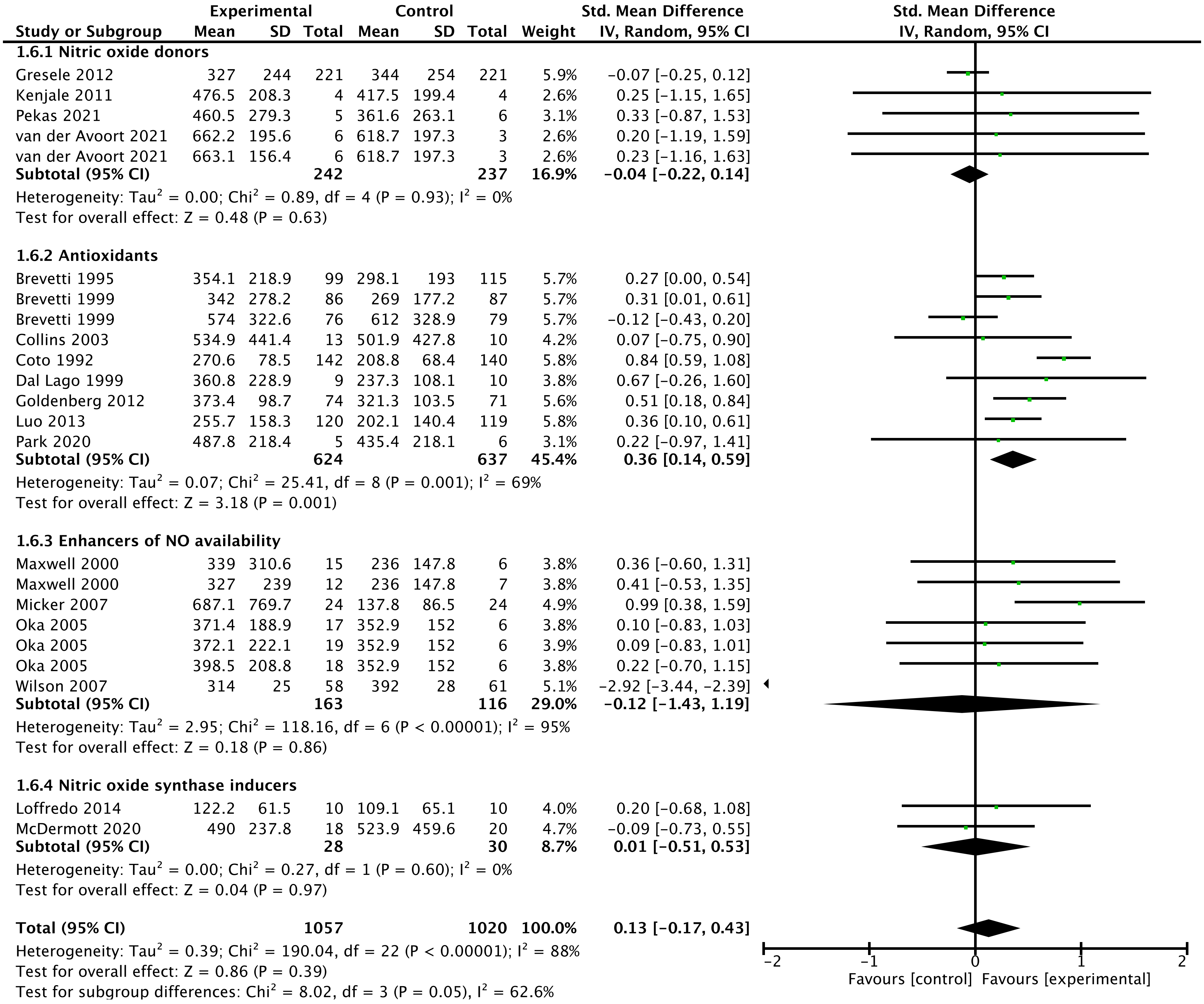

Figure S5. Funnel plot showing the impact of dietary supplements upregulating the NO pathway on initial claudication distance.

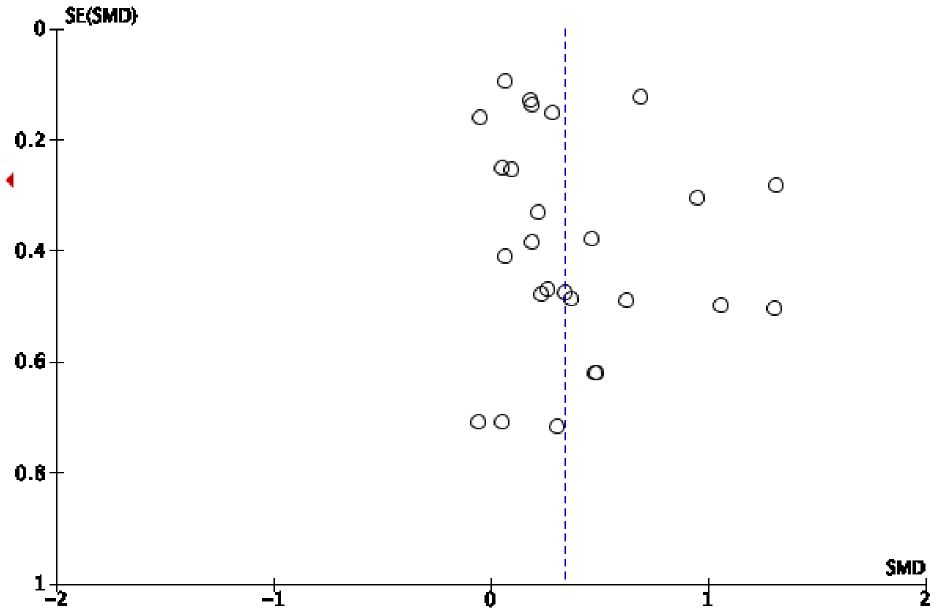

Figure S6. Forest plot showing effect of dietary supplements upregulating the NO pathway versus control on initial claudication distance; studies stratified by intervention type.

	Experimental			Control				Std. Mean Difference
Study or Subgroup	Mean	SD	Total	Mean	SD	Total	Weight	IV, Random, 95% CI
**1.3.1 Nitric oxide donors**								
Gresele 2012	203	162.6	221	193.2	138.8	221	4.7%	0.06 [−0.12, 0.25]
Kenjale 2011	192.2	88.5	4	163.6	75.1	4	2.4%	0.30 [−1.10, 1.70]
Pekas 2021	215.6	91.8	5	161.5	108.9	6	2.8%	0.49 [−0.73, 1.70]
van der Avoort 2021	367.1	166.2	6	358.2	156.4	3	2.5%	0.05 [−1.34, 1.43]
van der Avoort 2021	348.4	136.9	6	358.2	156.4	3	2.5%	−0.06 [−1.45, 1.33]
Subtotal (95% CI)			242			237	14.8%	0.08 [−0.10, 0.26]
Heterogeneity: $Tau^2 = 0.00$; $Chi^2 = 0.59$, df = 4 (P = 0.96); $I^2 = 0\%$								
Test for overall effect: Z = 0.82 (P = 0.41)								
**1.3.2 Antioxidants**								
Brevetti 1988	306.5	121.8	10	174.7	63.1	10	3.2%	1.30 [0.32, 2.29]
Brevetti 1995	222.6	179.1	99	191.6	150.1	115	4.6%	0.19 [−0.08, 0.46]
Brevetti 1999	367	270.3	76	381	293.3	79	4.6%	−0.05 [−0.36, 0.27]
Brevetti 1999	193	157.7	86	156	93.3	87	4.6%	0.28 [−0.01, 0.58]
Coto 1992	153.3	55.6	142	117.3	48.8	140	4.7%	0.69 [0.45, 0.93]
Dal Lago 1999	163.3	53.3	9	112.2	38.8	10	3.3%	1.06 [0.08, 2.03]
Gardner 2008	78.7	60.3	31	73	58.7	31	4.3%	0.09 [−0.40, 0.59]
Hiatt 2011	217.3	71	32	143	33.5	30	4.2%	1.31 [0.76, 1.86]
Kiesewetter 1993	207.1	85	32	203.1	72.8	32	4.3%	0.05 [−0.44, 0.54]
Luo 2013	148.4	94.8	120	133.2	71.5	119	4.7%	0.18 [−0.07, 0.43]
Park 2020	220.4	136.4	5	151.5	127.4	6	2.8%	0.48 [−0.73, 1.69]
Ramirez 2019	225	168	11	213	195	13	3.6%	0.06 [−0.74, 0.87]
Santo 2006	519.8	9	37	331.8	86.4	37	3.9%	3.03 [2.35, 3.71]
Vincent 2007	233.4	159.1	16	201.9	162.1	12	3.8%	0.19 [−0.56, 0.94]
Subtotal (95% CI)			706			721	56.4%	0.58 [0.26, 0.90]
Heterogeneity: $Tau^2 = 0.29$; $Chi^2 = 96.62$, df = 13 (P < 0.00001); $I^2 = 87\%$								
Test for overall effect: Z = 3.52 (P = 0.0004)								
**1.3.3 Enhancers of NO availability**								
Domingues 2021	193	110	14	145	90	15	3.8%	0.47 [−0.27, 1.21]
Maxwell 2000	221	258.2	15	135	79.3	6	3.3%	0.37 [−0.59, 1.32]
Maxwell 2000	260	232.1	12	135	79.3	7	3.3%	0.62 [−0.34, 1.58]
Micker 2007	438.8	532.6	24	74.2	42.3	24	4.1%	0.95 [0.35, 1.55]
Oka 2005	191.6	114.4	18	152.6	101.8	6	3.4%	0.34 [−0.59, 1.27]
Oka 2005	179.2	115.6	17	152.6	101.8	6	3.3%	0.23 [−0.71, 1.16]
Oka 2005	198	181.6	19	152.6	101.8	6	3.4%	0.26 [−0.66, 1.18]
Wilson 2007	110	11	58	146	12	61	4.2%	−3.10 [−3.64, −2.57]
Subtotal (95% CI)			177			131	28.7%	0.00 [−1.21, 1.21]
Heterogeneity: $Tau^2 = 2.86$; $Chi^2 = 134.50$, df = 7 (P < 0.00001); $I^2 = 95\%$								
Test for overall effect: Z = 0.00 (P = 1.00)								
**1.3.4 Nitric oxide synthase inducers**								
McDermott 2020	333.5	204.7	18	291.5	171.7	19	0.0%	0.22 [−0.43, 0.86]
Subtotal (95% CI)			0			0		Not estimable
Heterogeneity: Not applicable								
Test for overall effect: Not applicable								
**Total (95% CI)**			1125			1089	100.0%	0.35 [0.04, 0.66]
Heterogeneity: $Tau^2 = 0.52$; $Chi^2 = 263.13$, df = 26 (P < 0.00001); $I^2 = 90\%$								
Test for overall effect: Z = 2.20 (P = 0.03)								
Test for subgroup differences: $Chi^2 = 7.23$, df = 2 (P = 0.03), $I^2 = 72.3\%$								

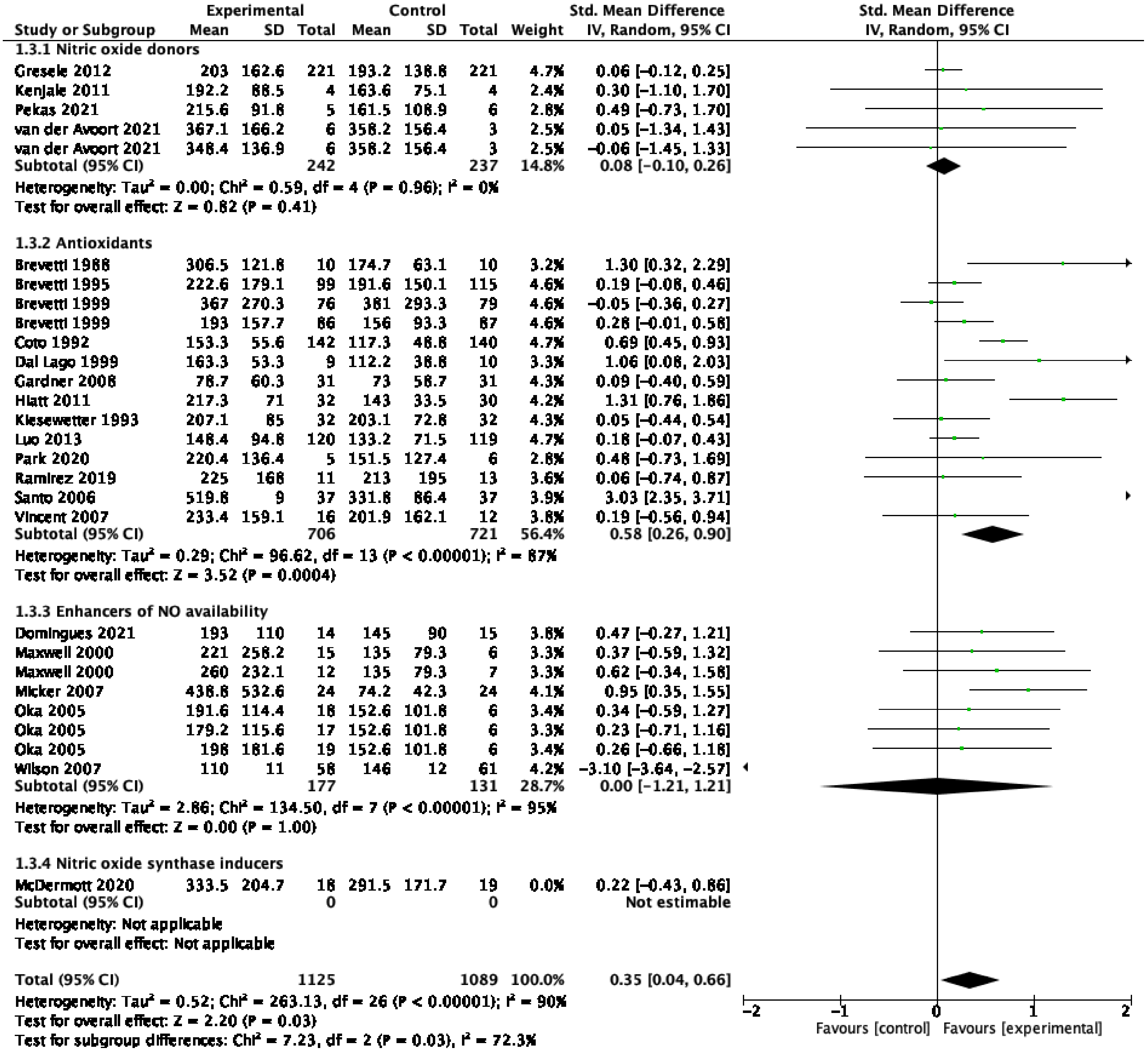

Figure S7. Forest plot showing effect of dietary supplements upregulating the NO pathway versus control on 6-minute walking distance.

	Experimental			Control				Std. Mean Difference
Study or Subgroup	Mean	SD	Total	Mean	SD	Total	Weight	IV, Random, 95% CI
Bock 2018	425	82	13	427	66	8	11.3%	−0.03 [−0.91, 0.86]
Domingues 2021	369	115	14	382	99	15	16.5%	−0.12 [−0.85, 0.61]
McDermott 2017	369.7	64.9	20	353.8	70.4	10	15.1%	0.23 [−0.53, 0.99]
McDermott 2017	357.4	57.7	23	353.8	70.4	11	17.0%	0.06 [−0.66, 0.78]
McDermott 2020	346.6	74.2	22	336.4	88.3	21	24.5%	0.12 [−0.48, 0.72]
Vincent 2007	380.7	126.6	16	395.8	83.8	12	15.6%	−0.13 [−0.88, 0.62]
Woessner 2018	371.8	67.5	11	378.8	106.7	13	0.0%	−0.07 [−0.88, 0.73]
**Total (95% CI)**			108			77	100.0%	0.03 [−0.26, 0.33]
Heterogeneity: $Tau^2 = 0.00$; $Chi^2 = 0.72$, df = 5 (P = 0.98); $I^2 = 0\%$								
Test for overall effect: Z = 0.21 (P = 0.83)								

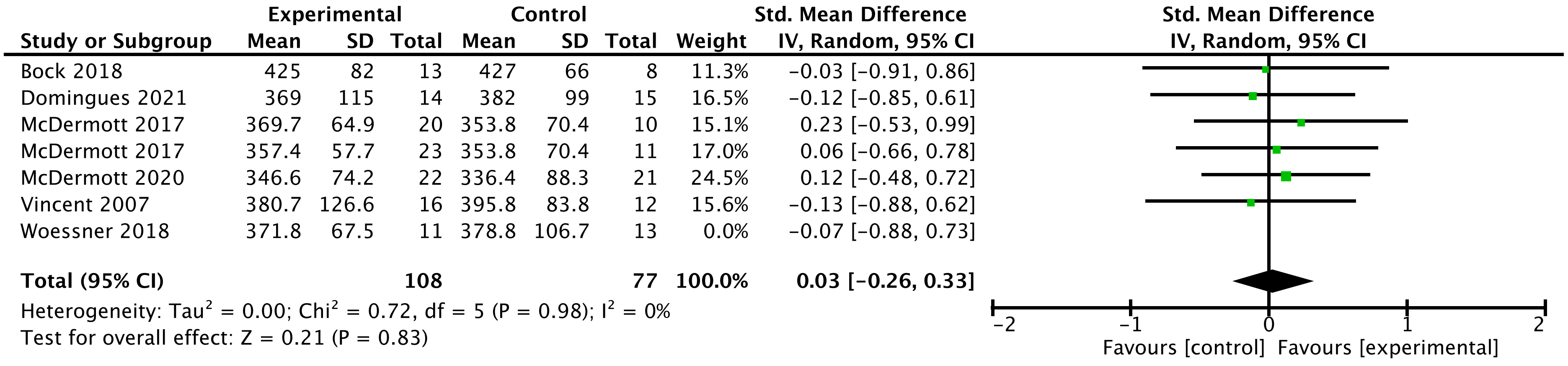

Figure S8. Funnel plot showing the impact of dietary supplements upregulating the NO pathway on 6-minute walking distance.

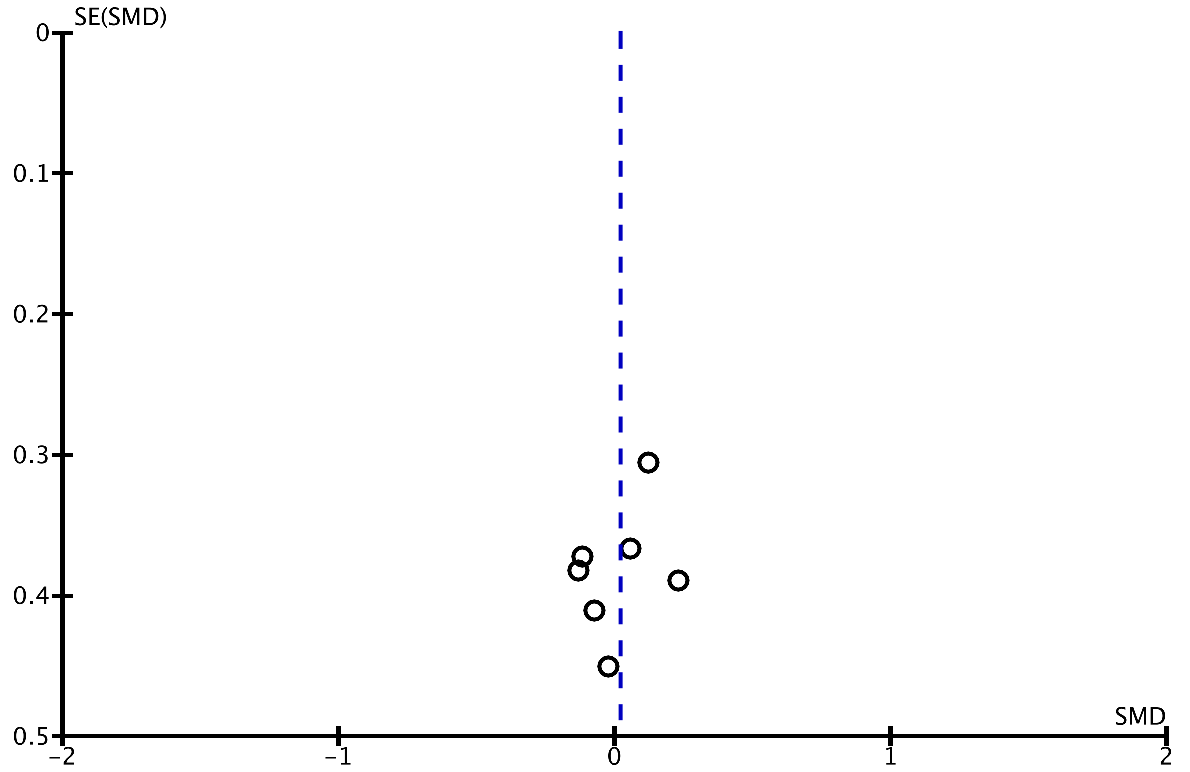

Figure S9. Forest plot showing effect of dietary supplements upregulating the NO pathway versus control on 6-minute walking distance; studies stratified by risk of bias.

	Experimental			Control				Std. Mean Difference
**Study or Subgroup**	**Mean**	**SD**	**Total**	**Mean**	**SD**	**Total**	**Weight**	**IV, Random, 95% CI**
**1.8.1 Low Risk of Bias**								
Bock 2018	425	82	13	427	66	8	10.0%	−0.03 [−0.91, 0.86]
McDermott 2017	369.7	64.9	20	353.8	70.4	10	13.3%	0.23 [−0.53, 0.99]
McDermott 2017	357.4	57.7	23	353.8	70.4	11	15.0%	0.06 [−0.66, 0.78]
McDermott 2020	346.6	74.2	22	336.4	88.3	21	21.6%	0.12 [−0.48, 0.72]
**Subtotal (95% CI)**			**78**			**50**	**59.8%**	**0.11 [−0.25, 0.47]**
Heterogeneity: $Tau^2 = 0.00$; $Chi^2 = 0.21$, df = 3 (P = 0.98); $I^2 = 0\%$								
Test for overall effect: Z = 0.58 (P = 0.56)								
**1.8.2 Some Risk of Bias**								
Domingues 2021	369	115	14	382	99	15	14.5%	−0.12 [−0.85, 0.61]
Vincent 2007	380.7	126.6	16	395.8	83.8	12	13.7%	−0.13 [−0.88, 0.62]
Woessner 2018	371.8	67.5	11	378.8	106.7	13	12.0%	−0.07 [−0.88, 0.73]
**Subtotal (95% CI)**			**41**			**40**	**40.2%**	**−0.11 [−0.55, 0.33]**
Heterogeneity: $Tau^2 = 0.00$; $Chi^2 = 0.01$, df = 2 (P = 0.99); $I^2 = 0\%$								
Test for overall effect: Z = 0.49 (P = 0.62)								
**1.8.3 High Risk of Bias**								
**Subtotal (95% CI)**			**0**			**0**		**Not estimable**
Heterogeneity: Not applicable								
Test for overall effect: Not applicable								
**Total (95% CI)**			**119**			**90**	**100.0%**	**0.02 [−0.26, 0.30]**
Heterogeneity: $Tau^2 = 0.00$; $Chi^2 = 0.78$, df = 6 (P = 0.99); $I^2 = 0\%$								
Test for overall effect: Z = 0.13 (P = 0.89)								
Test for subgroup differences: $Chi^2 = 0.56$, df = 1 (P = 0.45), $I^2 = 0\%$								

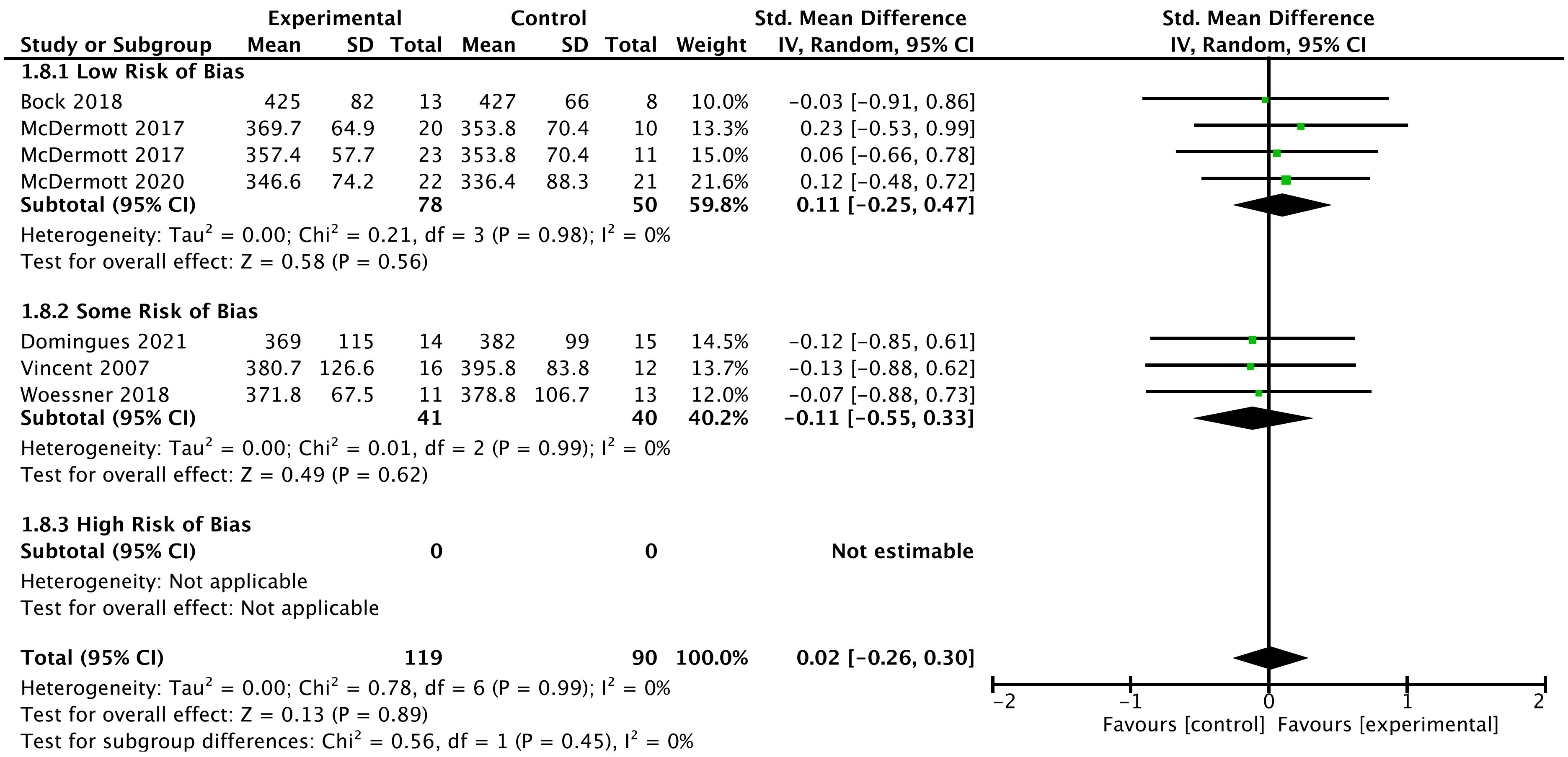

Figure S10. Funnel plot showing the impact of dietary supplements upregulating the NO pathway on quality of life (as measured by the SF-36 physical function domain).

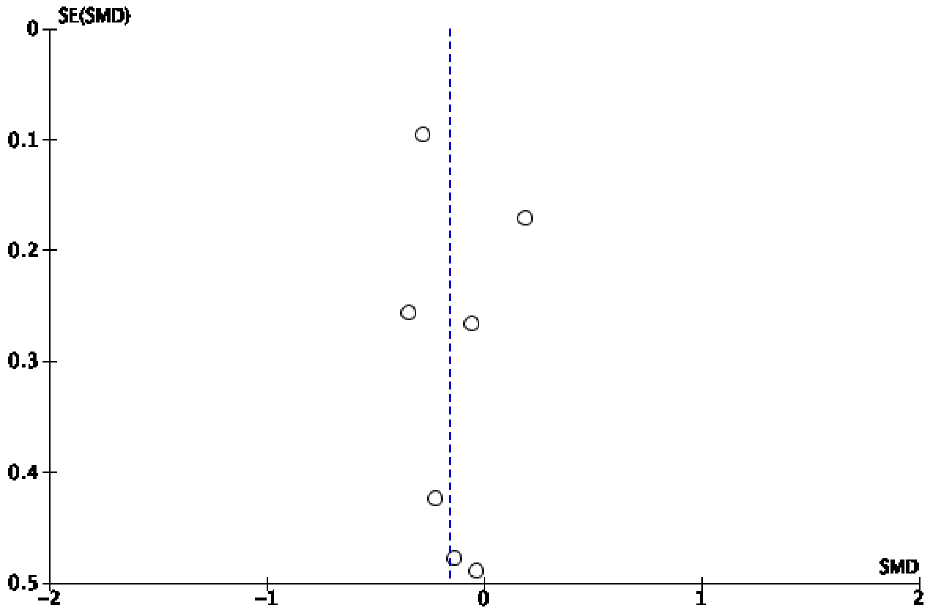

Figure S11. Forest plot showing effect of dietary supplements upregulating the NO pathway versus control on quality of life (as measured by the WIQ walking distance domain).

	Experimental			Control				Std. Mean Difference
Study or Subgroup	Mean	SD	Total	Mean	SD	Total	Weight	IV, Random, 95% CI
Collins 2003	0.26	0.28	13	0.36	0.34	10	5.9%	−0.31 [−1.14, 0.52]
Gardner 2008	0.52	0.32	30	0.54	0.33	26	14.7%	−0.06 [−0.59, 0.46]
Goldenberg 2012	35.4	29.4	68	33.8	27.1	68	35.9%	0.06 [−0.28, 0.39]
Grenon 2015	27	36.3	40	29	29.4	40	21.1%	−0.06 [−0.50, 0.38]
Hiatt 2011	49.5	24.2	32	42.8	26.9	30	16.2%	0.26 [−0.24, 0.76]
Ramirez 2019	53	38.7	11	43	38.2	13	6.2%	0.25 [−0.56, 1.06]
Total (95% CI)			194			187	100.0%	0.04 [−0.16, 0.24]
Heterogeneity: Tau² = 0.00; Chi² = 2.05, df = 5 (P = 0.84); I² = 0%								
Test for overall effect: Z = 0.37 (P = 0.71)								

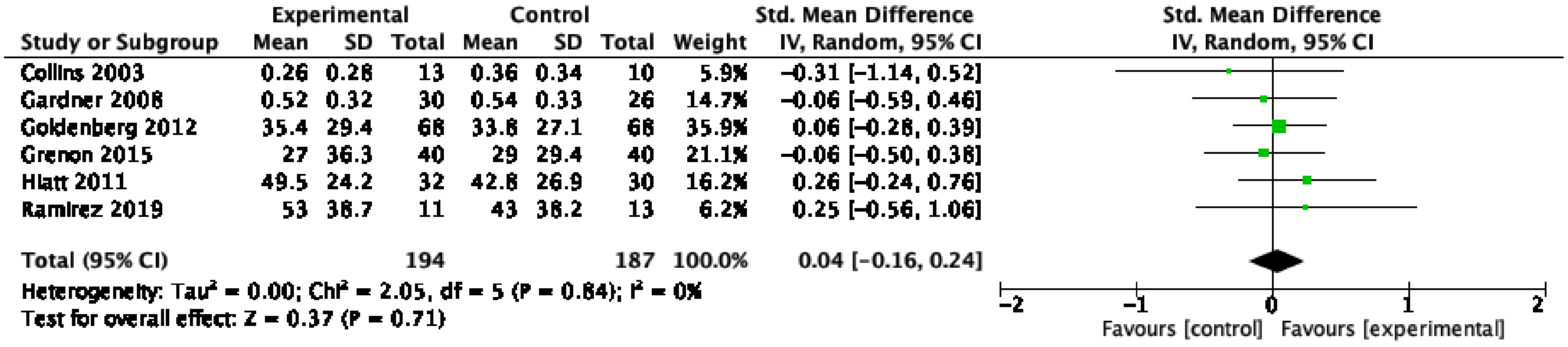

Figure S12. Funnel plot showing the impact of dietary supplements upregulating the NO pathway on quality of life (as measured by the WIQ walking distance domain).

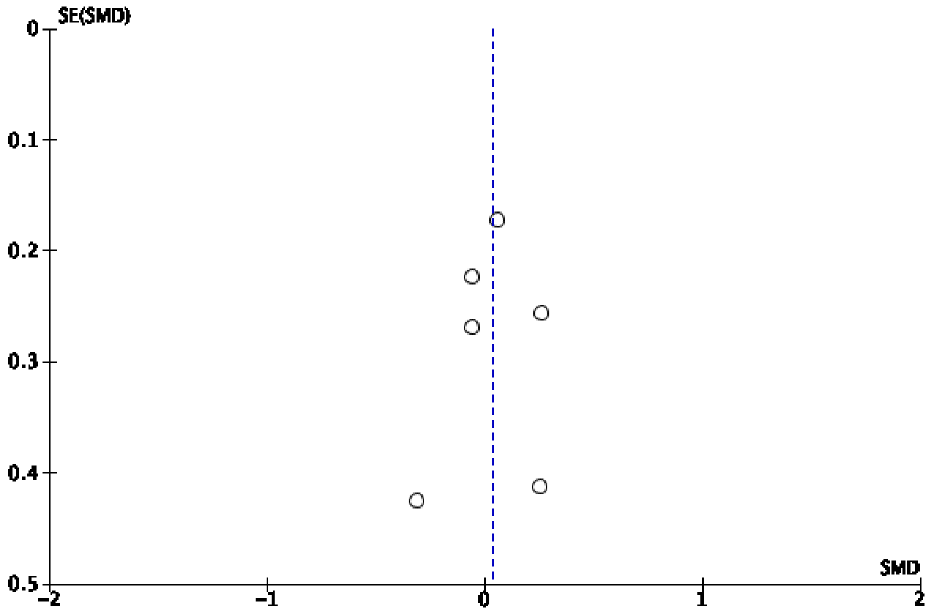

Figure S13. Forest plot showing effect of dietary supplements upregulating the NO pathway versus control on quality of life (as measured by the WIQ walking speed domain).

	Experimental			Control				Std. Mean Difference
Study or Subgroup	Mean	SD	Total	Mean	SD	Total	Weight	IV, Random, 95% CI
Collins 2003	0.27	0.17	13	0.33	0.32	10	7.9%	−0.24 [−1.06, 0.59]
Gardner 2008	0.5	0.23	30	0.5	0.26	26	18.8%	0.00 [−0.53, 0.53]
Grenon 2015	26	27.8	40	29	29.4	40	26.3%	−0.10 [−0.54, 0.33]
Hiatt 2011	42.8	26.9	32	29.4	17.2	30	19.9%	0.58 [0.07, 1.09]
Oka 2005	39.3	25	19	42.1	27.6	6	6.4%	−0.11 [−1.02, 0.81]
Oka 2005	29.6	27.6	18	42.1	27.6	6	6.2%	−0.44 [−1.37, 0.50]
Oka 2005	32.1	22.2	17	42.1	27.6	6	6.1%	−0.41 [−1.35, 0.53]
Ramirez 2019	38.3	21.8	11	42.4	32.7	13	8.3%	−0.14 [−0.94, 0.66]
Total (95% CI)			180			137	100.0%	−0.00 [−0.24, 0.23]
Heterogeneity: Tau² = 0.00; Chi² = 7.28, df = 7 (P = 0.40); I² = 4%								
Test for overall effect: Z = 0.00 (P = 1.00)								

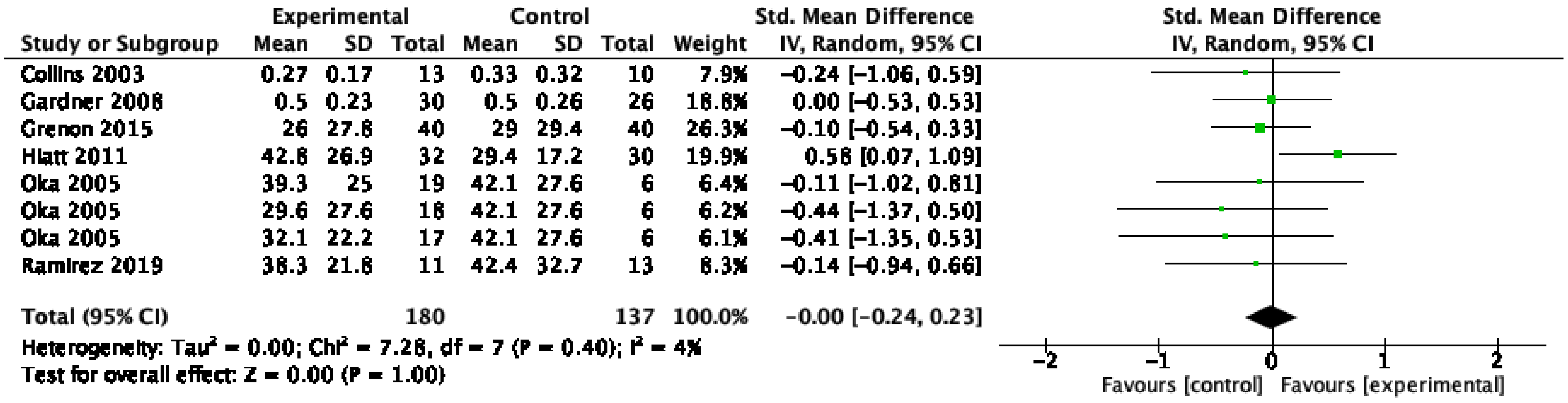

Figure S14. Funnel plot showing the impact of dietary supplements upregulating the NO pathway on quality of life (as measured by the WIQ walking speed domain).

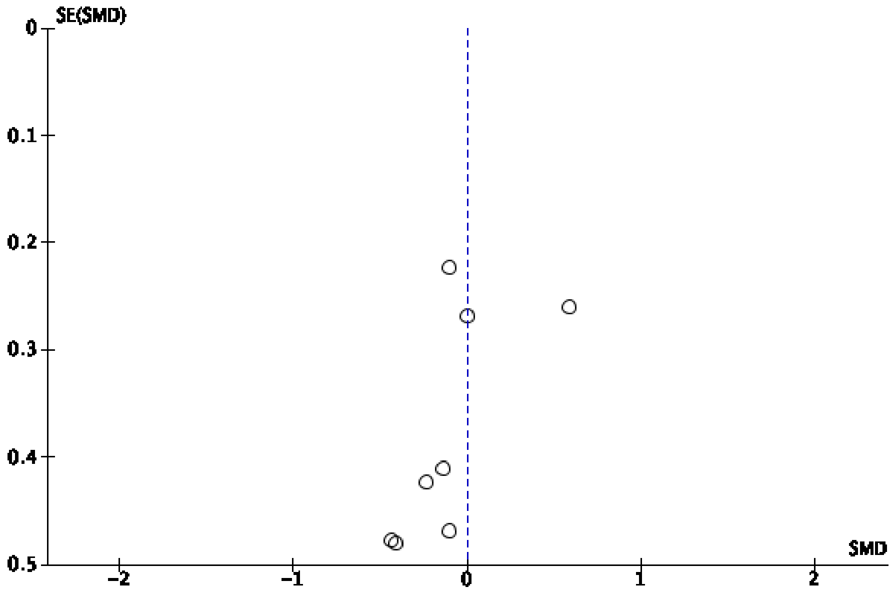

Figure S15. Forest plot showing effect of dietary supplements upregulating the NO pathway versus control on quality of life (as measured by the WIQ walking distance domain); studies stratified by risk of bias.

	Experimental			Control				Std. Mean Difference
Study or Subgroup	Mean	SD	Total	Mean	SD	Total	Weight	IV, Random, 95% CI
2.5.1 Low Risk of Bias								
Grenon 2015	27	36.3	40	29	29.4	40	25.2%	−0.06 [−0.50, 0.38]
Ramirez 2019	53	38.7	11	43	38.2	13	7.4%	0.25 [−0.56, 1.06]
Subtotal (95% CI)			51			53	32.6%	0.01 [-0.37, 0.40]
Heterogeneity: Tau² = 0.00; Chi² = 0.44, df = 1 (P = 0.51); I² = 0%								
Test for overall effect: Z = 0.06 (P = 0.96)								
2.5.2 Some Risk of Bias								
Collins 2003	0.26	0.28	13	0.36	0.34	10	7.0%	−0.31 [−1.14, 0.52]
Gardner 2008	0.52	0.32	30	0.54	0.33	26	17.5%	−0.06 [−0.59, 0.46]
Goldenberg 2012	35.4	29.4	68	33.8	27.1	68	42.8%	0.06 [−0.28, 0.39]
Subtotal (95% CI)			111			104	67.4%	-0.01 [-0.28, 0.26]
Heterogeneity: Tau² = 0.00; Chi² = 0.70, df = 2 (P = 0.71); I² = 0%								
Test for overall effect: Z = 0.09 (P = 0.93)								
2.5.3 High Risk of Bias								
Hiatt 2011	49.5	24.2	32	42.8	26.9	30		Not estimable
Subtotal (95% CI)			0			0		Not estimable
Heterogeneity: Not applicable								
Test for overall effect: Not applicable								
Total (95% CI)			162			157	100.0%	-0.01 [-0.22, 0.21]
Heterogeneity: Tau² = 0.00; Chi² = 1.15, df = 4 (P = 0.89); I² = 0%								
Test for overall effect: Z = 0.04 (P = 0.96)								
Test for subgroup differences: Chi² = 0.01, df = 1 (P = 0.92), I² = 0%								

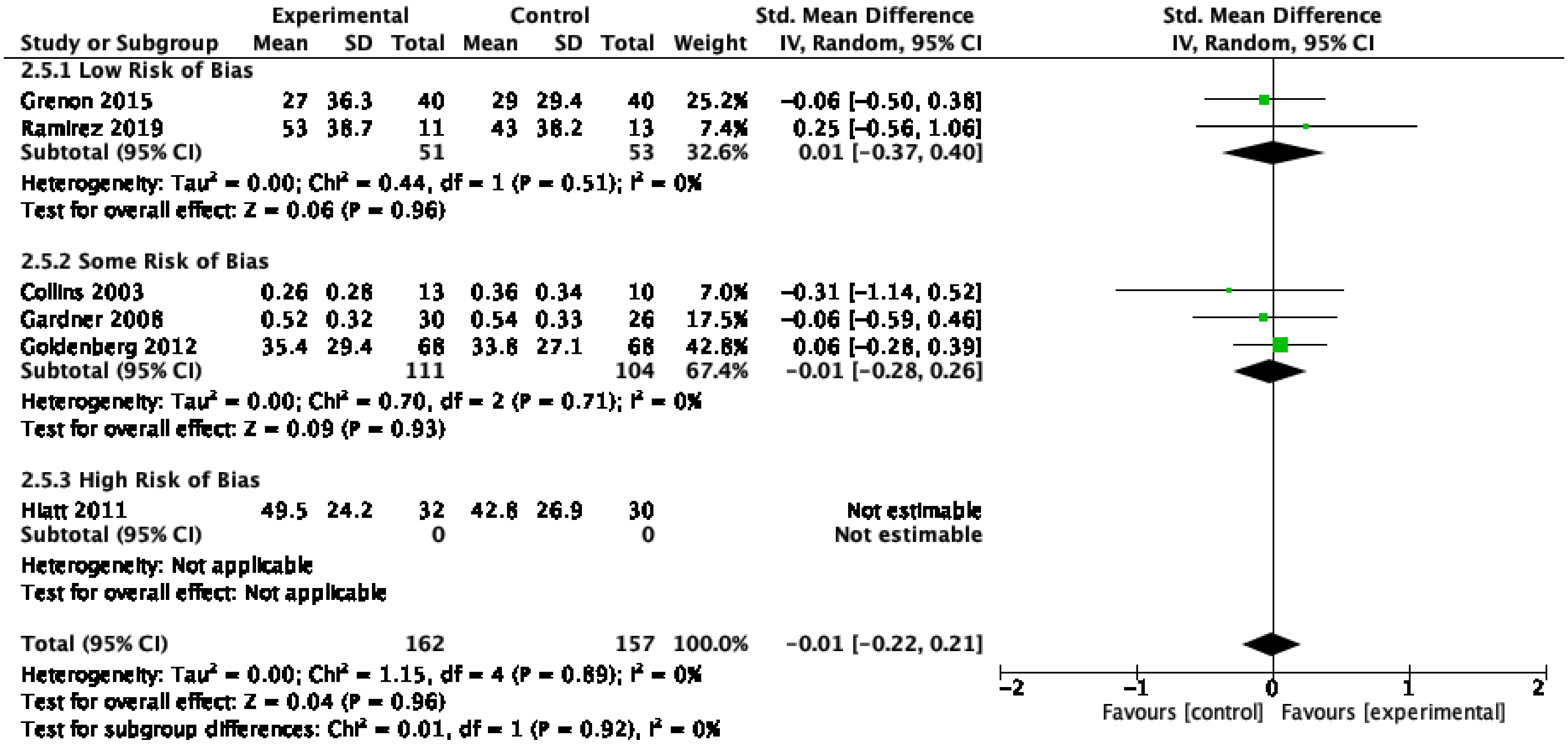

Figure S16. Forest plot showing effect of dietary supplements upregulating the NO pathway versus control on quality of life (as measured by the WIQ walking speed domain); studies stratified by risk of bias.

	Experimental			Control				Std. Mean Difference
**Study or Subgroup**	**Mean**	**SD**	**Total**	**Mean**	**SD**	**Total**	**Weight**	**IV, Random, 95% CI**
**2.8.1 Low Risk of Bias**								
Grenon 2015	26	27.8	40	29	29.4	40	34.0%	−0.10 [−0.54, 0.33]
Ramirez 2019	38.3	21.8	11	42.4	32.7	13	10.1%	−0.14 [−0.94, 0.66]
Subtotal (95% CI)			51			53	44.1%	−0.11 [−0.50, 0.27]
Heterogeneity: Tau² = 0.00; Chi² = 0.01, df = 1 (P = 0.94); I² = 0%								
Test for overall effect: Z = 0.57 (P = 0.57)								
**2.8.2 Some Risk of Bias**								
Collins 2003	0.27	0.17	13	0.33	0.32	10	9.5%	−0.24 [−1.06, 0.59]
Gardner 2008	0.5	0.23	30	0.5	0.26	26	23.7%	0.00 [−0.53, 0.53]
Oka 2005	39.3	25	19	42.1	27.6	6	7.8%	−0.11 [−1.02, 0.81]
Oka 2005	29.6	27.6	18	42.1	27.6	6	7.5%	−0.44 [−1.37, 0.50]
Oka 2005	32.1	22.2	17	42.1	27.6	6	7.4%	−0.41 [−1.35, 0.53]
Subtotal (95% CI)			97			54	55.9%	−0.17 [−0.51, 0.17]
Heterogeneity: Tau² = 0.00; Chi² = 1.01, df = 4 (P = 0.91); I² = 0%								
Test for overall effect: Z = 0.96 (P = 0.34)								
**2.8.3 High Risk of Bias**								
Hiatt 2011	42.8	26.9	32	29.4	17.2	30		Not estimable
Subtotal (95% CI)			0			0		Not estimable
Heterogeneity: Not applicable								
Test for overall effect: Not applicable								
**Total (95% CI)**			148			107	100.0%	−0.14 [−0.40, 0.11]
Heterogeneity: Tau² = 0.00; Chi² = 1.06, df = 6 (P = 0.98); I² = 0%								
Test for overall effect: Z = 1.10 (P = 0.27)								
Test for subgroup differences: Chi² = 0.04, df = 1 (P = 0.83), I² = 0%								

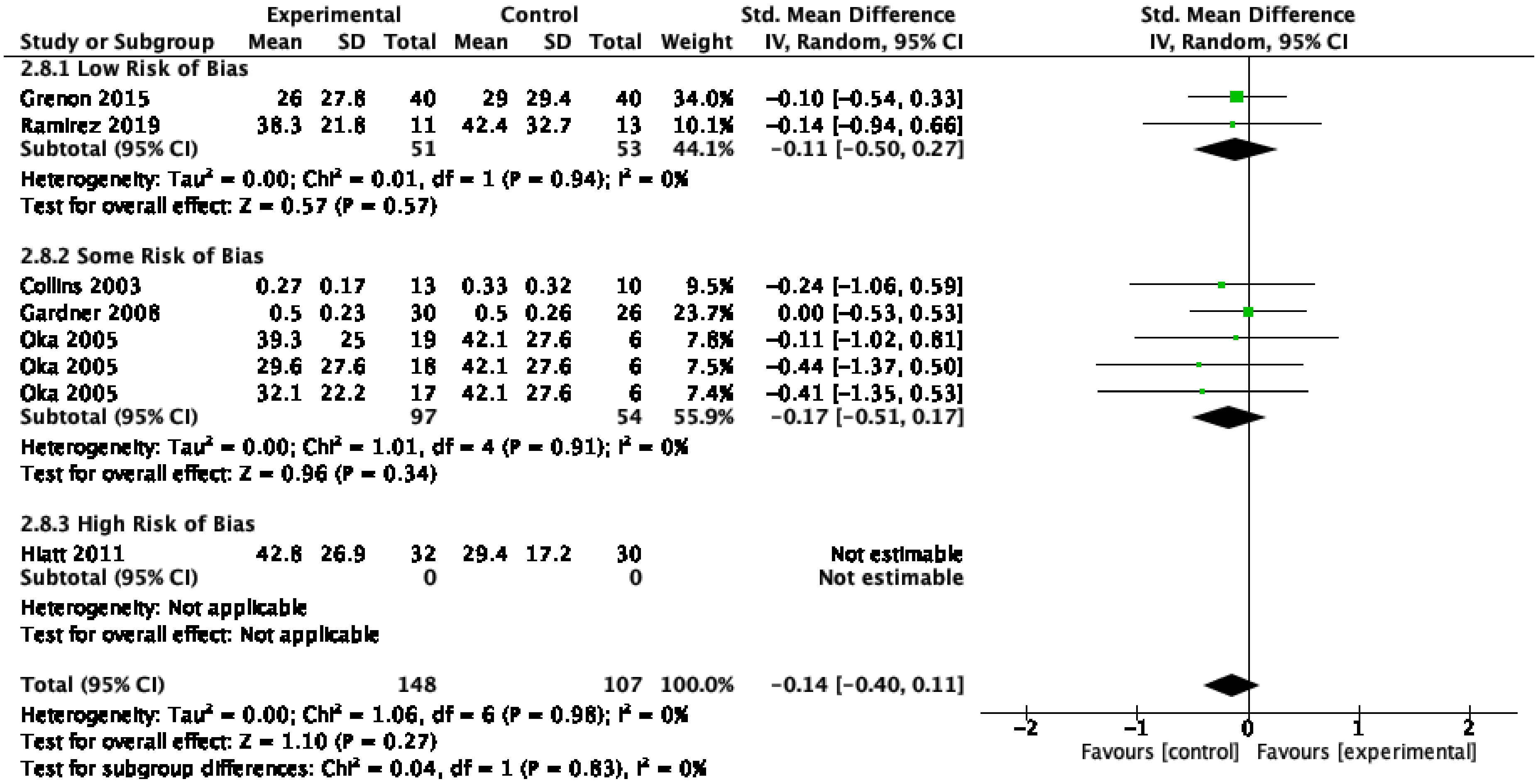

Figure S17. Forest plot showing effect of dietary supplements upregulating the NO pathway versus control on ABI.

	Experimental			Control				Std. Mean Difference
**Study or Subgroup**	**Mean**	**SD**	**Total**	**Mean**	**SD**	**Total**	**Weight**	**IV, Random, 95% CI**
Bock 2018	0.86	0.21	13	0.85	0.15	8	8.2%	0.05 [−0.83, 0.93]
Brevetti 1988	0.64	0.14	10	0.61	0.11	10	8.2%	0.23 [−0.65, 1.11]
Collins 2003	0.63	0.12	13	0.82	0.17	10	8.0%	−1.28 [−2.19, −0.36]
Dal Lago 1999	0.66	0.09	9	0.72	0.15	10	8.0%	−0.46 [−1.37, 0.46]
Grenon 2015	0.69	0.12	40	0.68	0.18	40	10.1%	0.06 [−0.37, 0.50]
Gresele 2012	0.67	0.16	221	0.66	0.14	221	10.8%	0.07 [−0.12, 0.25]
Luo 2013	0.73	0.22	120	0.71	0.18	119	10.6%	0.10 [−0.15, 0.35]
Ramirez 2019	0.66	0.09	11	0.71	0.12	13	8.5%	−0.45 [−1.26, 0.37]
Santo 2006	0.88	0.03	37	0.72	0.06	37	8.9%	3.34 [2.62, 4.05]
Wilson 2007	0.71	0.03	52	0.67	0.03	56	10.2%	1.32 [0.91, 1.74]
Woessner 2018	0.77	0.2	11	0.76	0.22	13	8.5%	0.05 [−0.76, 0.85]
**Total (95% CI)**			**537**			**537**	**100.0%**	**0.31 [−0.19, 0.81]**
Heterogeneity: Tau² = 0.59; Chi² = 118.63, df = 10 (P < 0.00001); I² = 92%								
Test for overall effect: Z = 1.21 (P = 0.23)								

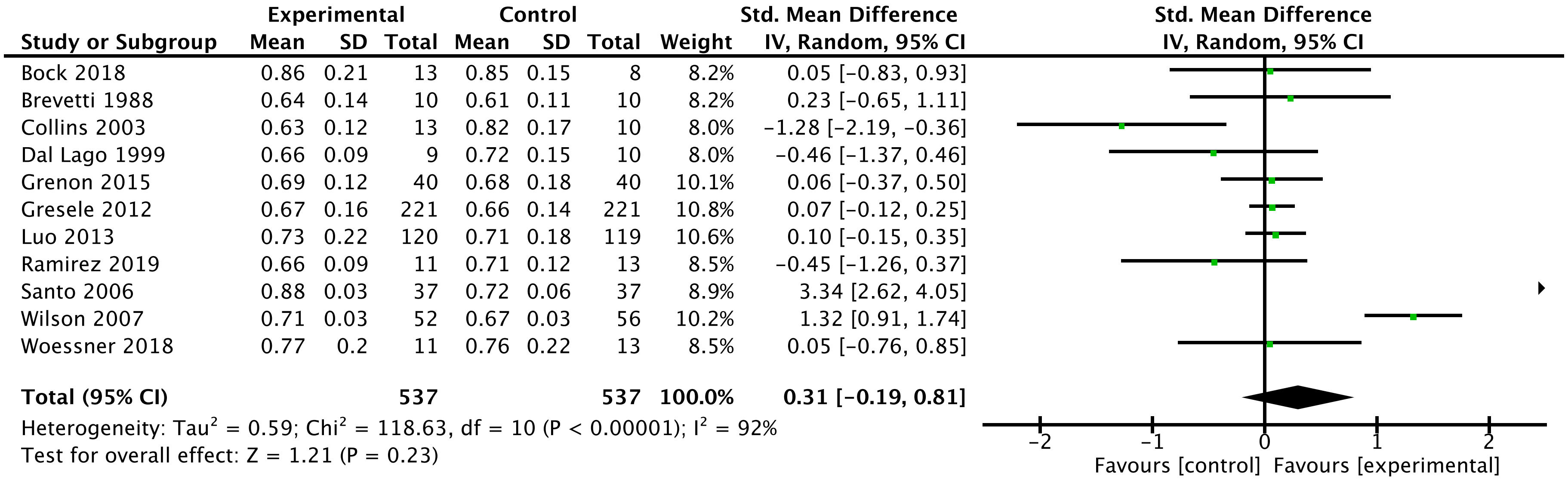

Figure S18. Funnel plot showing the impact of dietary supplements upregulating the NO pathway on ABI.

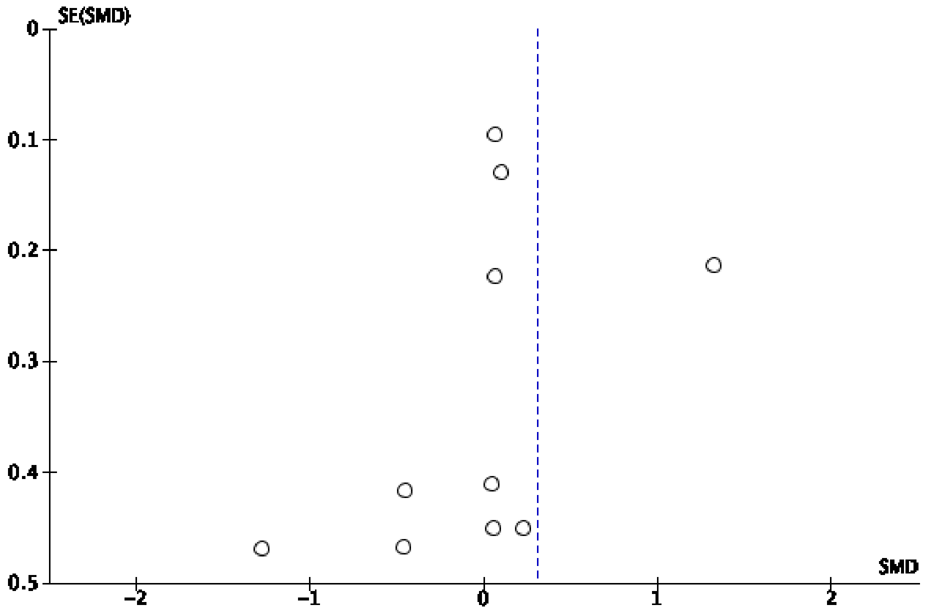

Figure S19. Forest plot showing effect of dietary supplements upregulating the NO pathway versus control on ABI; studies stratified by risk of bias.

	Experimental			Control				Std. Mean Difference
Study or Subgroup	Mean	SD	Total	Mean	SD	Total	Weight	IV, Random, 95% CI
**3.1.1 Low Risk of Bias**								
Bock 2018	0.86	0.21	13	0.85	0.15	8	8.2%	0.05 [−0.83, 0.93]
Grenon 2015	0.69	0.12	40	0.68	0.18	40	10.1%	0.06 [−0.37, 0.50]
Gresele 2012	0.67	0.16	221	0.66	0.14	221	10.8%	0.07 [−0.12, 0.25]
Ramirez 2019	0.66	0.09	11	0.71	0.12	13	8.5%	−0.45 [−1.26, 0.37]
Wilson 2007	0.71	0.03	52	0.67	0.03	56	10.2%	1.32 [0.91, 1.74]
**Subtotal (95% CI)**			**337**			**338**	**47.7%**	**0.25 [−0.32, 0.83]**
Heterogeneity: Tau^2 = 0.35; Chi^2 = 32.60, df = 4 (P < 0.00001); I^2 = 88%								
Test for overall effect: Z = 0.87 (P = 0.39)								
**3.1.2 Some Risk of Bias**								
Brevetti 1988	0.64	0.14	10	0.61	0.11	10	8.2%	0.23 [−0.65, 1.11]
Collins 2003	0.63	0.12	13	0.82	0.17	10	8.0%	−1.28 [−2.19, −0.36]
Dal Lago 1999	0.66	0.09	9	0.72	0.15	10	8.0%	−0.46 [−1.37, 0.46]
Luo 2013	0.73	0.22	120	0.71	0.18	119	10.6%	0.10 [−0.15, 0.35]
Santo 2006	0.88	0.03	37	0.72	0.06	37	8.9%	3.34 [2.62, 4.05]
Woessner 2018	0.77	0.2	11	0.76	0.22	13	8.5%	0.05 [−0.76, 0.85]
**Subtotal (95% CI)**			**200**			**199**	**52.3%**	**0.35 [−0.78, 1.47]**
Heterogeneity: Tau^2 = 1.81; Chi^2 = 85.81, df = 5 (P < 0.00001); I^2 = 94%								
Test for overall effect: Z = 0.60 (P = 0.55)								
**3.1.3 High Risk of Bias**								
**Subtotal (95% CI)**			**0**			**0**		**Not estimable**
Heterogeneity: Not applicable								
Test for overall effect: Not applicable								
**Total (95% CI)**			**537**			**537**	**100.0%**	**0.31 [−0.19, 0.81]**
Heterogeneity: Tau^2 = 0.59; Chi^2 = 118.63, df = 10 (P < 0.00001); I^2 = 92%								
Test for overall effect: Z = 1.21 (P = 0.23)								
Test for subgroup differences: Chi^2 = 0.02, df = 1 (P = 0.89), I^2 = 0%								

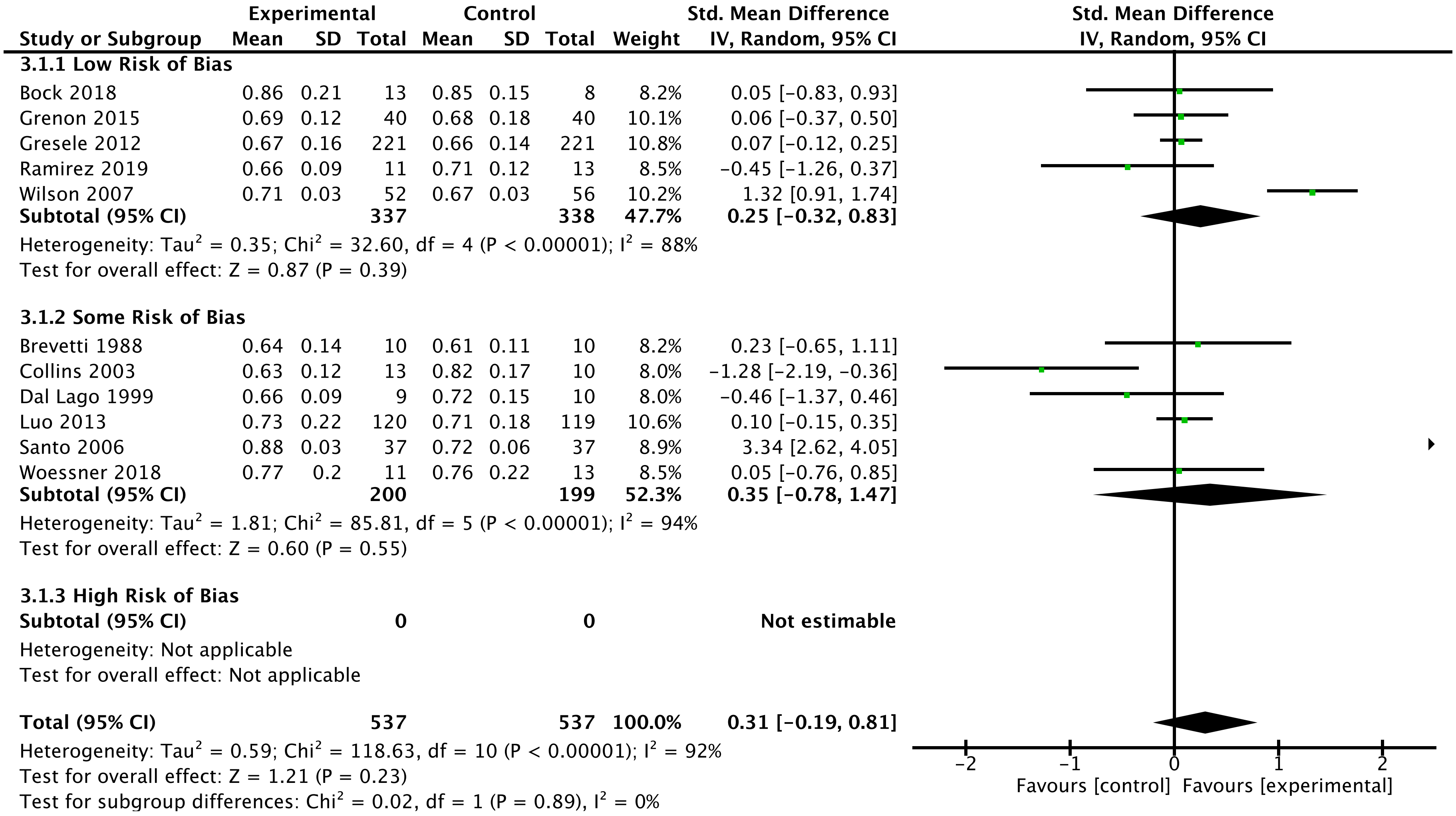

Figure S20. Forest plot showing effect of dietary supplements upregulating the NO pathway versus control on ABI; studies stratified by intervention type.

Study or Subgroup	Experimental Mean	Experimental SD	Experimental Total	Control Mean	Control SD	Control Total	Weight	Std. Mean Difference IV, Random, 95% CI
**3.3.1 Nitric oxide donors**								
Bock 2018	0.86	0.21	13	0.85	0.15	8	9.0%	0.05 [−0.83, 0.93]
Gresele 2012	0.67	0.16	221	0.66	0.14	221	12.3%	0.07 [−0.12, 0.25]
Woessner 2018	0.77	0.2	11	0.76	0.22	13	9.4%	0.05 [−0.76, 0.85]
Subtotal (95% CI)			245			242	30.7%	0.06 [−0.11, 0.24]
Heterogeneity: Tau² = 0.00; Chi² = 0.00, df = 2 (P = 1.00); I² = 0%								
Test for overall effect: Z = 0.71 (P = 0.48)								
**3.3.2 Antioxidants**								
Brevetti 1988	0.64	0.14	10	0.61	0.11	10	9.0%	0.23 [−0.65, 1.11]
Collins 2003	0.63	0.12	13	0.82	0.17	10	8.8%	−1.28 [−2.19, −0.36]
Dal Lago 1999	0.66	0.09	9	0.72	0.15	10	8.8%	−0.46 [−1.37, 0.46]
Grenon 2015	0.69	0.12	40	0.68	0.18	40	11.4%	0.06 [−0.37, 0.50]
Luo 2013	0.73	0.22	120	0.71	0.18	119	12.1%	0.10 [−0.15, 0.35]
Ramirez 2019	0.66	0.09	11	0.71	0.12	13	9.4%	−0.45 [−1.26, 0.37]
Santo 2006	0.88	0.03	37	0.72	0.06	37	9.9%	3.34 [2.62, 4.05]
Subtotal (95% CI)			240			239	69.3%	0.24 [−0.62, 1.10]
Heterogeneity: Tau² = 1.21; Chi² = 89.01, df = 6 (P < 0.00001); I² = 93%								
Test for overall effect: Z = 0.54 (P = 0.59)								
**3.3.3 Enhancers of NO availability**								
Wilson 2007	0.71	0.03	52	0.67	0.03	56		Not estimable
Subtotal (95% CI)			0			0		Not estimable
Heterogeneity: Not applicable								
Test for overall effect: Not applicable								
**3.3.4 Nitric oxide synthase inducers**								
Subtotal (95% CI)			0			0		Not estimable
Heterogeneity: Not applicable								
Test for overall effect: Not applicable								
**Total (95% CI)**			485			481	100.0%	0.19 [−0.30, 0.69]
Heterogeneity: Tau² = 0.52; Chi² = 90.27, df = 9 (P < 0.00001); I² = 90%								
Test for overall effect: Z = 0.76 (P = 0.45)								
Test for subgroup differences: Chi² = 0.15, df = 1 (P = 0.70), I² = 0%								

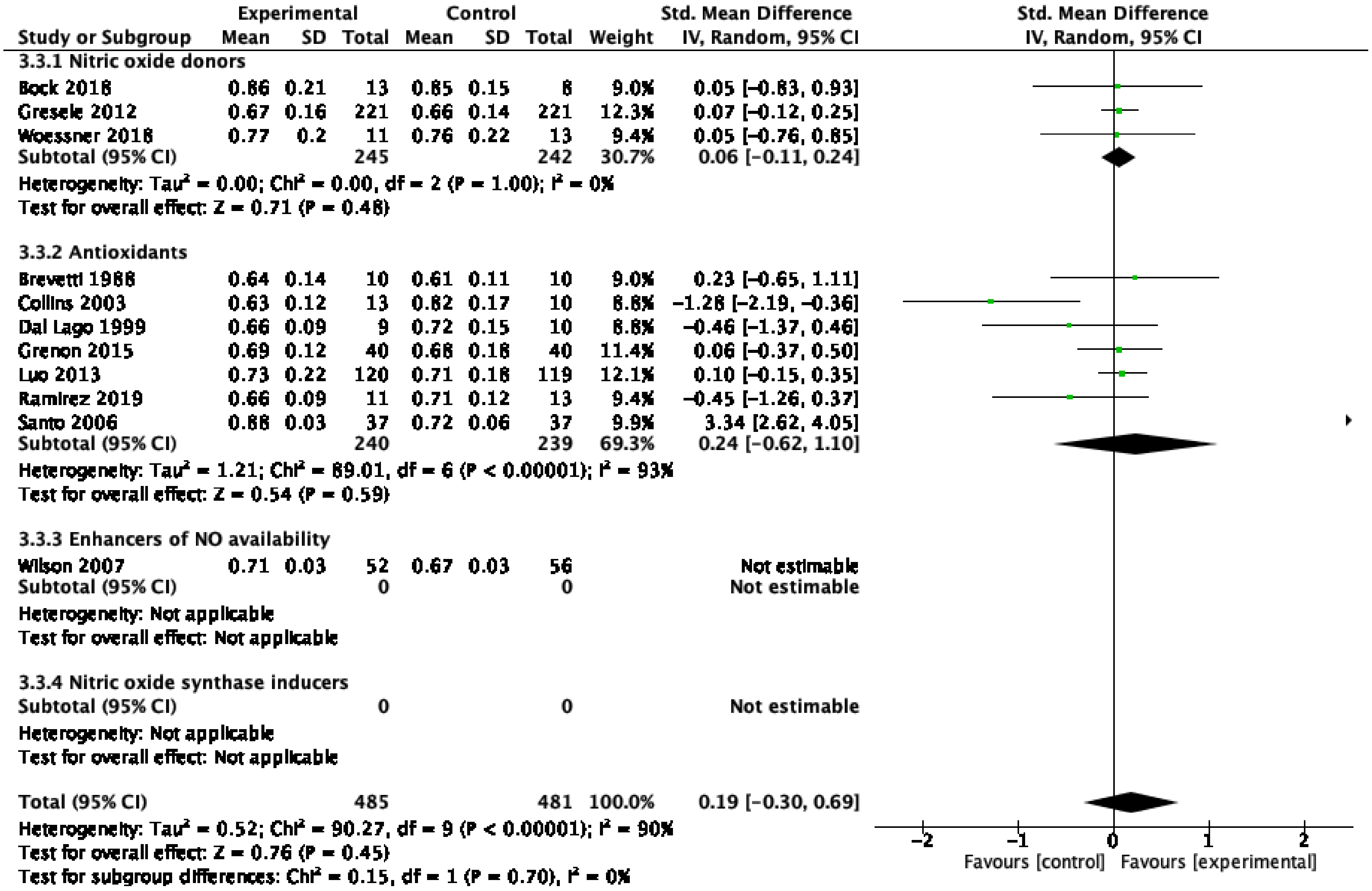

Figure S21. Forest plot showing effect of dietary supplements upregulating the NO pathway versus control on risk of adverse events.

Study or Subgroup	Experimental Events	Experimental Total	Control Events	Control Total	Weight	Odds Ratio M-H, Random, 95% CI
Bock 2018	0	13	0	8		Not estimable
Brevetti 1995	11	99	3	115	7.4%	4.67 [1.26, 17.24]
Brevetti 1999	38	239	98	246	11.8%	0.29 [0.19, 0.44]
Coto 1992	3	142	5	140	6.7%	0.58 [0.14, 2.49]
Gardner 2008	2	31	2	31	4.7%	1.00 [0.13, 7.59]
Goldenberg 2012	35	80	39	83	11.0%	0.88 [0.47, 1.63]
Gresele 2012	90	221	69	221	12.0%	1.51 [1.02, 2.24]
Hiatt 2011	24	34	24	35	8.8%	1.10 [0.39, 3.07]
Luo 2013	36	120	30	119	11.2%	1.27 [0.72, 2.25]
Maxwell 2000	2	27	1	13	3.5%	0.96 [0.08, 11.66]
Mohler 2014	26	37	9	18	8.1%	2.36 [0.74, 7.56]
van der Avoort 2021	0	6	0	3		Not estimable
van der Avoort 2021	0	6	0	3		Not estimable
Vincent 2007	7	16	0	12	2.7%	19.74 [1.00, 390.30]
Wilson 2007	55	66	44	67	9.9%	2.61 [1.15, 5.94]
Woessner 2018	1	11	0	13	2.3%	3.86 [0.14, 104.65]
**Total (95% CI)**		**1148**		**1127**	**100.0%**	**1.32 [0.76, 2.28]**
Total events	330		324			
Heterogeneity: Tau² = 0.62; Chi² = 55.46, df = 12 (P < 0.00001); I² = 78%						
Test for overall effect: Z = 0.98 (P = 0.33)						

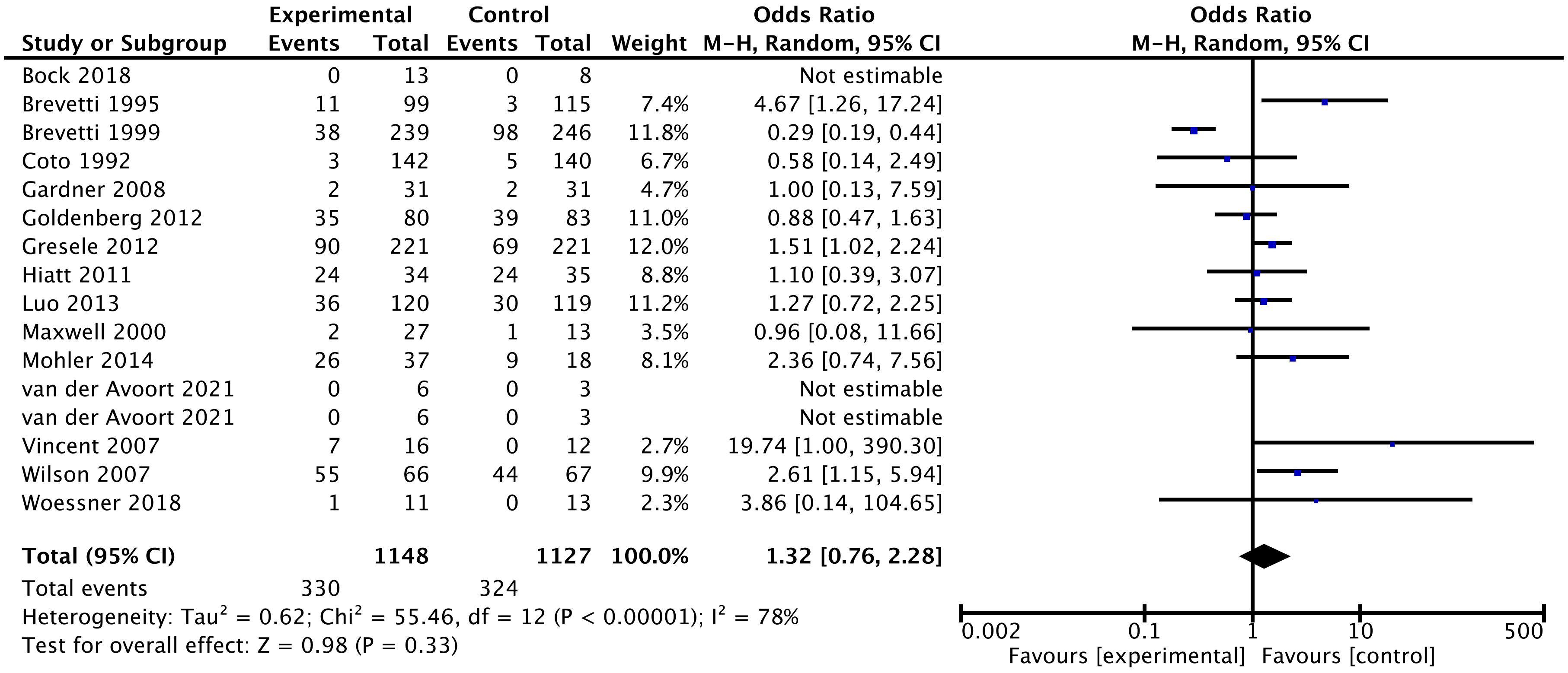

Figure S22. Funnel plot showing the impact of dietary supplements upregulating the NO pathway on risk of adverse events.

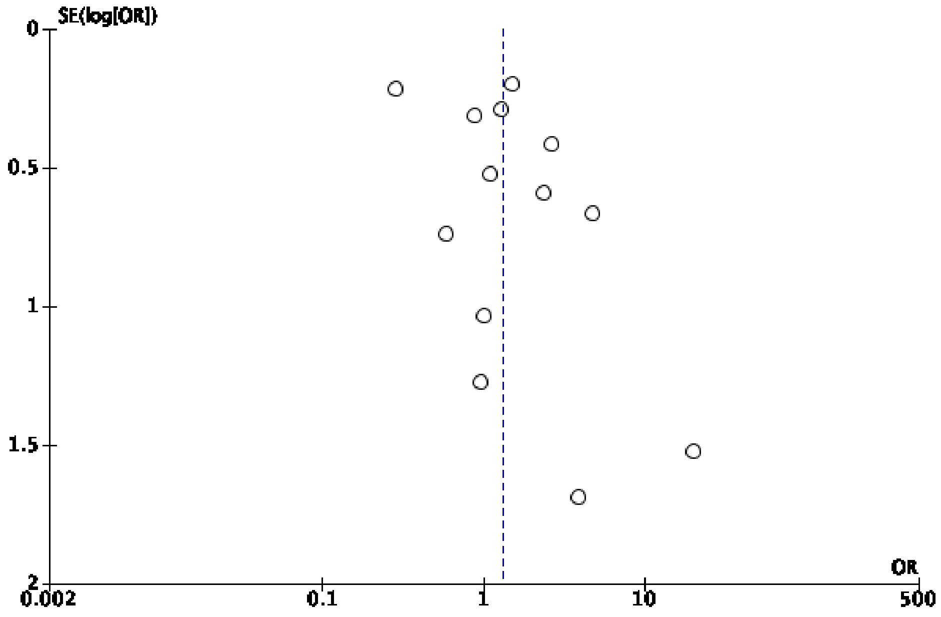

Figure S23. Forest plot showing effect of dietary supplements upregulating the NO pathway versus control on risk of adverse events; studies stratified by risk of bias.

Study or Subgroup	Experimental Events	Experimental Total	Control Events	Control Total	Weight	Odds Ratio M-H, Random, 95% CI
**4.2.1 Low Risk of Bias**						
Bock 2018	0	13	0	8		Not estimable
Gresele 2012	90	221	69	221	12.0%	1.51 [1.02, 2.24]
van der Avoort 2021	0	6	0	3		Not estimable
van der Avoort 2021	0	6	0	3		Not estimable
Wilson 2007	55	66	44	67	9.9%	2.61 [1.15, 5.94]
**Subtotal (95% CI)**		**312**		**302**	**21.9%**	**1.76 [1.09, 2.83]**
Total events	145		113			
Heterogeneity: $Tau^2 = 0.04$; $Chi^2 = 1.39$, df = 1 (P = 0.24); $I^2 = 28\%$						
Test for overall effect: Z = 2.31 (P = 0.02)						
**4.2.2 Some Risk of Bias**						
Coto 1992	3	142	5	140	6.7%	0.58 [0.14, 2.49]
Gardner 2008	2	31	2	31	4.7%	1.00 [0.13, 7.59]
Goldenberg 2012	35	80	39	83	11.0%	0.88 [0.47, 1.63]
Luo 2013	36	120	30	119	11.2%	1.27 [0.72, 2.25]
Maxwell 2000	2	27	1	13	3.5%	0.96 [0.08, 11.66]
Mohler 2014	26	37	9	18	8.1%	2.36 [0.74, 7.56]
Vincent 2007	7	16	0	12	2.7%	19.74 [1.00, 390.30]
Woessner 2018	1	11	0	13	2.3%	3.86 [0.14, 104.65]
**Subtotal (95% CI)**		**464**		**429**	**50.1%**	**1.19 [0.81, 1.74]**
Total events	112		86			
Heterogeneity: $Tau^2 = 0.01$; $Chi^2 = 7.27$, df = 7 (P = 0.40); $I^2 = 4\%$						
Test for overall effect: Z = 0.87 (P = 0.39)						
**4.2.3 High Risk of Bias**						
Brevetti 1995	11	99	3	115	7.4%	4.67 [1.26, 17.24]
Brevetti 1999	38	239	98	246	11.8%	0.29 [0.19, 0.44]
Hiatt 2011	24	34	24	35	8.8%	1.10 [0.39, 3.07]
**Subtotal (95% CI)**		**372**		**396**	**28.0%**	**1.04 [0.21, 5.22]**
Total events	73		125			
Heterogeneity: $Tau^2 = 1.80$; $Chi^2 = 19.62$, df = 2 (P < 0.0001); $I^2 = 90\%$						
Test for overall effect: Z = 0.04 (P = 0.96)						
**Total (95% CI)**		**1148**		**1127**	**100.0%**	**1.32 [0.76, 2.28]**
Total events	330		324			
Heterogeneity: $Tau^2 = 0.62$; $Chi^2 = 55.46$, df = 12 (P < 0.00001); $I^2 = 78\%$						
Test for overall effect: Z = 0.98 (P = 0.33)						
Test for subgroup differences: $Chi^2 = 1.70$, df = 2 (P = 0.43), $I^2 = 0\%$						

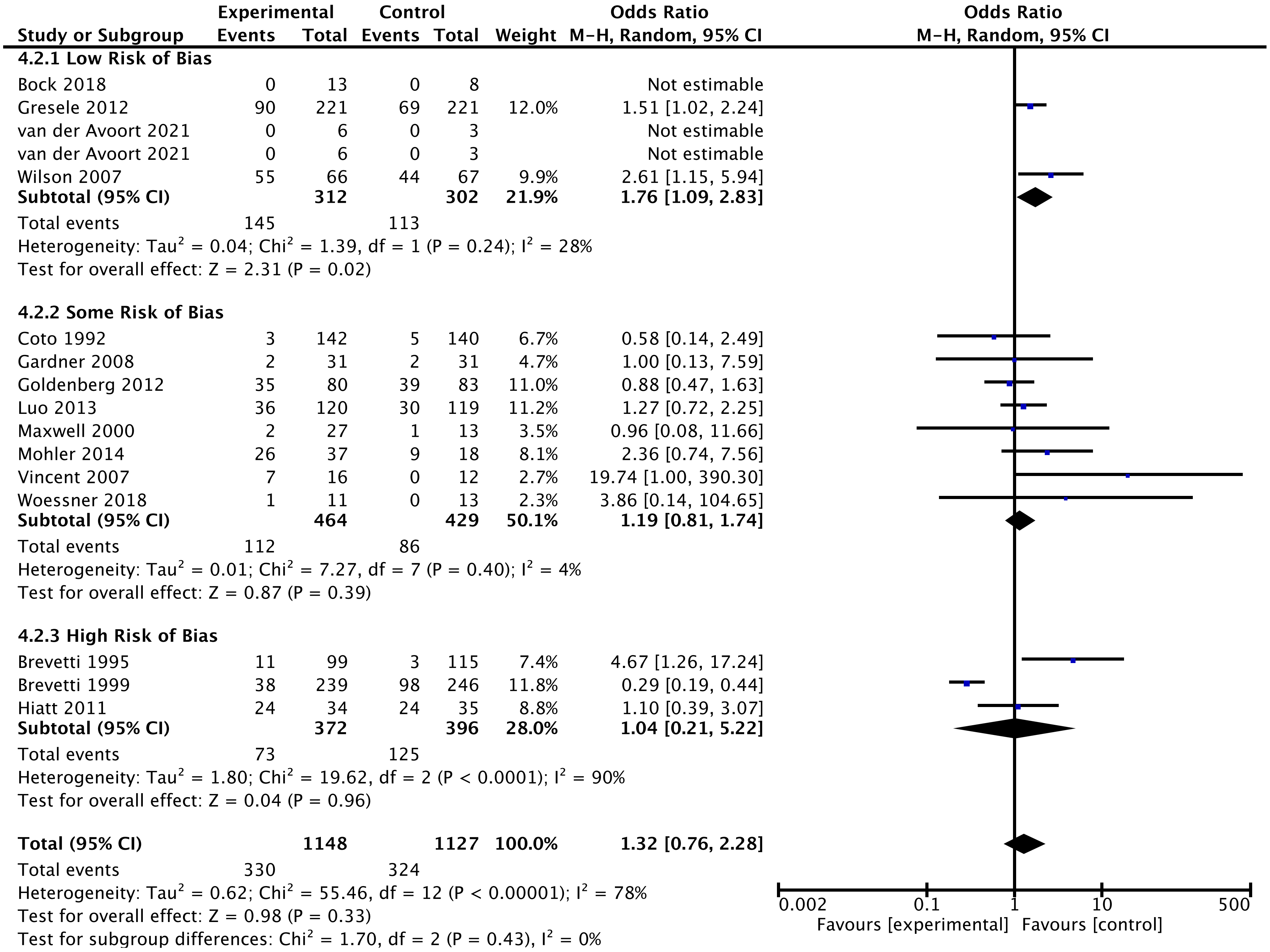

Figure S24. Forest plot showing effect of dietary supplements upregulating the NO pathway versus control on risk of adverse events; studies stratified by type of intervention.

Study or Subgroup	Experimental Events	Experimental Total	Control Events	Control Total	Weight	Odds Ratio M-H, Random, 95% CI
**4.3.1 Nitric oxide donors**						
Bock 2018	0	13	0	8		Not estimable
Gresele 2012	90	221	69	221	12.0%	1.51 [1.02, 2.24]
Mohler 2014	26	37	9	18	8.1%	2.36 [0.74, 7.56]
van der Avoort 2021	0	6	0	3		Not estimable
van der Avoort 2021	0	6	0	3		Not estimable
Woessner 2018	1	11	0	13	2.3%	3.86 [0.14, 104.65]
**Subtotal (95% CI)**		**294**		**266**	**22.3%**	**1.60 [1.11, 2.31]**
Total events	117		78			
Heterogeneity: $Tau^2 = 0.00$; $Chi^2 = 0.78$, df = 2 (P = 0.68); $I^2 = 0\%$						
Test for overall effect: Z = 2.51 (P = 0.01)						
**4.3.2 Antioxidants**						
Brevetti 1995	11	99	3	115	7.4%	4.67 [1.26, 17.24]
Brevetti 1999	38	239	98	246	11.8%	0.29 [0.19, 0.44]
Coto 1992	3	142	5	140	6.7%	0.58 [0.14, 2.49]
Gardner 2008	2	31	2	31	4.7%	1.00 [0.13, 7.59]
Goldenberg 2012	35	80	39	83	11.0%	0.88 [0.47, 1.63]
Hiatt 2011	24	34	24	35	8.8%	1.10 [0.39, 3.07]
Luo 2013	36	120	30	119	11.2%	1.27 [0.72, 2.25]
Vincent 2007	7	16	0	12	2.7%	19.74 [1.00, 390.30]
**Subtotal (95% CI)**		**761**		**781**	**64.2%**	**1.07 [0.52, 2.23]**
Total events	156		201			
Heterogeneity: $Tau^2 = 0.74$; $Chi^2 = 35.27$, df = 7 (P < 0.00001); $I^2 = 80\%$						
Test for overall effect: Z = 0.18 (P = 0.85)						
**4.3.3 Enhancers of NO availability**						
Maxwell 2000	2	27	1	13	3.5%	0.96 [0.08, 11.66]
Wilson 2007	55	66	44	67	9.9%	2.61 [1.15, 5.94]
**Subtotal (95% CI)**		**93**		**80**	**13.4%**	**2.37 [1.09, 5.17]**
Total events	57		45			
Heterogeneity: $Tau^2 = 0.00$; $Chi^2 = 0.56$, df = 1 (P = 0.46); $I^2 = 0\%$						
Test for overall effect: Z = 2.17 (P = 0.03)						
**4.3.4 Nitric oxide synthase inducers**						
**Subtotal (95% CI)**		**0**		**0**		**Not estimable**
Total events	0		0			
Heterogeneity: Not applicable						
Test for overall effect: Not applicable						
**Total (95% CI)**		**1148**		**1127**	**100.0%**	**1.32 [0.76, 2.28]**
Total events	330		324			
Heterogeneity: $Tau^2 = 0.62$; $Chi^2 = 55.46$, df = 12 (P < 0.00001); $I^2 = 78\%$						
Test for overall effect: Z = 0.98 (P = 0.33)						
Test for subgroup differences: $Chi^2 = 2.13$, df = 2 (P = 0.34), $I^2 = 6.1\%$						

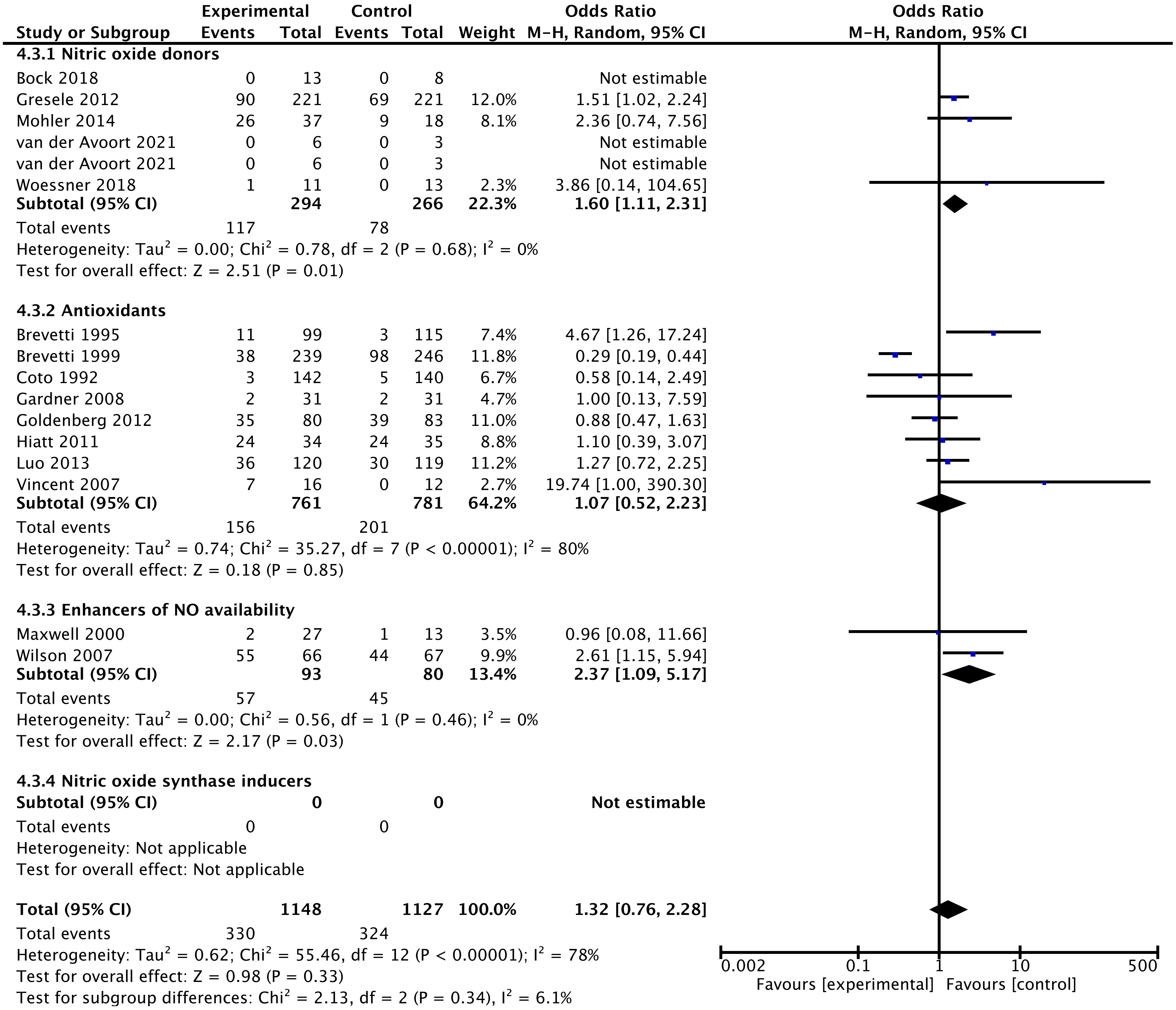

Figure S25. Forest plot showing effect of dietary supplements upregulating the NO pathway versus control on risk of serious adverse events.

Study or Subgroup	Experimental Events	Experimental Total	Control Events	Control Total	Weight	Odds Ratio M-H, Random, 95% CI
Bock 2018	0	13	0	8		Not estimable
Brevetti 1999	27	239	30	246	34.8%	0.92 [0.53, 1.59]
Collins 2003	1	13	2	12	1.6%	0.42 [0.03, 5.30]
Coto 1992	0	142	1	140	1.0%	0.33 [0.01, 8.08]
Goldenberg 2012	12	80	19	83	16.7%	0.59 [0.27, 1.32]
Gresele 2012	6	221	4	221	6.5%	1.51 [0.42, 5.44]
Hiatt 2011	4	34	6	35	5.7%	0.64 [0.16, 2.52]
Kiesewetter 1993	0	32	0	32		Not estimable
Leng 1997	12	55	16	65	14.6%	0.85 [0.36, 2.01]
Luo 2013	2	120	4	119	3.6%	0.49 [0.09, 2.71]
Maxwell 2000	0	27	0	13		Not estimable
McDermott 2020	5	23	2	21	3.4%	2.64 [0.45, 15.37]
Mohler 2014	0	37	2	18	1.1%	0.09 [0.00, 1.94]
van der Avoort 2021	0	6	0	3		Not estimable
van der Avoort 2021	0	6	0	3		Not estimable
Wilson 2007	9	66	9	67	10.8%	1.02 [0.38, 2.75]
Woessner 2018	0	12	0	14		Not estimable
**Total (95% CI)**		**1126**		**1100**	**100.0%**	**0.83 [0.60, 1.16]**
Total events	78		95			
Heterogeneity: $Tau^2 = 0.00$; $Chi^2 = 6.60$, df = 10 (P = 0.76); $I^2 = 0\%$						
Test for overall effect: Z = 1.09 (P = 0.27)						

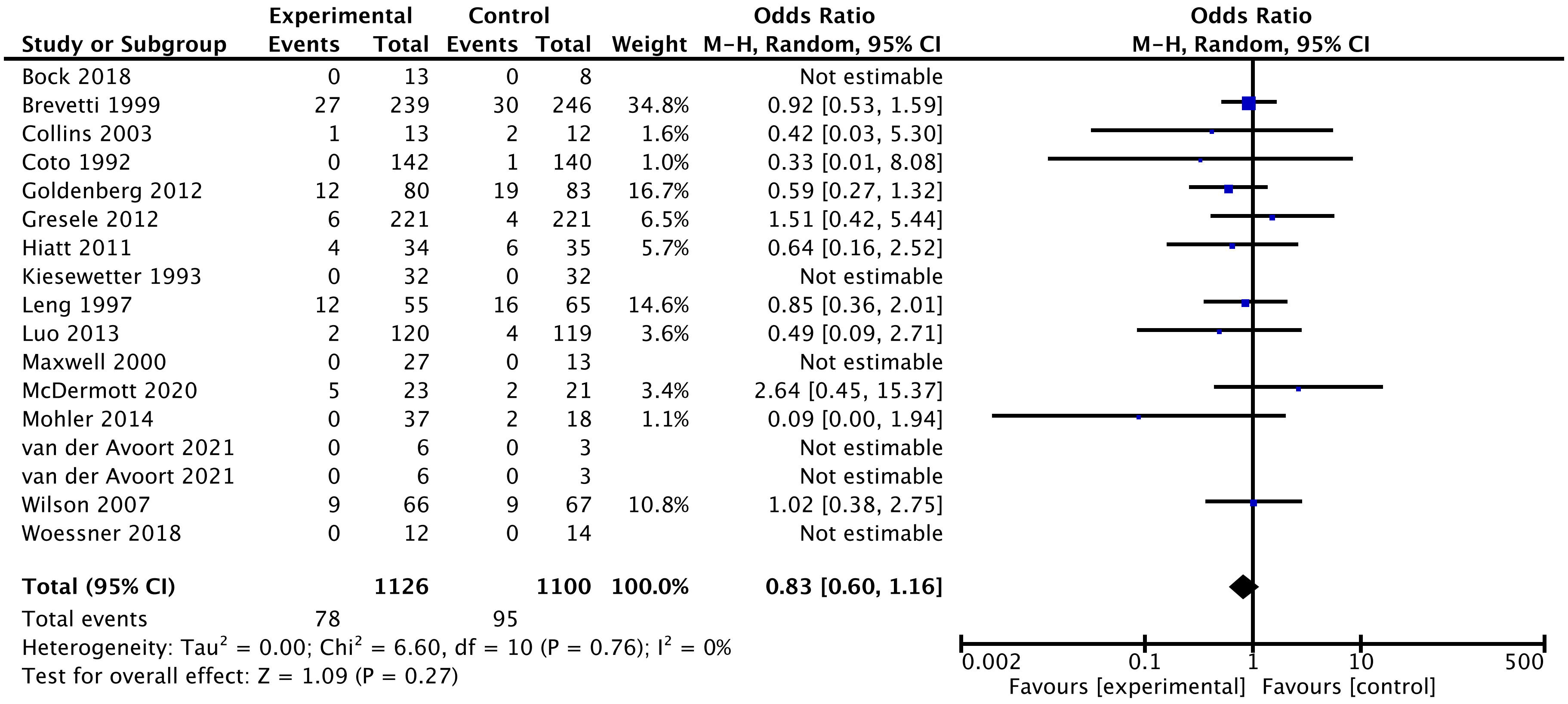

Figure S26. Funnel plot showing the impact of dietary supplements upregulating the NO pathway on risk of serious adverse events.

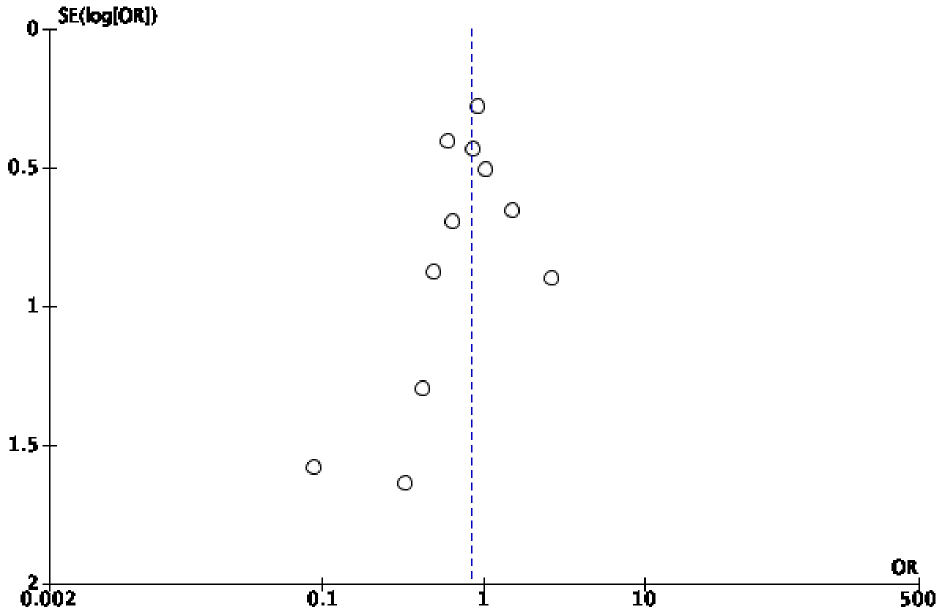

Figure S27. Forest plot showing effect of dietary supplements upregulating the NO pathway versus control on risk of serious adverse events; studies stratified by risk of bias.

Study or Subgroup	Experimental Events	Experimental Total	Control Events	Control Total	Weight	Odds Ratio M-H, Random, 95% CI
**4.3.1 Low Risk of Bias**						
Bock 2018	0	13	0	8		Not estimable
Gresele 2012	6	221	4	221	6.5%	1.51 [0.42, 5.44]
Leng 1997	12	55	16	65	14.6%	0.85 [0.36, 2.01]
McDermott 2020	5	23	2	21	3.4%	2.64 [0.45, 15.37]
van der Avoort 2021	0	6	0	3		Not estimable
van der Avoort 2021	0	6	0	3		Not estimable
Wilson 2007	9	66	9	67	10.8%	1.02 [0.38, 2.75]
**Subtotal (95% CI)**		**390**		**388**	**35.4%**	**1.12 [0.65, 1.93]**
Total events	32		31			
Heterogeneity: Tau^2 = 0.00; Chi^2 = 1.54, df = 3 (P = 0.67); I^2 = 0%						
Test for overall effect: Z = 0.40 (P = 0.69)						
**4.3.2 Some Risk of Bias**						
Collins 2003	1	13	2	12	1.6%	0.42 [0.03, 5.30]
Coto 1992	0	142	1	140	1.0%	0.33 [0.01, 8.08]
Goldenberg 2012	12	80	19	83	16.7%	0.59 [0.27, 1.32]
Kiesewetter 1993	0	32	0	32		Not estimable
Luo 2013	2	120	4	119	3.6%	0.49 [0.09, 2.71]
Maxwell 2000	0	27	0	13		Not estimable
Mohler 2014	0	37	2	18	1.1%	0.09 [0.00, 1.94]
Woessner 2018	0	12	0	14		Not estimable
**Subtotal (95% CI)**		**463**		**431**	**24.1%**	**0.50 [0.26, 0.98]**
Total events	15		28			
Heterogeneity: Tau^2 = 0.00; Chi^2 = 1.49, df = 4 (P = 0.83); I^2 = 0%						
Test for overall effect: Z = 2.03 (P = 0.04)						
**4.3.3 High Risk of Bias**						
Brevetti 1999	27	239	30	246	34.8%	0.92 [0.53, 1.59]
Hiatt 2011	4	34	6	35	5.7%	0.64 [0.16, 2.52]
**Subtotal (95% CI)**		**273**		**281**	**40.5%**	**0.87 [0.52, 1.46]**
Total events	31		36			
Heterogeneity: Tau^2 = 0.00; Chi^2 = 0.22, df = 1 (P = 0.64); I^2 = 0%						
Test for overall effect: Z = 0.52 (P = 0.60)						
**Total (95% CI)**		**1126**		**1100**	**100.0%**	**0.83 [0.60, 1.16]**
Total events	78		95			
Heterogeneity: Tau^2 = 0.00; Chi^2 = 6.60, df = 10 (P = 0.76); I^2 = 0%						
Test for overall effect: Z = 1.09 (P = 0.27)						
Test for subgroup differences: Chi^2 = 3.35, df = 2 (P = 0.19), I^2 = 40.3%						

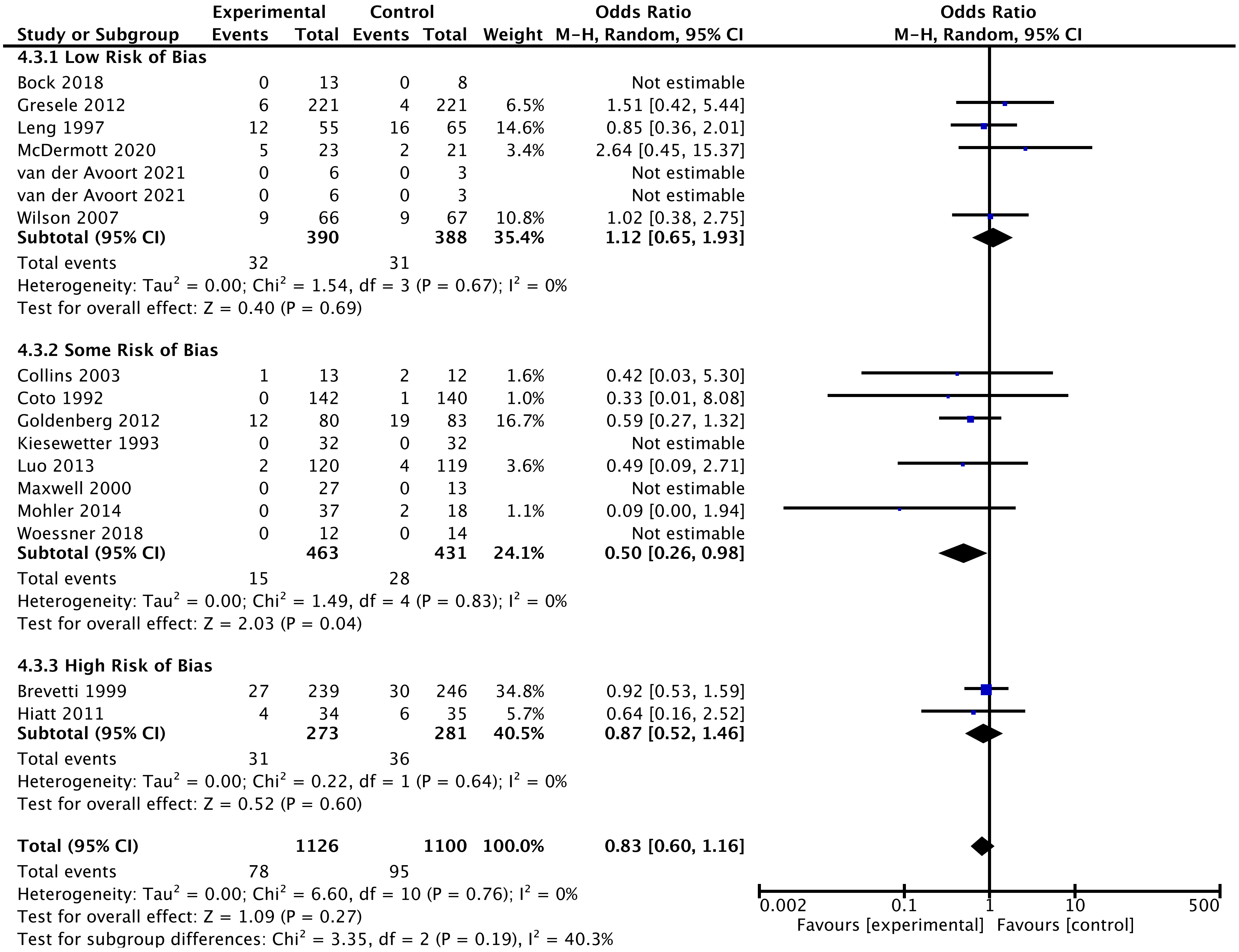

Figure S28. Forest plot showing effect of dietary supplements upregulating the NO pathway versus control on risk of serious adverse events; studies stratified by type of intervention.

Study or Subgroup	Experimental Events	Experimental Total	Control Events	Control Total	Weight	Odds Ratio M-H, Random, 95% CI
**4.6.1 Nitric oxide donors**						
Bock 2018	0	13	0	8		Not estimable
Gresele 2012	6	221	4	221	6.7%	1.51 [0.42, 5.44]
Mohler 2014	0	37	2	18	1.2%	0.09 [0.00, 1.94]
van der Avoort 2021	0	6	0	3		Not estimable
van der Avoort 2021	0	6	0	3		Not estimable
Woessner 2018	0	12	0	14		Not estimable
**Subtotal (95% CI)**		**295**		**267**	**7.9%**	**0.52 [0.03, 7.97]**
Total events	6		6			
Heterogeneity: $Tau^2 = 2.68$; $Chi^2 = 2.84$, df = 1 (P = 0.09); $I^2 = 65\%$						
Test for overall effect: Z = 0.47 (P = 0.64)						
**4.6.2 Antioxidants**						
Brevetti 1999	27	239	30	246	36.0%	0.92 [0.53, 1.59]
Collins 2003	1	13	2	12	1.7%	0.42 [0.03, 5.30]
Coto 1992	0	142	1	140	1.1%	0.33 [0.01, 8.08]
Goldenberg 2012	12	80	19	83	17.3%	0.59 [0.27, 1.32]
Hiatt 2011	4	34	6	35	5.9%	0.64 [0.16, 2.52]
Kiesewetter 1993	0	32	0	32		Not estimable
Leng 1997	12	55	16	65	15.2%	0.85 [0.36, 2.01]
Luo 2013	2	120	4	119	3.7%	0.49 [0.09, 2.71]
**Subtotal (95% CI)**		**715**		**732**	**80.9%**	**0.76 [0.52, 1.10]**
Total events	58		78			
Heterogeneity: $Tau^2 = 0.00$; $Chi^2 = 1.67$, df = 6 (P = 0.95); $I^2 = 0\%$						
Test for overall effect: Z = 1.47 (P = 0.14)						
**4.6.3 Enhancers of NO availability**						
Maxwell 2000	0	27	0	13		Not estimable
Wilson 2007	9	66	9	67	11.2%	1.02 [0.38, 2.75]
**Subtotal (95% CI)**		**93**		**80**	**11.2%**	**1.02 [0.38, 2.75]**
Total events	9		9			
Heterogeneity: Not applicable						
Test for overall effect: Z = 0.03 (P = 0.97)						
**4.6.4 Nitric oxide synthase inducers**						
McDermott 2020	5	23	2	21		Not estimable
**Subtotal (95% CI)**		**0**		**0**		**Not estimable**
Total events	0		0			
Heterogeneity: Not applicable						
Test for overall effect: Not applicable						
**Total (95% CI)**		**1103**		**1079**	**100.0%**	**0.80 [0.57, 1.12]**
Total events	73		93			
Heterogeneity: $Tau^2 = 0.00$; $Chi^2 = 4.90$, df = 9 (P = 0.84); $I^2 = 0\%$						
Test for overall effect: Z = 1.32 (P = 0.19)						
Test for subgroup differences: $Chi^2 = 0.38$, df = 2 (P = 0.83), $I^2 = 0\%$						

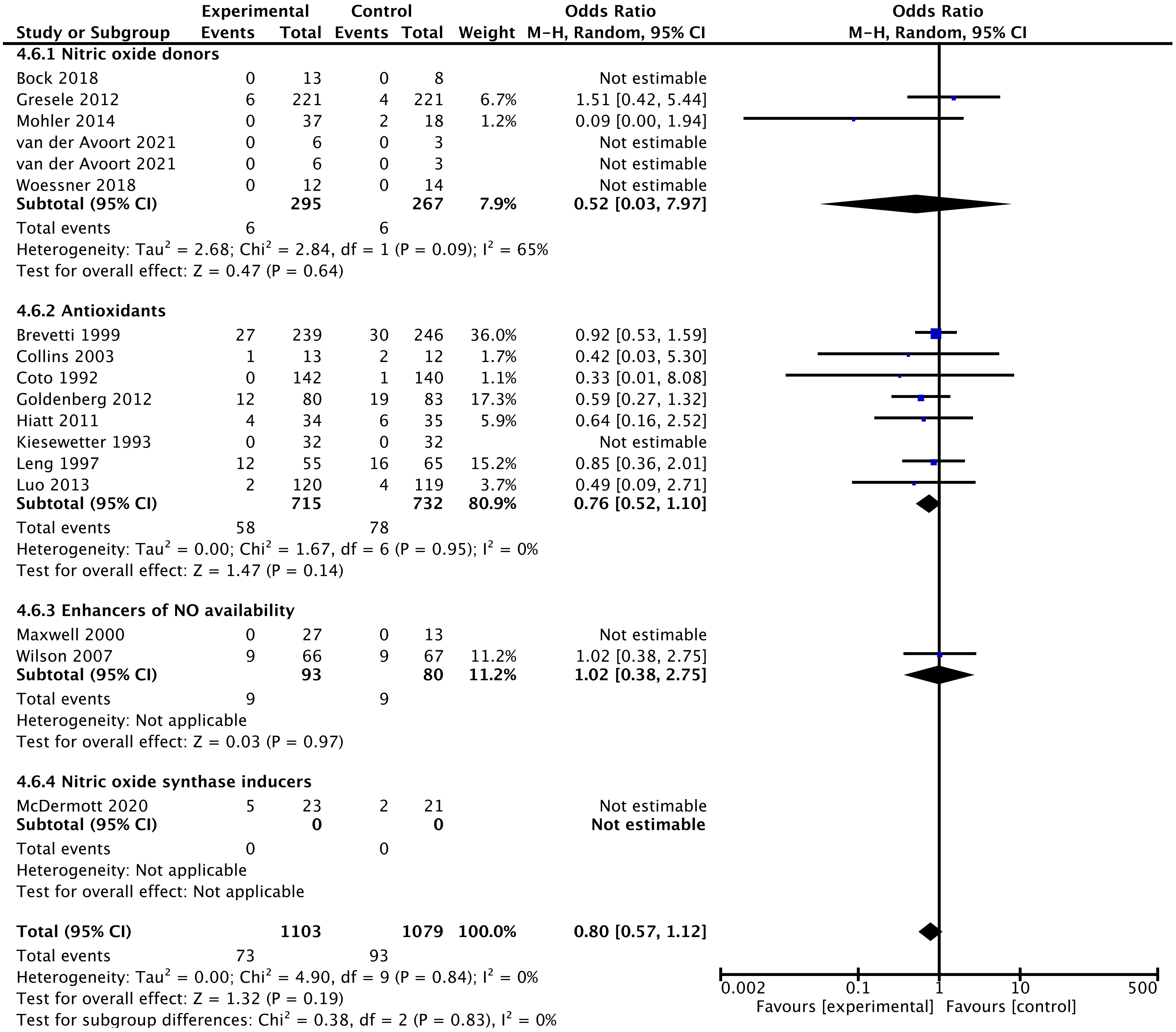

Figure S29. Forest plot showing effect of dietary supplements upregulating the NO pathway versus control on risk of mortality.

Study or Subgroup	Experimental Events	Experimental Total	Control Events	Control Total	Weight	Odds Ratio M-H, Random, 95% CI
Bock 2018	0	13	0	8		Not estimable
Brevetti 1999	5	239	5	246	44.7%	1.03 [0.29, 3.60]
Goldenberg 2012	1	80	0	83	6.8%	3.15 [0.13, 78.49]
Gresele 2012	0	221	1	221	6.8%	0.33 [0.01, 8.19]
Leng 1997	3	55	3	65	26.0%	1.19 [0.23, 6.16]
McDermott 2020	1	23	0	21	6.6%	2.87 [0.11, 74.28]
Ramirez 2019	0	11	0	13		Not estimable
Santo 2006	0	37	0	37		Not estimable
Tenore 2019	0	90	0	90		Not estimable
van der Avoort 2021	0	6	0	3		Not estimable
van der Avoort 2021	0	6	0	3		Not estimable
Vincent 2007	0	16	0	12		Not estimable
Wilson 2007	1	66	1	67	9.0%	1.02 [0.06, 16.58]
Woessner 2018	0	11	0	13		Not estimable
**Total (95% CI)**		**874**		**882**	**100.0%**	**1.14 [0.49, 2.64]**
Total events	11		10			
Heterogeneity: $Tau^2 = 0.00$; $Chi^2 = 1.30$, df = 5 (P = 0.94); $I^2 = 0\%$						
Test for overall effect: Z = 0.31 (P = 0.76)						

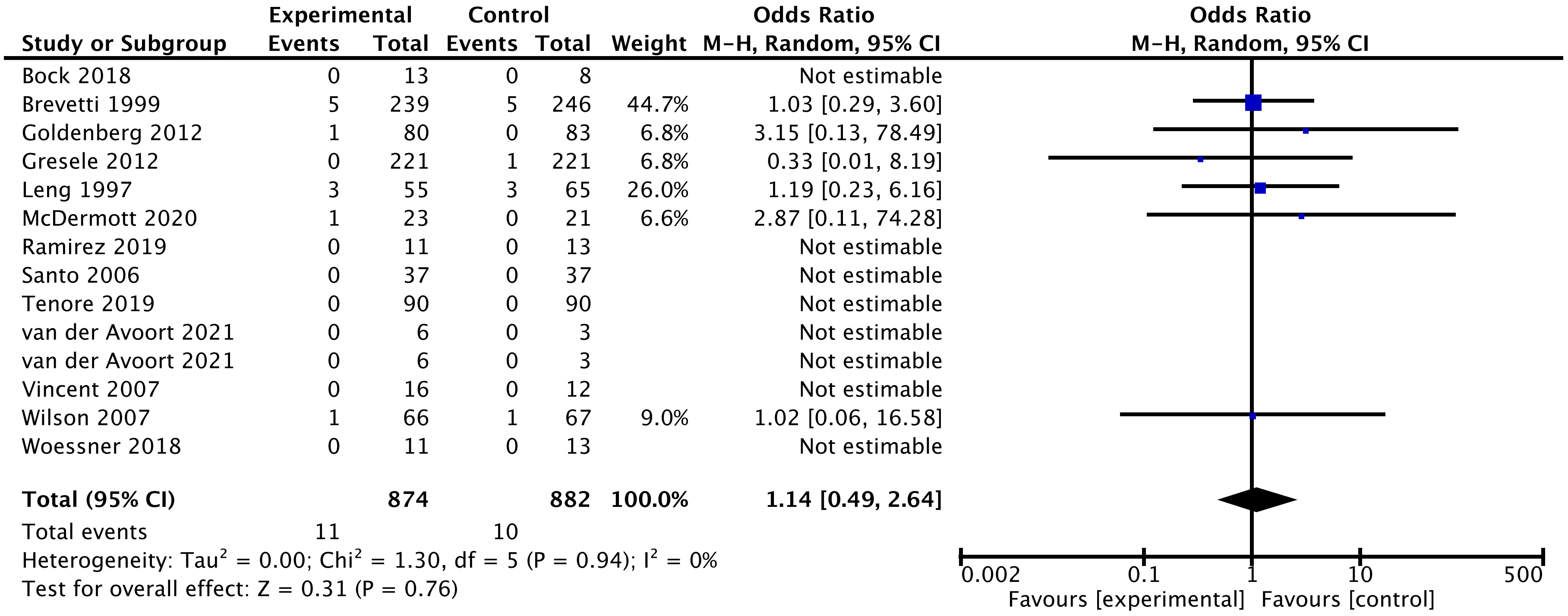

Figure S30. Funnel plot showing the impact of dietary supplements upregulating the NO pathway on risk of mortality.

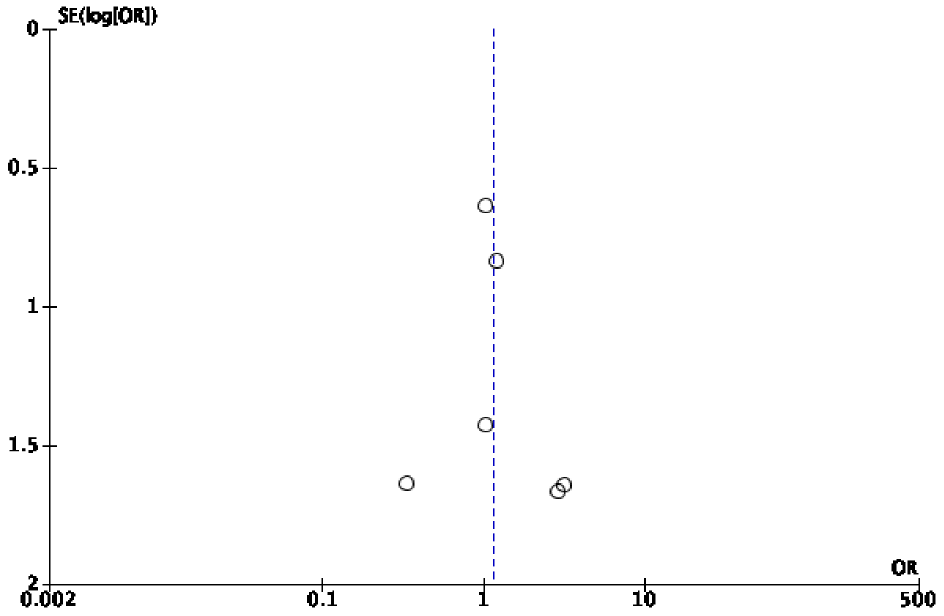

Figure S31. Forest plot showing effect of dietary supplements upregulating the NO pathway versus control on risk of lower extremity revascularisation or amputation.

	Experimental		Control			Odds Ratio	Odds Ratio
**Study or Subgroup**	**Events**	**Total**	**Events**	**Total**	**Weight**	**M-H, Random, 95% CI**	**M-H, Random, 95% CI**
Brevetti 1999	2	239	0	246	21.1%	5.19 [0.25, 108.66]	
Leng 1997	2	55	3	65	58.5%	0.78 [0.13, 4.84]	
McDermott 2020	2	23	0	21	20.4%	5.00 [0.23, 110.40]	
Park 2020	0	5	0	6		Not estimable	
Pekas 2021	0	6	0	5		Not estimable	
van der Avoort 2021	0	6	0	3		Not estimable	
van der Avoort 2021	0	6	0	3		Not estimable	
**Total (95% CI)**		**340**		**349**	**100.0%**	**1.70 [0.42, 6.87]**	
Total events	6		3				

Heterogeneity: $Tau^2 = 0.00$; $Chi^2 = 1.71$, df = 2 (P = 0.42); $I^2 = 0\%$
Test for overall effect: Z = 0.74 (P = 0.46)

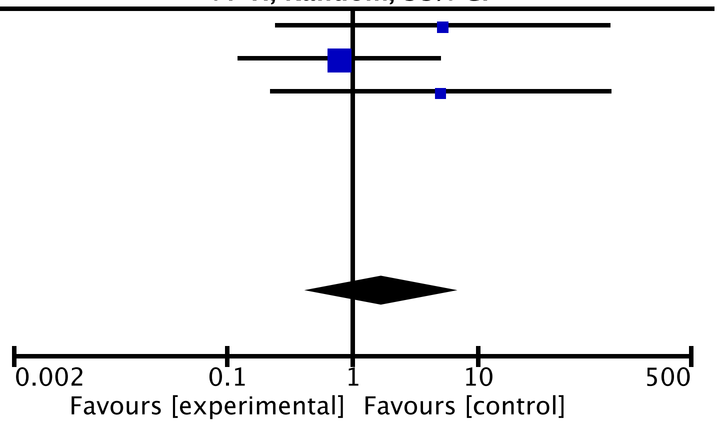

Figure S32. Funnel plot showing the impact of dietary supplements upregulating the NO pathway on risk of lower extremity revascularisation or amputation.

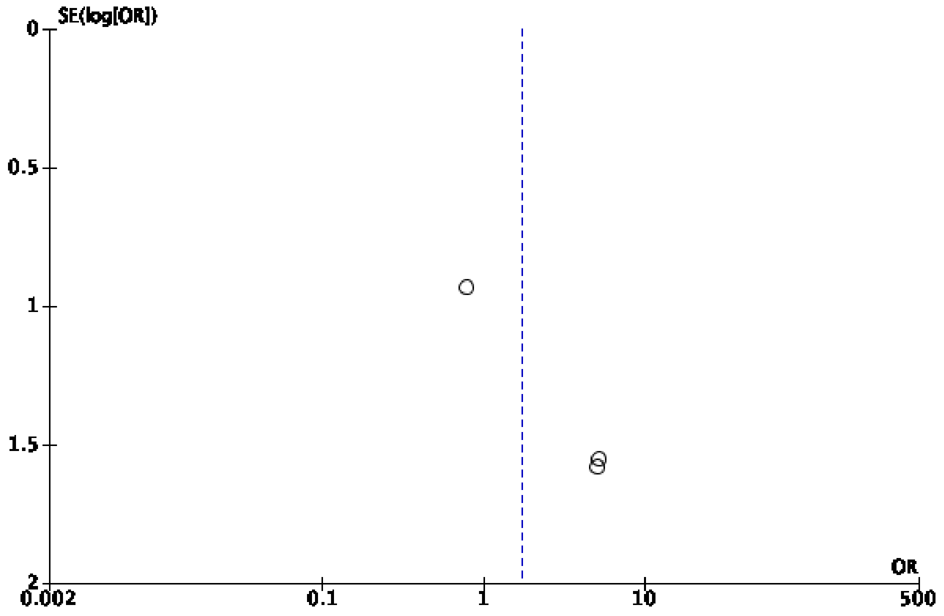

Supplement: Supplementary file 1 [file biomedicines-11-01859-s001.zip › biomedicines-2454548-supplementary.pdf]
